# Ruthenium complexes of 1,4-diazabutadiene ligands with a *cis*-RuCl_2_ moiety for catalytic acceptorless dehydrogenation of alcohols: DFT evidence of chemically non-innocent ligand participation†

**DOI:** 10.1039/d3ra04750d

**Published:** 2023-08-29

**Authors:** Aparajita Mukherjee, Sayanti Datta, Michael G. Richmond, Samaresh Bhattacharya

**Affiliations:** a Department of Chemistry, Inorganic Chemistry Section, Jadavpur University Kolkata 700 032 India samaresh_b@yahoo.com; b Department of Chemistry, Brainware University Kolkata 700 125 India; c Department of Chemistry, University of North Texas Denton TX 76203 USA

## Abstract

The acceptorless dehydrogenative coupling (ADC) of primary alcohols to esters by diazabutadiene-coordinated ruthenium compounds is reported. Treatment of *cis*-Ru(dmso)_4_Cl_2_ in acetone at 56 °C with different 1,4-diazabutadienes [*p*-XC_6_H_4_N=C(H)(H)C=NC_6_H_4_X-*p*; X = H, CH_3_, OCH_3_, and Cl; abbreviated as DAB-X], gives *trans*-Ru[κ^2^-*N*,*N*-DAB-X]_2_Cl_2_ as the kinetic product of substitution. Heating these products in *o*-xylene at 144 °C gives the thermodynamically favored *cis*-Ru[κ^2^-*N*,*N*-DAB-X]_2_Cl_2_ isomers. Electronic structure calculations confirm the greater stability of the *cis* diastereomer. The molecular structures for each pair of geometric isomers have been determined by X-ray diffraction analyses. Cyclic voltammetry experiments on the complexes show an oxidative response and a reductive response within 0.50 to 0.93 V and −0.76 to −1.24 V *vs.* SCE respectively. The *cis*-Ru[κ^2^-*N*,*N*-DAB-X]_2_Cl_2_ complexes function as catalyst precursors for the acceptorless dehydrogenative coupling of primary alcohols to H_2_ and homo- and cross-coupled esters. When 1,4-butanediol and 1,5-pentanediol are employed as substrates, lactones and hydroxyaldehydes are produced as the major dehydrogenation products, while secondary alcohols afforded ketones in excellent yields. The mechanism for the dehydrogenation of benzyl alcohol to benzyl benzoate and H_2_ using *cis*-Ru[κ^2^-*N*,*N*-DAB-H]_2_Cl_2_ (*cis*-1) as a catalyst precursor was investigated by DFT calculations. The data support a catalytic cycle that involves the four-coordinate species Ru[κ^2^-*N*,*N*-DAB-H][κ^1^-*N*-DAB-H](κ^1^-OCH_2_Ph) whose protonated κ^1^-diazabutadiene moiety functions as a chemically non-innocent ligand that facilitates a β-hydrogen elimination from the κ^1^-*O*-benzoxide ligand to give the corresponding hydride HRu[κ^2^-*N*,*N*-DAB-H][κ^1^-*N*-DAB-H](κ^2^-*O*,*C*-benzaldehyde). H_2_ production follows a Noyori-type elimination to give (H_2_)Ru[κ^2^-*N*,*N*-DAB-H][κ^1^-*N*-DAB-H](κ^1^-*O*-benzaldehyde) as an intermediate in the catalytic cycle.

## Abstract 2

A family of diazabutadiene bound ruthenium compounds bearing a RuCl_2_ moiety has been synthesized, and catalytic efficiency of their *cis*-isomer for the coupling of primary alcohols to esters has been studied.

## Graphical abstract

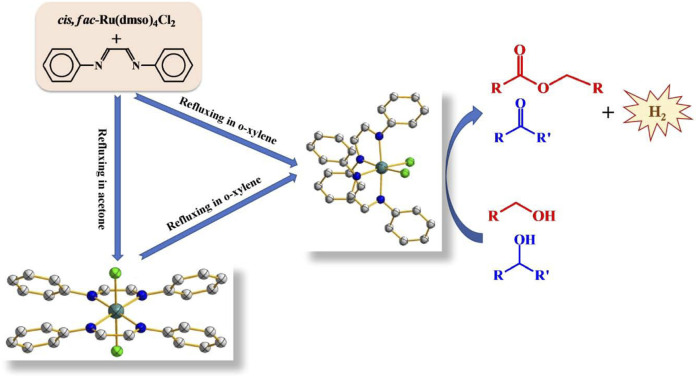

## Introduction

Ruthenium-promoted chemistry continues to attract worldwide attention from both the academic and industrial sectors. From the catalysis of key commodity chemicals to their use as chemotherapeutic agents,^1–4^ ruthenium compounds play a pivotal role in helping to address some of the critical challenges faced by the consumer products industry. Of the many different types of ruthenium compounds known, those having a *cis*-RuCl_2_ moiety are of particular interest in the catalysis and biomedical fields. Interest in ruthenium(ii) complexes with a *cis*-RuCl_2_ moiety remains high, with attention directed toward their use in the preparation of new complexes,^2^ as efficient DNA binding and anti-tumor agents,^3^ and as catalyst precursors for a variety of organic transformations.^4^ The vast majority of these applications rely on the enhanced lability of the Ru–Cl bond, which, in turn, is influenced by the nature of the other ligands in the primary coordination sphere of the ruthenium.^5^ The coordination of ruthenium by π-acid ligands is fundamental for the stabilization of the ruthenium(ii) oxidation state, and such ligands also augment the lability of the Ru–Cl bonds in *cis*-Ru(α-diimine)_2_Cl_2_ and related complexes to furnish syn coordination sites in a well-defined molecular environment that synergistically act on organic substrates to effect site-selective activation and value-added functionalization.^6,7^ We maintain an interest in this genre of compounds and have published several papers on the synthesis and the catalytic activity of new nitrogen- and phosphine-coordinated ruthenium complexes possessing a reactive *cis*-RuCl_2_ moiety in transfer hydrogenation and acceptorless dehydrogenative coupling reactions.^8^

**Scheme 1:**
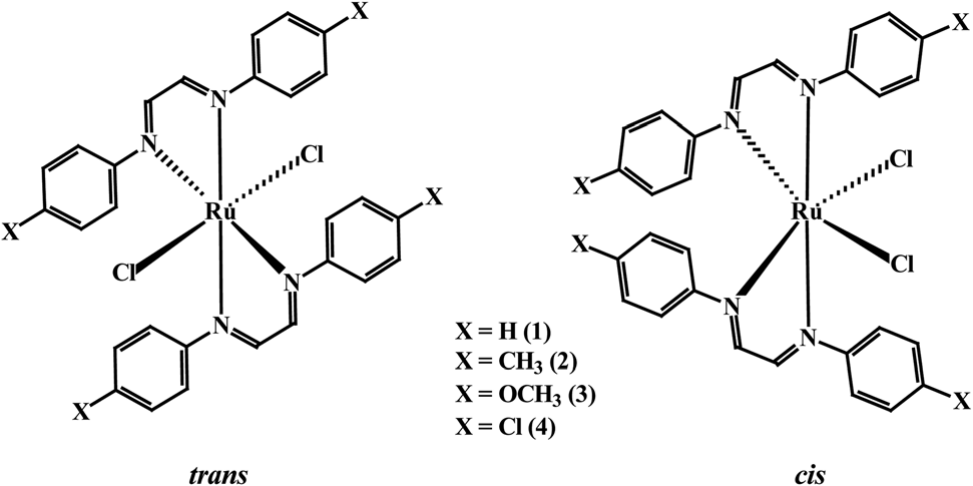
1,4-Diazabutadiene-coordinated ruthenium(ii) compounds 1–4 investigated.

Wishing to branch out and to explore different ruthenium precursors as catalysts for the directed synthesis of complex organic molecules, we have initiated studies on a series of α-diimine-coordinated ruthenium compounds with the common formula *cis*-Ru[κ^2^-*N*,*N-p*-XC_6_H_4_N=C(H)(H)C=NC_6_H_4_X-*p*]_2_Cl_2_, where the *p*-XC_6_H_4_N=C(H)(H)C=NC_6_H_4_X-*p* ligand is based on the 1,4-diazabutadiene (DAB) platform (Scheme 1). These nitrogen donors are easily prepared from glyoxal and *para*-substituted anilines and they typically function as κ^2^-*N*,*N*-donors to give five-membered chelate rings.^9^ Numerous electrochemical and photophysical studies have demonstrated notable π-acidity of the κ^2^-bound *p*-XC_6_H_4_N=C(H)(H)C=NC_6_H_4_X-*p* ligands,^10^ a property that also promotes the stabilization of metals in low oxidation states; this latter feature is catalytically desirable and remains an important consideration in the design of late-metal catalysts for hydrogenation and C–C bond coupling reactions.^11^

Interest in the catalytic conversion of primary alcohols to esters by acceptorless dehydrogenative coupling (ADC) remains high compared to traditional synthetic methods for ester formation from alcohols (eqn (1)).^12^ ADC catalysis is environmentally friendly, and it1
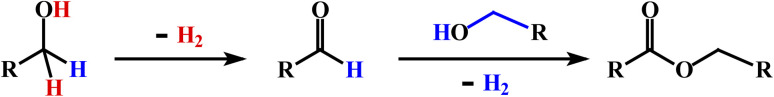
aligns itself nicely with our current emphasis on green catalysis, not to mention ADC chemistry is atom-economical in nature.^13,14^ The coupling of two alcohols to furnish an ester in the absence of stoichiometric oxidizing reagents remains a priority in the field of catalysis. Herein we present our results on the synthesis and catalytic properties in the acceptorless dehydrogenative coupling (ADC) of alcohols to esters by new 1,4-diazabutadiene-coordinated compounds *cis*-Ru[κ^2^-*N*,*N-p*-XC_6_H_4_N=C(H)(H)C=NC_6_H_4_X-*p*]_2_Cl_2_. The catalytic mechanism for the observed ADC reaction of benzyl alcohol to benzyl benzoate and H_2_ was investigated by electronic structure calculations, and a mechanism involving Ru(0) intermediate and a chemically non-innocent κ^1^-*N-p*-XC_6_H_4_N=C(H)(H)C=N(H)C_6_H_4_X-*p* ligand, which is formed by hydrogen transfer from the hydrido-alkoxide intermediate *cis*-HRu[κ^2^-*N*,*N-p*-XC_6_H_4_N=C(H)(H)C=N(H)C_6_H_4_X-*p*]_2_(κ^1^-OCH_2_Ph), is discussed.

## Results and discussion

### Syntheses and characterization

**Fig. 1:**
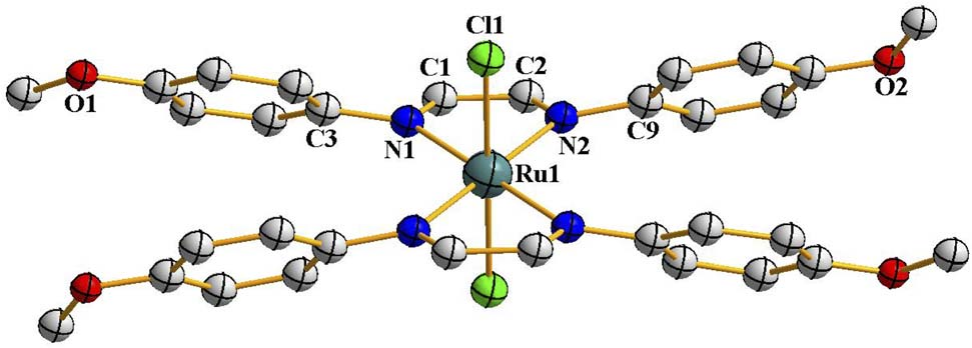
Molecular structures of *trans*-3. The thermal ellipsoids are drawn at the 50% probability level. Hydrogen atoms have been omitted for clarity.

As delineated in the introduction, in order to explore the catalytic efficiency of *cis*-Ru(DAB-X)_2_Cl_2_ in acceptorless dehydrogenation of alcohols, we prepared a series of Ru(DAB-X)_2_Cl_2_ compounds 1–4 (Scheme 1). Treatment of the *cis*-Ru(dmso)_4_Cl_2_ with a slight excess of two equivalents of DAB-X in refluxing acetone afforded a green complex having general formula Ru(DAB-X)_2_Cl_2_ in good yield. Use of the relatively low boiling solvent (acetone) for the synthetic reaction was intended for retaining the *cis*-RuCl_2_ fragment of the *cis*-Ru(dmso)_4_Cl_2_ starting compound in the final bis-chelated product. The crystal structures of the green products authenticated the *trans*-geometry of the Ru(DAB-X)_2_Cl_2_ complexes. Structure of *trans*-Ru(DAB-OCH_3_)_2_Cl_2_ (*trans*-3) is displayed in Fig. 1. The structure clearly shows that the two DAB-OCH_3_ ligands are coordinated to ruthenium, and have constituted an equatorial plane with ruthenium at the center with two *trans* coordinated chlorides (Scheme 1). The molecular structures of the other three green products, *viz. trans*-1, *trans*-2 and *trans*-4, are similar to that of *trans*-3, and these are deposited as Fig. S1 (ESI†). ^1^H NMR data of the green Ru(DAB-X)_2_Cl_2_ complexes are also consistent with the *trans*-geometry.

As DAB-X ligands are symmetric in nature, the Ru(DAB-X)_2_Cl_2_ complexes can, in principle, exist in two geometrical isomeric forms, *viz. cis* and *trans*, in view of relative disposition of the two coordinated chlorides (Scheme 1). Formation of the *trans*-isomer from *cis*-Ru(dmso)_4_Cl_2_ in boiling acetone was quite intriguing, particularly as Ru(diimine)_2_Cl_2_ type complexes are well known to prefer the *cis*-geometry for better π-interaction.^7,15^ The difference in thermodynamic stability of the *cis* and *trans* isomers of Ru(DAB-X)_2_Cl_2_, complexes was investigated by DFT calculations. For each complex, the *cis*-isomer was found to be more stable than the *trans*-isomer (Fig. S2, (ESI†)). The fact, that in spite of the greater thermodynamic stability of the *cis*-isomer, the formation of *trans*-isomer in the refluxing acetone indicates that the *trans*-isomer is probably the kinetically controlled product. Conversion of *trans*-isomer to the more stable *cis*-isomer could not take place in refluxing acetone, presumably due to unavailability of adequate energy required for the isomerization.

**Fig. 2:**
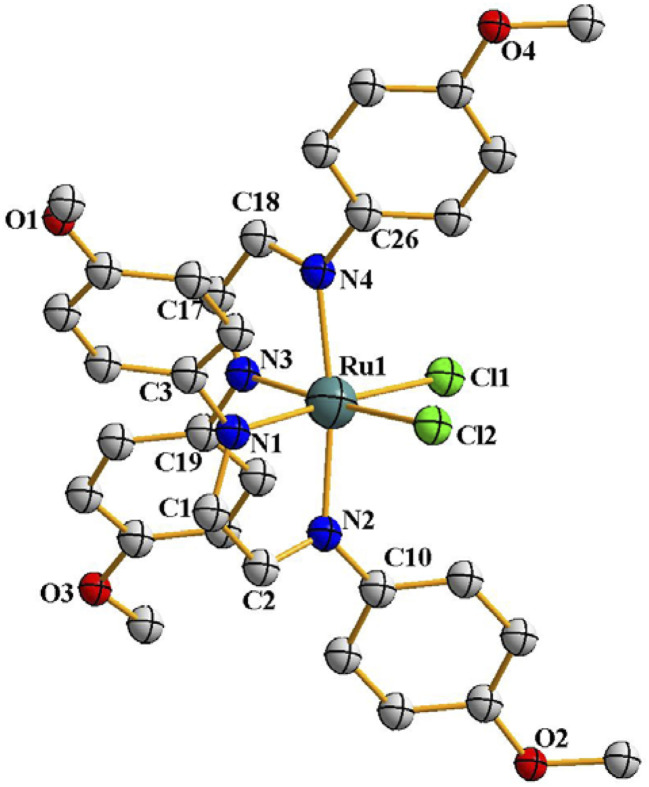
Molecular structures of *cis*-3. The thermal ellipsoids are drawn at the 50% probability level. Hydrogen atoms have been omitted for clarity.

**Scheme 2:**
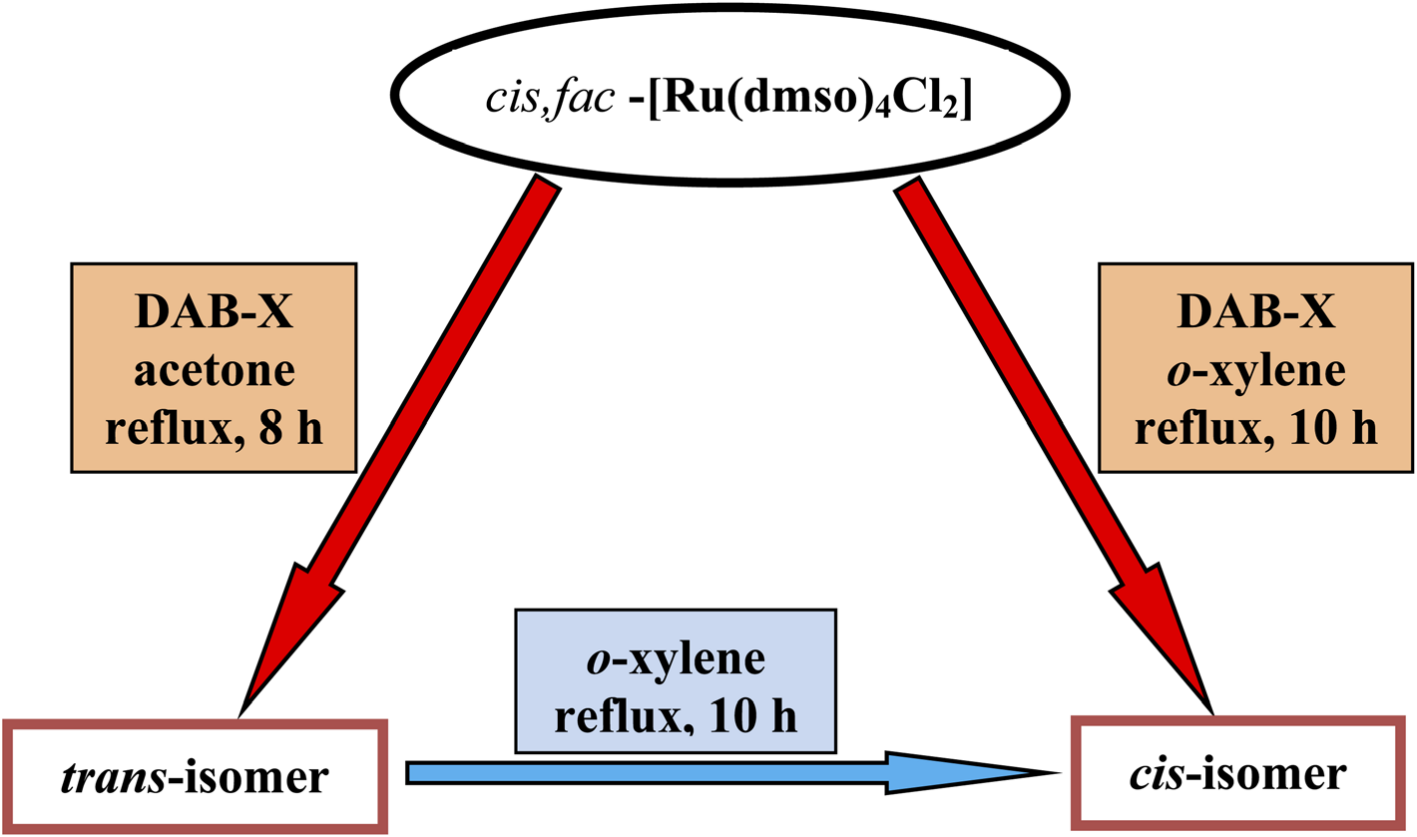
Formation of *trans*- and *cis*-isomers, and conversion of *trans* → *cis*.

Following the hint from the energy calculations, reaction of DAB-X with *cis*-[Ru(dmso)_4_Cl_2_] was next carried out in a relatively high-boiling solvent, *viz. ortho*-xylene, under refluxing condition, which afforded blue (X = H, Me, Cl) or bluish-green (X = OCH_3_) product having the same general formula Ru(DAB-X)_2_Cl_2_. Crystal structures of these complexes, as well as their ^1^H NMR spectra, confirmed the *cis*-geometry of them. Molecular structure of *cis*-3 is shown in Fig. 2 as a representative, and structures of the remaining three complexes (*cis*-1, *cis*-2 and *cis*-4) are shown in Fig. S3 (ESI†). In summary, heating the DAB-X and *cis*-[Ru(dmso)_4_Cl_2_] in acetone gives the *trans* isomer as the kinetically controlled product, while refluxing the starting reagents in *o*-xylene affords the thermodynamically favored *cis* isomer. The isolated *trans*-isomers are also found to be quantitatively convertible into the corresponding *cis*-isomers by simply heating the former in refluxing *ortho*-xylene.^16^ The solvent dependent formation of *trans*- and *cis*-isomers and conversion of trans → cis are summarized in Scheme 2. The observed trans → cis isomerization in each group of isomers proceeds by a unimolecular reaction that is believed to involve a Bailar-twist process through a trigonal prismatic transition state.^17,18^

The isomers of complexes 1–4 were characterized by elemental analysis and traditional spectroscopic methods, and these data are summarized in the Experimental section. Examination of the known structures on deposit with the Cambridge Crystallographic Data Centre (CSD version 5.40) reveals only three entries with the general formula Ru(DAB-X)_2_Cl_2_ of which two structures are polymorphs. The deposited structures are consistent with the formulated composition for each isomer and do not exhibit any significant differences in their structural parameters. Reports on the synthesis of isomers of compounds 2 and 3 by methods significantly different from ours exist in the literature.^17,19^

The structures depicted in Fig. 1, 2, S1 and S3 (ESI†) confirm that the two 1,4-diazabutadiene ligands chelate the ruthenium in the expected fashion (Scheme 1). The observed Ru–Cl and Ru–N distances in each isomer compare well with those found earlier.^19–21^ The ruthenium center is six-coordinate in each structure, and the subtended angles at the metal are consistent with the arrangement of the ligands in each isomer. The C–C and C–N bond lengths within the chelating DAB-X ligands in *trans*- and *cis*-complexes deviate significantly from localized C–C single bond and C=N double bond distances in keeping with an enhanced delocalization of the π-cloud and back-donation from the electron-rich, low-spin d^6^ ruthenium center.

### Spectral and electrochemical properties

The ^1^H NMR spectral data of the complexes are presented in the Experimental section. Number of signals observed in each of the *trans* isomers is consistent with the presence of two C_2_ axes. For example, only one signal near 8.6 ppm is observed for the four azomethine protons. In each of the *cis* isomers, two azomethine proton signals were found within 8.1–8.6 ppm, indicating existence of only one C_2_ axis.

Electronic spectra of the complexes were recorded in dichloromethane solutions, and the spectral data are given in the Experimental section. Each complex shows two intense absorptions in the visible region, which are tentatively attributed to metal-to-ligand (DAB-X) charge-transfer transitions. The intense absorptions in the ultraviolet region are assignable to transitions within the DAB-X ligand orbitals.

Redox activity of the *trans*- and *cis*-Ru(DAB-X)_2_Cl_2_ complexes was examined in acetonitrile solution (0.1 M TBHP) by cyclic voltammetry. Each complex shows a reversible oxidation within 0.5 to 1.0 V and an irreversible reduction within −0.7 to −1.3 V (all potentials are referenced to SCE). Voltammetric data are presented in Table S9 (ESI†), and a representative CV is shown in Fig. S4 (ESI†). The oxidative response is assigned to Ru(ii)–Ru(iii) oxidation. In the *trans*-isomers the Ru(ii)–Ru(iii) oxidation occurs at a much lower (*ca.* 300 mV) potential compared to that in the corresponding *cis* isomers, which is attributed to difference in stereochemical preference of Ru(ii) and Ru(iii).^7,15,22^ In *cis*-Ru(bpy)_2_Cl_2_, an exhaustively studied complex, the Ru(ii)–Ru(iii) oxidation takes place at 0.29 V,^23^ while in the *cis*-Ru(DAB-X)_2_Cl_2_ complexes the same oxidation occurs at *ca.* 0.8 V. The observed positive shift of about 500 mV indicates that these 1,4-diazabutadiene ligands are better π-acids than bpy.

The Ru(ii)–Ru(iii) oxidation potential in the *trans*- and *cis*-Ru(DAB-X)_2_Cl_2_ complexes is found to be sensitive to the nature of substituent X in the DAB-X ligand. The potential increases with increasing electron-withdrawing character of X. The plot of oxidation potentials *vs.* Hammett constant of the substituents (4*σ*; the *σ* values being:^24^ OCH_3_ = −0.27, CH_3_ = −0.17, H = 0.00 and Cl = 0.23) is linear for both *trans*-[Ru(DAB-X)_2_Cl_2_] and *cis*-[Ru(DAB-X)_2_Cl_2_] complexes (Fig. S5 (ESI†)). The observed slope (slope = reaction constant (*ρ*) of this redox couple^25^) is 0.09 V for the *trans*-complexes and 0.12 V for the *cis*-complexes. From these linear correlations with reasonable slopes it is clear that the substituents on the 1,4-diazabutadiene ligands, which are six bonds away from the electroactive metal center, can influence the redox potential in a predictable manner. The irreversible reductive response is attributed to reduction of the α-diimine fragment in the 1,4-diazabutadiene ligands. Potential of this reduction does not show any systematic variation with the nature of substituent X.

### Catalytic acceptorless dehydrogenation of alcohols

As outlined in the introduction, the other major objective of this study was to explore the catalytic activity of a series of *cis*-Ru(DAB-X)_2_Cl_2_ complexes in acceptorless dehydrogenation of alcohols. The high efficiency of catalyst precursors with a *cis*-RuCl_2_ moiety [or *cis*-Ru(H)Cl or *cis*-Ru(H)_2_ moieties derived from a *cis*-RuCl_2_ unit] in fundamental bond-activation reactions is well-documented in the literature.^12,13^ We began our study by examining the dehydrogenation of benzyl alcohol using 0.1 mol% *cis*-Ru(DAB-X)_2_Cl_2_ as the catalyst precursor in toluene as a solvent. These data are summarized in Table S10 (ESI†), and surprisingly, instead of observing benzaldehyde as the dominant product of dehydrogenation, benzyl benzoate was obtained in relatively good yield [entry 1, Table S10 (ESI†)].^26^ The presence of H_2_ was verified by ^1^H NMR spectroscopy [Fig. S6 (ESI†)].^27^ The coupling of benzyl alcohol to furnish benzyl benzoate in the absence of an added oxidant or sacrificial hydrogen acceptor is, though precedent,^14*g*,*i*^ quite interesting. The formation of benzyl benzoate and H_2_ as reaction products proceeds by an initial dehydrogenation of benzyl alcohol, followed by a dehydrogenative C–O bond coupling involving the parent alcohol and benzaldehyde (eqn (1)). Having established the ADC reaction with benzyl alcohol using *cis*-1, we next investigated the catalytic activity of the other *cis* isomers based on compounds 2–4; these data are found in entries 13–15 in Table S10 (ESI†). The three other catalyst precursors also give benzyl benzoate as the major product in yields that range from 66% (*cis*-4) to 43% (*cis*-3). The presence of small amounts of benzaldehyde (<15%) was confirmed in each of these reactions. Qualitatively, the *para* substituent in the four catalysts investigated mildly influences the overall yield of product ester, with the electron-donating Me and MeO groups in *cis*-2 and *cis*-3 affording lower yields of benzyl benzoate relative to the phenyl and *para*-chloro derivatives. Of the four different catalyst precursors examined, *cis*-1 was the most active, and the optimal reaction conditions using *cis*-1 were ultimately finalized as listed in entry 2 of Table S10 (ESI†). Accordingly, we selected *cis*-1 for more detailed screening studies with other alcohol substrates, the results of which are presented below.

The scope of the dehydrogenation using different primary alcohols and *cis*-1 as a catalyst was next investigated using the optimized conditions from the benzyl alcohol reaction [entry 2; Table S10 (ESI†)]. Five additional primary alcohols were examined as substrates, and the catalysis results are reported in Table 1. All of the primary alcohols examined furnished the corresponding ester as the major dehydrogenated product. The yield of ester yield ranged from good (51% for 1-butanol) to excellent (90% for isobutyl alcohol), with the corresponding aldehyde present in yields that ranged from a high of 18% (1-butanol) to a low of 1% (isobutyl alcohol). Though relatively less common, several reports concerning the ruthenium-catalyzed formation of esters *via* ADC of alcohols exist.^14*a*,*e*,*g*–*i*^

**Table:** Dehydrogenation of primary alcohols^a^

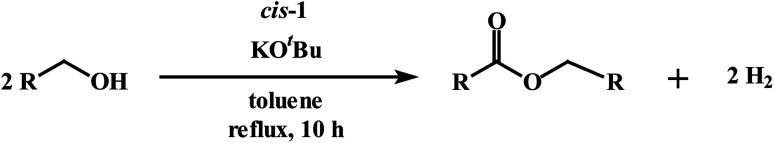		
Entry	Substrate	Yield,^b^ %
1	Benzyl alcohol	74 (73)^c^
2	Ethanol	82 (80)^c^
3	1-Propanol	81
4	1-Butanol	51
5	Isoamyl alcohol	86 (85)^c^
6	Isobutyl alcohol	90

a Reaction conditions: primary alcohol (1.0 mmol), KO^*t*^Bu (1.0 mol%), *cis*-1 catalyst (0.1 mol%), 1 : 4 dichloromethane–toluene (5 mL).

b Determined by GCMS.

c Yield from reaction carried out under argon atmosphere.

We next explored the efficacy of *cis*-1 in the cross-coupling of two different alcohols to furnish mixed-ester products (eqn (2)) since transition-metal catalyzed dehydrogenative cross-coupling reactions are few in number. A recent report by Milstein and coworkers confirms the successful cross-coupling of primary alcohols to form cross-esters using a manganese pincer complex.^28^Table 2 summarizes our ester cross-coupling results. We employed the standard reaction conditions adopted for the benzyl alcohol cross-coupling [entry 2, Table S10 (ESI†)] and here we used a 1 : 1 mixture of primary alcohols. In principle, four different esters may be2
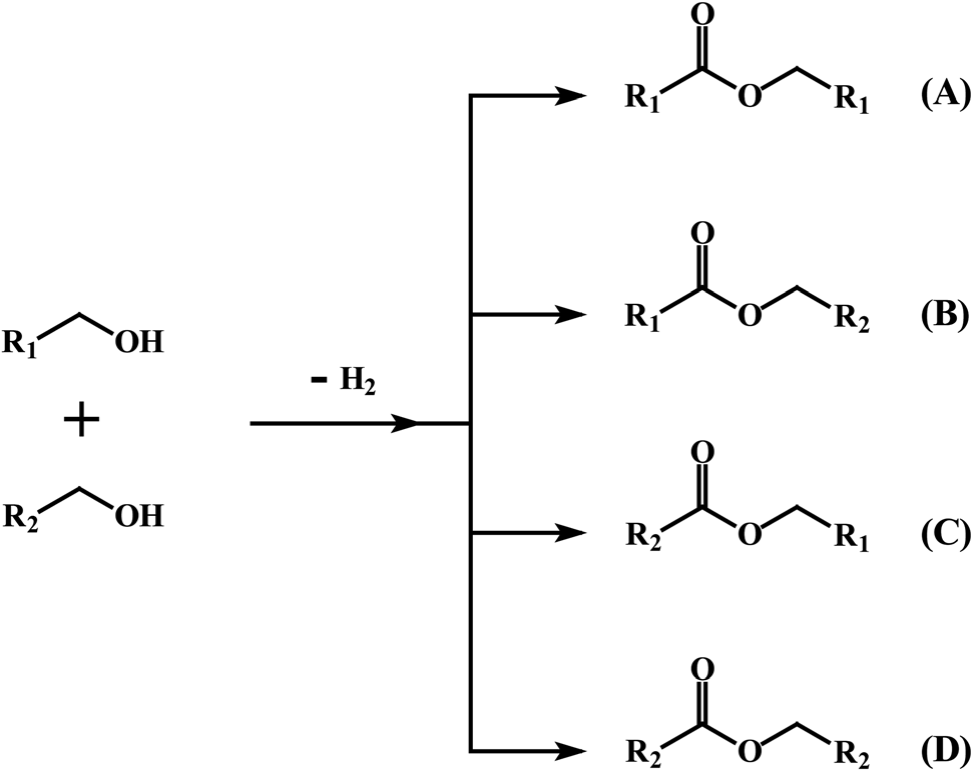
produced, as illustrated by the reaction depicted in the header of Table 2. However, only the reaction using ethanol and benzyl alcohol afforded all four possible ester products (entry 5). The remaining reactions gave their respective homo-esters and only one of the two cross-coupled esters as observed products (entries 1–4). The observed variation in product distribution is, presumably, due to difference in bulk of the alkyl/aryl moieties (R and R′) in the alcohols, which, in turn, influences approach of these substrates to the metal center in the catalyst, and hence the reaction kinetics. We also examined the ADC reaction using the terminal diols 1,4-butanediol and 1,5-pentanediol and found the cyclized products γ-butyrolactone, and δ-valerolactone, respectively, as the major product. Accompanying each lactone was the corresponding hydroxyaldehyde. The data from the diol reactions support a scheme that involves initial oxidation to give a hydroxyaldehyde whose intramolecular cyclization is slightly faster (∼2×) compared to the dissociation of the initial oxidation product from the κ^1^-aldehyde intermediate. The observed lactone products are in keeping with the Ru-catalyzed lactone formation from terminal diols reported earlier by the groups of Szymczak^14*g*^ and Hartwig.^14*j*^

Dehydrogenative cross-coupling of two primary alcohols^a^

**Table:** 

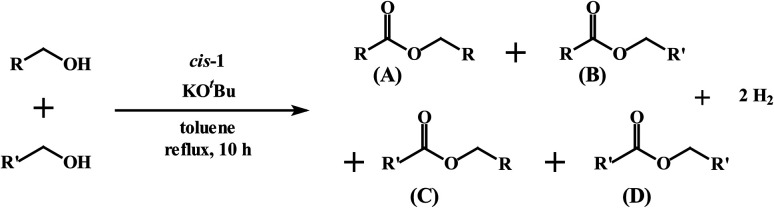						
Entry	R	R′	Yield^b^A (%)	Yield^b^B (%)	Yield^b^C (%)	Yield^b^D (%)
1	CH_3_	CH_3_CH_2_	38	15	No	40
2	CH_3_	C_6_H_5_	43	36	No	No
3	C_6_H_5_	4-OCH_3_-C_6_H_4_	42	23	12	No
4	C_6_H_5_	4-Cl-C_6_H_4_	39	28	15	No
5	CH_3_CH_2_	C_6_H_5_	21	8	35	27

a Reaction conditions: primary alcohol (1.0 mmol), KO^*t*^Bu (1.0 mol%), *cis*-1 catalyst (0.1 mol%), 1 : 4 dichloromethane–toluene (5 mL).

b Determined by GCMS.

**Table:** 

Dehydrogenation of terminal diols			
	Diol	Product-I^b^	Product-II^b^
6	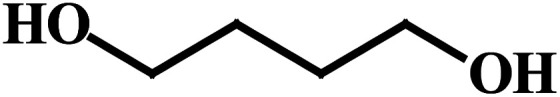	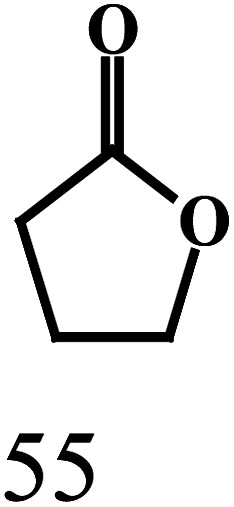	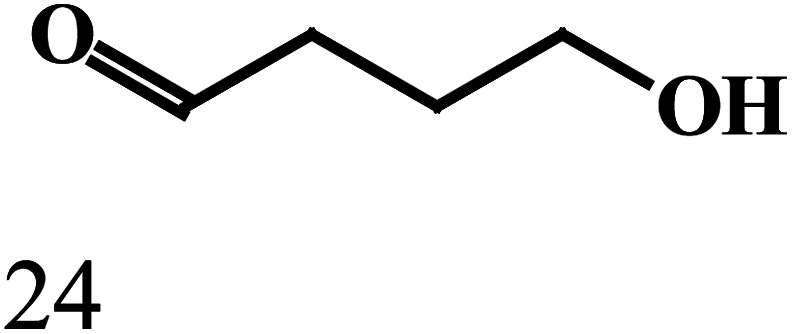
7	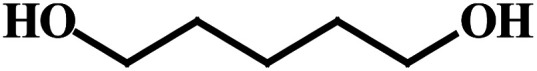	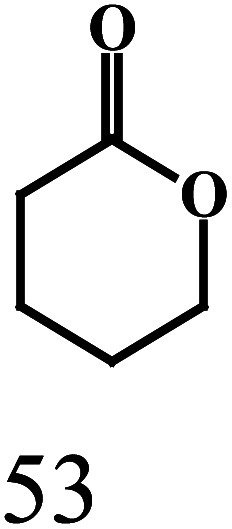	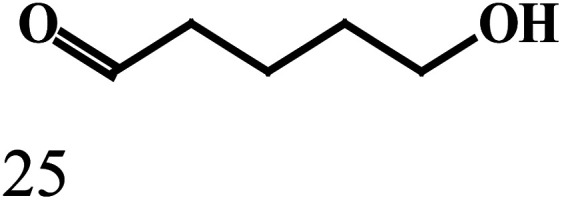

Finally, the dehydrogenation of selected secondary alcohols was investigated using *cis*-1 (eqn (3)). Similar experimental conditions, as in the case of primary alcohols, were used,3
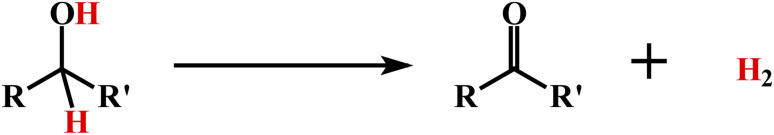
except that the catalyst loading had to be increased to 0.2 mol% for a better yield. The yield of ketone product was lower by a factor of two with a 0.1 mol% catalyst loading. Table 3 shows the results of our dehydrogenation of secondary alcohols. The expected ketone was obtained in excellent yield in each case, and the evolution of H_2_ was again verified by ^1^H NMR studies (Fig. S7 (ESI†)). Control experiments, carried out under an argon atmosphere, afforded the same ketone in comparable yield (*cf.*: entries 1 and 6), serving to eliminate the participation molecular oxygen in the alcohol oxidation. As expected, no ester product was found in any of these reactions that employed a secondary alcohol as the substrate. The observed catalytic efficiency of *cis*-1 towards acceptorless dehydrogenation of alcohols is better than that of many other Ru-catalysts,^14*b*–*d*,*f*,*g*,*i*,*j*^ and comparable to one reported Ru-complex.^14*a*^

**Table:** Dehydrogenation of secondary alcohols^a^

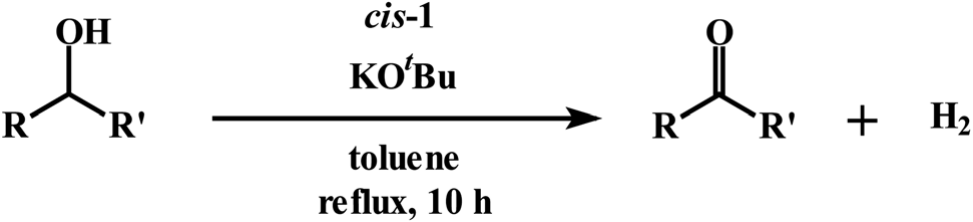		
Entry	Reactant	Yield^b^, %
1	Cyclohexanol	86 (83)^c^
2	2-Propanol	90
3	2-Butanol	88
4	3-Methyl-2-butanol	83
5	Cyclopentanol	89
6	Benzhydrol	92 (90)^c^

a Reaction conditions: secondary alcohol (1.0 mmol), KO^*t*^Bu (0.1 mol%), *cis*-1 catalyst (0.2 mol%), 1 : 4 dichloromethane–toluene (5 mL).

b Determined by GCMS.

c Yield from reaction carried out under argon atmosphere.

### Mechanism for the acceptorless dehydrogenative coupling reaction

**Scheme 3:**
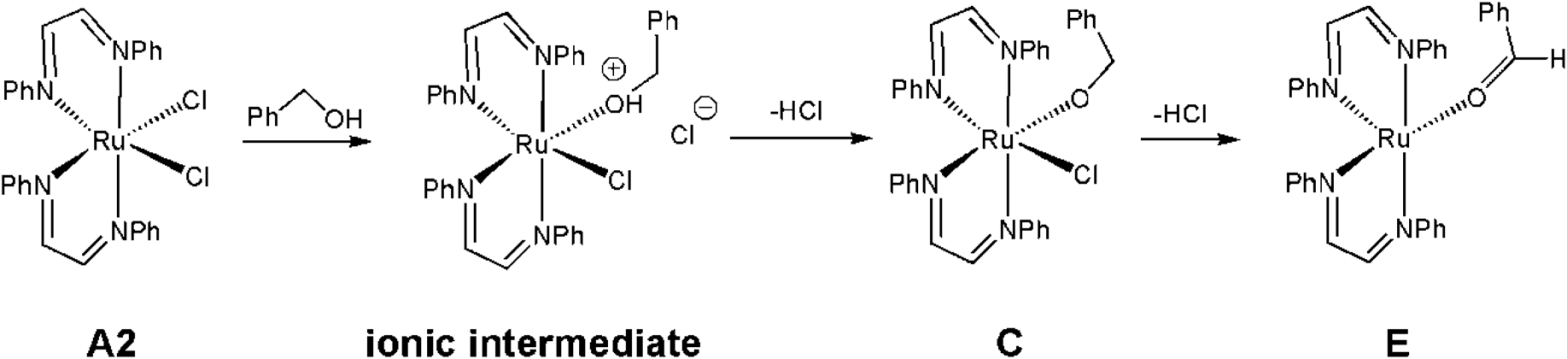
Proposed path for the alcohol-promoted Ru(ii) → Ru(0) process starting from *cis*-1.

The ruthenium-mediated formation of benzyl benzoate requires, at a minimum, two successive catalytic steps involving the dehydrogenation of benzyl alcohol to benzaldehyde, followed by C–O coupling with additional alcohol. A likely contributor to the catalytic cycle is the unsaturated ruthenium(0) intermediate Ru(DAB-H)_2_, whose formation may be traced to an initial reduction process with the alcohol substrate serving as the reducing agent in the Ru(ii) → Ru(0) process. Generation of ruthenium(0) species bearing soft ligands is precedent in literature.^29^Scheme 3 shows a sequence of events that serves to convert *cis*-1 to the κ^1^-*O*-benzaldehyde complex E. The activation of *cis*-1 by the alcohol substrate was experimentally examined using ethanol at room temperature. Treatment of *cis*-1 with ethanol led to a slow increase in the solution conductivity (*Λ* = 12.0 S cm^2^ mol^−1^) over the course of several hours in support of the ion pair [Ru(DAB-H)_2_(EtOH)Cl][Cl].^30^ While not examined, deprotonation of the ionic intermediate produced from benzyl alcohol would give the corresponding chloro-alkoxide species C, which upon loss of additional HCl would furnish the κ^1^-*O*-benzaldehyde complex E. Dissociation of the aldehyde from the latter species or its reaction with additional alcohol to give the homocoupled-ester product would ultimately furnish the key catalytic intermediate Ru(DAB-H)_2_. The proposed reduction pathway depicted in Scheme 3 is strengthened by reports on glycol reduction of related ruthenium(ii) precursors to active ruthenium(0) species used in C–H and O–H bond activation reactions.^31^

**Fig. 3:**
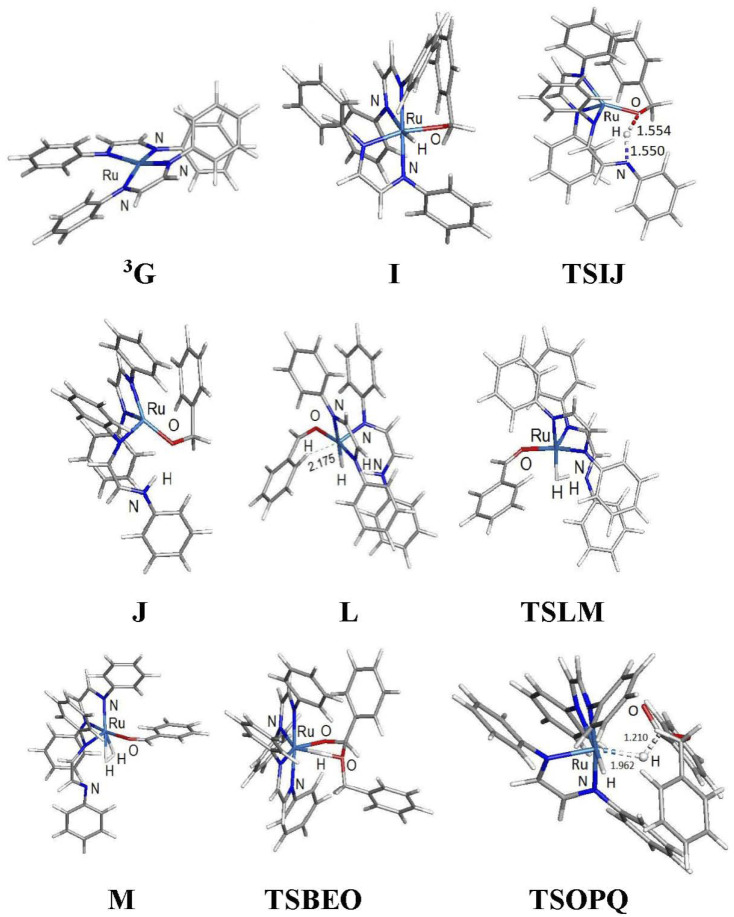
DFT-optimized structures for selected participants in the ADC reaction mechanism depicted in Fig. 4. Displayed bond distances for species TSIJ, L, and TSOPQ are in Å.

Before addressing the mechanism of the acceptorless dehydrogenative C–O coupling reaction involving *cis*-1 and benzyl alcohol, we wished to establish the structure of the unsaturated intermediate Ru(DAB-H)_2_ by DFT calculations. As a formal d^8^-ML_4_ species, Ru(DAB-H)_2_ can exist as either a singlet or triplet ground state with a structure that can range from a square planar to a distorted tetrahedral geometry.^32,33^ Unrestricted optimization of Ru(DAB-H)_2_ confirmed the triplet species ^3^G to be 11.0 kcal mol^−1^ more stable (Δ*H*) than the corresponding singlet species ^1^G. The optimized structure of ^3^G (Fig. 3) exhibits a deformed square-planar structure that possesses idealized *D*_2_ symmetry due to a slight twist in the DAB rings. The origin of the observed twist is attributed to unfavorable van der Waals interactions between the phenyl groups on the adjacent chelate rings that effectively prohibit the core RuN_4_ atoms from adopting a planar arrangement. The N–Ru–N bite angle in each chelate ring is 77.8° while the mean intermolecular angle for the cis and trans N–Ru–N linkages between the rings is 103.9° and 166.0°, respectively. The related 16e complex Ru(dmpe)_2_ [where dmpe = 1,2-bis(dimethylphosphino)ethane] has been reported to be a singlet based on UV-vis data with a structure based on a square-planar geometry. The singlet state proposed for this d^8^-ML_4_ species was reproduced computationally using the model compound Ru(PH_3_)_4_.^33*c*^ That Ru(dmpe)_2_ is a singlet is reinforced by the near diffusion limit for the capture of CO and H_2_.^34^ The optimization of ^3^G as a triplet as opposed to a singlet in the case of Ru(dmpe)_2_ likely reflects subtle differences in the ordering of the frontier orbitals as influenced by the nature of the donor ligands (N *vs.* P) and the backbone of the chelating ligands.

**Fig. 4:**
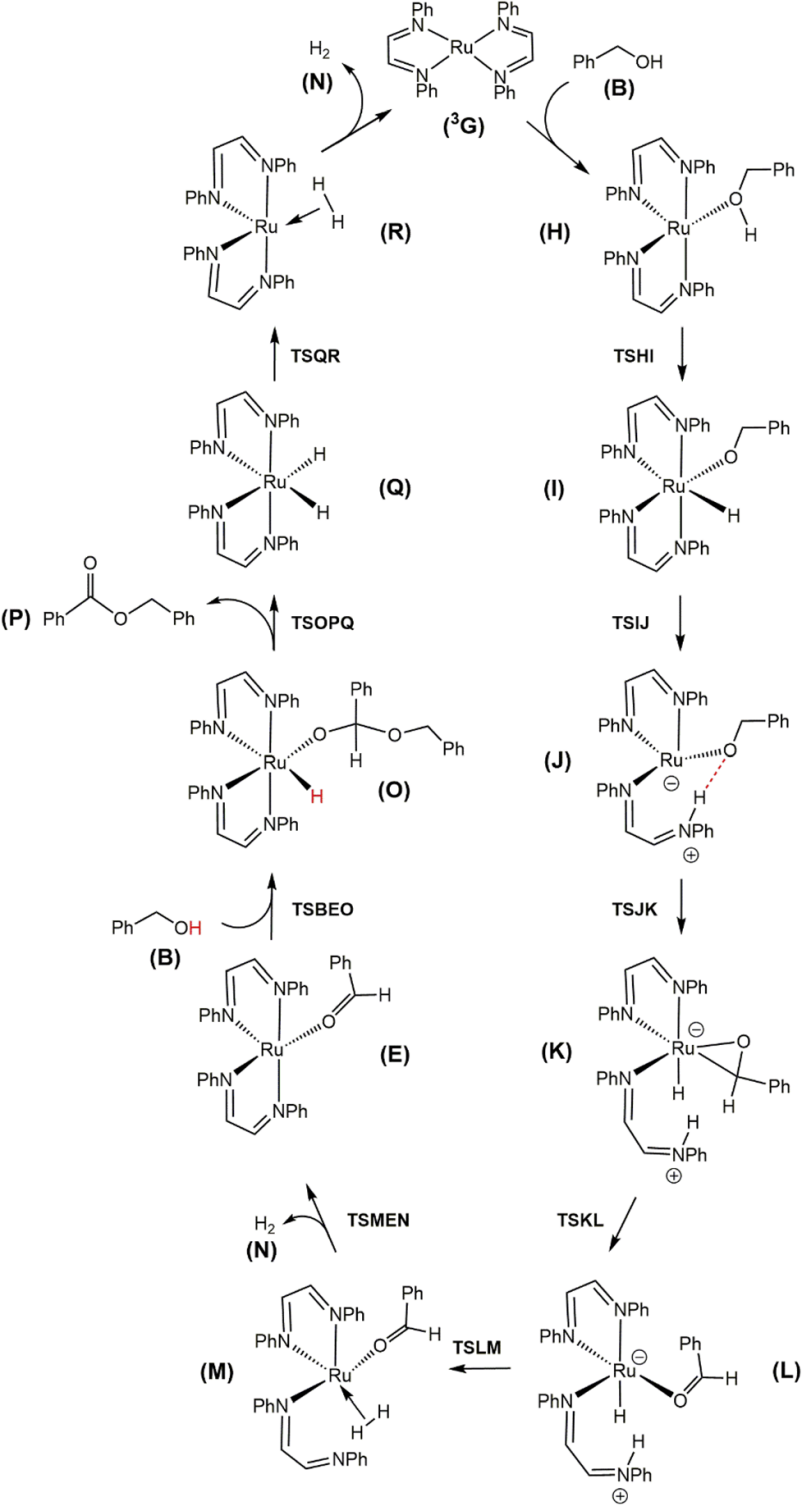
DFT-computed acceptorless dehydrogenative coupling mechanism from Ru(DAB-H)_2_ (^3^G) and benzyl alcohol (B) to give H_2_ (N) and benzyl benzoate (P).

**Fig. 5:**
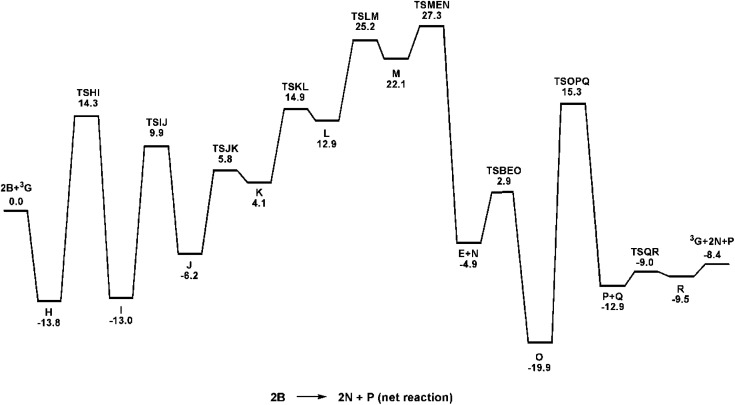
Potential energy profile for the reaction of Ru(DAB-H)_2_ (^3^G) and benzyl alcohol (B) to give H_2_ (N) and benzyl benzoate (P). Relative energies (Δ*H*) in kcal mol^−1^ with respect to 2B + ^3^G.

Fig. 4 shows the computed catalytic cycle starting from species ^3^G that is produced from species E on completion of the dehydrogenative C–O coupling with additional benzyl alcohol (B) or through dissociation of benzaldehyde (F) from species E. The unsaturated intermediate ^3^G is integral as an entry point into the catalytic cycle. The potential energy profile for the reaction under consideration is depicted in Fig. 5. Benzyl alcohol (B) addition to ^3^G affords the five-coordinate compound H which serves as the precursor to the octahedral hydridoalkoxide species I. Transfer of the hydride (as a proton) to an adjacent nitrogen atom of a DAB ligand proceeds *via*TSIJ and yields a protonated DAB ligand that undergoes a κ^2^-*N*,*N* → κ^1^-N transformation in the process to give species J that contains a κ^1^-*N*-C_6_H_5_N=C(H)(H)C=N(H)C_6_H_5_ ligand. The latter species, which lies 6.8 kcal mol^−1^ above I, is of interest because its formation underscores the chemically non-innocent role played by the ancillary DAB ligand in the catalytic cycle. Related to our results are the computational data reported by Li and Hall on the conversion of methanol to H_2_ and CO_2_ by the ruthenium complex [HRu(trop_2_dad)][K(dme)_2_] whose ancillary 1,4-bis(5*H*-dibenzo[*a*,*d*]cycloheptenyl-5-yl)-1,4-diazabuta-1,3-diene ligand (trop_2_dad) also functions as a chemically non-innocent ligand that exclusively controls the formation of the reported products.^35^ Here the ruthenium metal functions as a template for ligand binding and remains as a spectator throughout the catalytic cycle.

β-Hydrogen elimination from a benzylic site in J gives the hydrido species K that contains a κ^2^-*O*,*C*-benzaldehyde moiety and an intact and protonated κ^1^-*N*-DAB ligand. Transformation of the coordinated benzaldehyde moiety in K to the oxygen-bound κ^1^ species in L facilitates the formation of the molecular H_2_ complex M. The H_2_ ligand in M derives from a Noyori-type elimination involving the protonated “imine-like” N–H group on the κ^1^-*N*-DAB moiety and the terminal hydride ligand. The elimination process that gives M proceeds *via*TSLM with a barrier of 12.3 kcal mol^−1^. Dissociative loss of H_2_ from M is coupled with concomitant chelation of the pendant DAB ligand, leading to the aldehyde complex E which completes the first phase of the dehydrogenation reaction.

The C–O bond coupling portion of the ADC reaction is initiated by TSBEO. The second equivalent of benzyl alcohol (B) adds to the vacant site in E that is syn to the coordinated benzaldehyde to furnish the hydridoalkoxide O. The H–O moiety of the incoming alcohol adds synchronously to the metal center and aldehyde carbon in E, respectively, to form a five-membered ring not unlike that reported for other substrate activation processes that display bifunctional behavior.^14*g*,36^ This addition step exhibits a barrier of 7.8 kcal mol^−1^ and proceeds through TSBEO. Transfer of the hydride (as a proton) in O to an adjacent nitrogen atom was modeled as was done for I → J, but all attempts to model this process were unfavorable, giving a κ^2^-*O*,*O*-coordinated alkoxyether species and other unreasonable structures that proved resistant to β-hydrogen elimination and release of the product ester. We were able to successfully model the release of the benzyl benzoate product (P) through a benzylic β-hydrogen elimination through TSOPQ that is overall rate-limiting and proceeds with a barrier of 35.2 kcal mol^−1^. Activation of the C–H bond in this step is accompanied by the release of the ester P and formation of the dihydride Q. The penultimate step involves the reductive elimination of the hydrides to give the H_2_ complex R, which on the release of H_2_ (N) furnishes ^3^G to complete the ADC reaction. Finally, the overall reaction (eqn (1)) is computed to be exothermic by 8.4 kcal mol^−1^. It is relevant to mention here that utilization of imine-coordinated ruthenium(0) species in catalysis is precedent in the literature.^29*a*,37^

## Conclusion

We have prepared a series of ruthenium compounds with the general formula *cis*-Ru(DAB-X)_2_Cl_2_ [where DAB = *p*-XC_6_H_4_N=C(H)(H)C=NC_6_H_4_X-*p*; X= H, CH_3_, OCH_3_, and Cl] and have investigated their reactivity in the dehydrogenation of primary and secondary alcohols. We confirm that the *cis*-Ru(DAB-X)_2_Cl_2_ complexes are efficient catalyst precursors for acceptorless dehydrogenation of primary alcohols, which is followed by dehydrogenative C–O coupling of the resulting aldehyde and the additional alcohol to yield esters and H_2_. The dehydrogenation reaction with secondary alcohols proceeds similarly and stops at the ketone product. DFT calculations support the coordination of the alcohol substrate by the unsaturated species Ru(κ^2^-*N*,*N*-DAB)_2_ to give the κ^1^-*O* alcohol compound H. Transfer of the hydroxy hydrogen in H to the metal affords the octahedral intermediate *cis*-HRu(κ^2^-*N*,*N*-DAB)_2_(κ^1^-OCH_2_Ph) (I), whose hydride is transferred to an adjacent DAB ligand to give the protonated ligand complex Ru[κ^2^-*N*,*N*-C_6_H_5_N=C(H)(H)C=NC_6_H_5_][κ^1^-*N*-C_6_H_5_N=C(H)(H)C=N(H)C_6_H_5_](κ^1^-OCH_2_Ph) (J). The ability of the chemically non-innocent DAB ligand to function as a proton acceptor, coupled with its participation in the bifunctional release of H_2_ from the hydride species HRu[κ^2^-*N*,*N*-C_6_H_5_N=C(H)(H)C=NC_6_H_5_][κ^1^-*N*-C_6_H_5_N=C(H)(H)C=N(H)C_6_H_5_](κ^1^-*O*-benzaldehyde) (L) through an outer-sphere elimination is computationally supported. Our current efforts center on the study of other nitrogen-based donors that can function in a capacity similar to that exhibited by the DAB ligands here. The catalytic results from these studies will be disseminated in due course.

## Experimental section

### Materials

Ruthenium trichloride was purchased from Arora Matthey, Kolkata, India. *cis*,*fac*-Ru(dmso)_4_Cl_2_ was prepared by following a reported procedure.^38^ Glyoxal was obtained from SD Fine Chem, Mumbai, India, and the 4-X-anilines (X = OCH_3_, CH_3_, H, and Cl) were procured from Merck, India. The 1,4-diazabutadiene ligands were prepared by condensation of glyoxal with the 4-X-anilines in hot methanol. All other chemicals and solvents were reagent grade commercial materials and were used as received.

### Physical measurements

Microanalyses (C, H, and N) were performed using a Heraeus Carlo Erba 1108 elemental analyzer, and magnetic susceptibilities were measured using a Sherwood MK-1 balance. ^1^H NMR spectra were recorded in CDCl_3_ solution at 300 MHz on a Bruker Avance DPX 300 NMR spectrometer. IR spectra were recorded on a PerkinElmer Spectrum Two IR spectrometer, with samples prepared as KBr pellets. Electronic spectra were recorded on a JASCO V-630 spectrophotometer. Solution electrical conductivities were measured using an Elico CM 180 conductivity meter with a solute concentration of *ca.* 10^−3^ M. GC-MS analyses were performed using a PerkinElmer CLARUS 680 instrument.

### Compound preparation

The synthetic details associated with the preparation of the isomeric compounds *trans*-Ru[κ^2^-*N*,*N-p*-XC_6_H_4_N=C(H)(H)C=NC_6_H_4_X-*p*]_2_Cl_2_ (*trans*-1) and *cis*-Ru[κ^2^-*N*,*N-p*-XC_6_H_4_N=C(H)(H)C=NC_6_H_4_X-*p*]_2_Cl_2_ (*cis*-1) are described below. The remaining six compounds were synthesized similarly and we report only the yield and physical data for each derivative of 2–4.

#### *trans*-1

To a solution of DAB-H (0.11 g, 0.51 mmol) in warm acetone (40 mL) was added *cis*-Ru(dmso)_4_Cl_2_ (0.10 g, 0.21 mmol), and the mixture was heated at reflux for 8 h. The resulting dark solution was allowed to stand overnight, whereby *trans*-1 separated as a dark crystalline precipitate, which was collected by filtration, washed with cold acetone, and dried in air. Additional *trans*-1 was obtained from the filtrate following evaporation and chromatographic purification of the residue on a silica plate using 1 : 1 dichloromethane–chloroform as the eluent. Yield: 0.10 g (80%). Anal. calc. for C_28_H_24_N_4_Cl_2_Ru: C, 57.13; H, 4.08; N, 9.52. Found: C, 57.18; H, 4.11; N, 9.49. ^1^H NMR (300 MHz, CDCl_3_): *δ* 6.90 (t, 8H, *J* = 7.5); 7.08 (t, 4H, *J* = 7.5); 7.22 (d, 8H, *J* = 7.5); 8.68 (s, 4H). IR (KBr pellet, cm^−1^): 521, 585, 604, 623, 696, 759, 836, 852, 881, 916, 1002, 1028, 1071, 1175, 1208, 1300, 1312, 1342, 1447, 1483, 1584, 1628. UV-vis in CH_2_Cl_2_ [*λ*_max_/nm (*ε*/M^−1^ cm^−1^)]: 706 (8700), 411 (7900), 307 (8100), 269 (11 800).

#### *cis*-1

To a solution of DAB-H (0.11 g, 0.51 mmol) in *o*-xylene (40 mL) was added *cis*-Ru(dmso)_4_Cl_2_ (0.10 g, 0.21 mmol), after which the mixture was heated at reflux for 10 h. The resulting bluish-green solution was allowed to stand overnight, whereby *cis*-1 separated as a dark crystalline solid. The desired product was collected by filtration, washed with ether, and dried in air. Additional *cis*-1 was obtained from the filtrate following evaporation and chromatographic purification of the residue on a silica plate using 1 : 1 dichloromethane–chloroform as the eluent. Yield: 0.09 g (77%). Anal. calc. for C_28_H_24_N_4_Cl_2_Ru: C, 57.13; H, 4.08; N, 9.52. Found: C, 57.18; H, 4.11; N, 9.49. ^1^H NMR (300 MHz, CDCl_3_): *δ* 6.78 (d, 4H, *J* = 7.5); 7.01–7.32 (m, 12H)*; 7.37 (d, 4H, *J* = 7.2); 8.32 (s, 2H); 8.50 (s, 2H). IR (KBr pellet, cm^−1^): 524, 613, 694, 759, 846, 954, 1024, 1075, 1208, 1275, 1345, 1447, 1485, 1536, 1591, 1625. UV-vis in CH_2_Cl_2_ [*λ*_max_/nm (*ε*/M^−1^ cm^−1^)]: 572 (7500), 473 (2300), 350 (10 000).

#### *trans*-2

Yield: 0.11 g (81%). Anal. calc. for C_32_H_32_N_4_Cl_2_Ru: C, 59.63; H, 4.97; N, 8.70. Found: C, 59.91; H, 4.95; N, 8.73. ^1^H NMR (300 MHz, CDCl_3_): *δ* 2.27 (s, 12H, CH_3_); 6.69 (d, 8H, *J* = 8.1); 7.09 (d, 8H, *J* = 8.1); 8.64 (s, 4H). IR (KBr pellet, cm^−1^): 502, 559, 645, 763, 807, 847, 867, 1020, 1039, 1062, 1103, 1170, 1210, 1278, 1347, 1380, 1466, 1499, 1634. UV-vis in CH_2_Cl_2_ [*λ*_max_/nm (*ε*/M^−1^ cm^−1^)]: 707 (8000), 434 (9400), 313 (7800), 267 (12 000).

#### *cis*-2

Yield: 0.10 g (78%). Anal. calc. for C_32_H_32_N_4_Cl_2_Ru: C, 59.63; H, 4.97; N, 8.70. Found: C, 60.28; H, 4.98; N, 8.79. ^1^H NMR (300 MHz, CDCl_3_): *δ* 2.33 (s, 6H, CH_3_); 2.40 (s, 6H, CH_3_); 6.68 (d, 4H, *J* = 8.5); 6.79 (d, 4H, *J* = 8.5); 7.04 (d, 4H, *J* = 8.5); 7.13 (d, 4H, *J* = 8.5); 8.25 (s, 2H); 8.41 (s, 2H). IR (KBr pellet, cm^−1^): 569, 770, 815, 956, 1021, 1069, 1108, 1215, 1345, 1361, 1454, 1501, 1623. UV-vis in CH_2_Cl_2_ [*λ*_max_/nm (*ε*/M^−1^ cm^−1^)]: 576 (8300), 479 (2900), 372 (14 000).

#### *trans*-3

Yield: 0.13 g (91%). Anal. calc. for C_32_H_32_N_4_O_4_Cl_2_Ru: C, 54.24; H, 4.52; N, 7.91. Found: C, 54.33; H, 4.53; N, 7.93. ^1^H NMR (300 MHz, CDCl_3_): *δ* 3.78 (s, 12H, OCH_3_), 6.43 (d, 8H, *J* = 8.9), 7.18 (d, 8H, *J* = 8.9), 8.67 (s, 4H). IR (KBr pellet, cm^−1^): 461, 505, 556, 573, 642, 645, 661, 760, 798, 828, 846, 867, 925, 938, 1034, 1069, 1113, 1169, 1212, 1254, 1298, 1349, 1456, 1502, 1550, 1601. UV-vis in CH_2_Cl_2_ [*λ*_max_/nm (*ε*/M^−1^ cm^−1^)]: 708 (10 600), 472 (15 500), 343 (10 000), 238 (35 900).

#### *cis*-3

Yield: 0.12 g (82%). Anal. calc. for C_32_H_32_N_4_O_4_Cl_2_Ru: C, 54.24; H, 4.52; N, 7.91. Found: C, 54.72; H, 4.49; N, 7.96. ^1^H NMR (300 MHz, CDCl_3_): *δ* 3.83 (s, 6H, OCH_3_); 3.84 (s, 6H, OCH_3_); 6.76 (d, 4H, *J* = 8.7); 6.80 (d, 4H, *J* = 8.7); 7.11 (d, 4H, *J* = 8.8); 7.36 (d, 4H, *J* = 8.8); 8.19 (s, 2H); 8.34 (s, 2H). IR (KBr pellet, cm^−1^): 569, 658, 756, 798, 824, 1031, 1107, 1168, 1212, 1254, 1301, 1342, 1502, 1580, 1602. UV-vis in CH_2_Cl_2_ [*λ*_max_/nm (*ε*/M^−1^ cm^−1^)]: 586 (7800), 407 (17 400), 241 (33 200).

#### *trans*-4

Yield: 0.11 g (75%). Anal. calc. for C_28_H_20_N_4_Cl_6_Ru: C, 46.27; H, 2.75; N, 7.71. Found: C, 46.44; H, 2.73; N, 7.71. ^1^H NMR (300 MHz, CDCl_3_): *δ* 6.98 (d, 8H, *J* = 8.6); 7.21 (d, 8H, *J* = 8.6); 8.65 (s, 4H). IR (KBr pellet, cm^−1^): 535, 677, 715, 752, 819, 833, 874, 1014, 1091, 1167, 1208, 1345, 1412, 1456, 1482, 1590, 1635. UV-vis in CH_2_Cl_2_ [*λ*_max_/nm (*ε*/M^−1^ cm^−1^)]: 714 (6300), 421 (7300), 309 (7600), 281 (10 000).

#### *cis*-4

Yield: 0.11 g (75%). Anal. calc. for C_28_H_20_N_4_Cl_6_Ru: C, 46.27; H, 2.75; N, 7.71. Found: C, 46.52; H, 2.79; N, 7.68. ^1^H NMR (300 MHz, CDCl_3_): *δ* 6.43 (d, 4H, *J* = 8.6); 6.72 (d, 4H, *J* = 8.6); 7.10 (d, 4H, *J* = 8.5); 7.18 (d, 4H, *J* = 8.5); 8.33 (s, 2H); 8.50 (s, 2H). IR (KBr pellet, cm^−1^): 540, 597, 683, 715, 750, 823, 1013, 1091, 1170, 1211, 1285, 1345, 1405, 1450, 1480, 1539, 1588, 1635. UV-vis in CH_2_Cl_2_ [*λ*_max_/nm (*ε*/M^−1^ cm^−1^)]: 578 (10 400), 479 (3100), 364 (17 100).

### X-ray crystallography

Methods for growing single crystals of the complexes: *trans*-3 and *trans*-1: slow evaporation of solvents from solutions of the complexes in 1 : 1 acetonitrile–dichloromethane; *trans*-2: slow diffusion of toluene into a solution of the complex in dichloromethane; *trans*-4: slow evaporation of solvents from a solution of the complex in 1 : 1 acetonitrile–chloroform; *cis*-3, *cis*-2 and *cis*-1: diffusion of toluene (for *cis*-3 and *cis*-1) or benzene (for *cis*-2) into a solution of the complex in dichloromethane (for *cis*-3 and *cis*-2), or chloroform (for *cis*-1); *cis*-4: slow evaporation of solvent from a solution of the complex in toluene.

The molecular structure of each compound was established by X-ray crystallography. Tables S1–S4 (ESI†) contain the pertinent crystallographic data collection and processing parameters for the four pairs of isomers. The X-ray data for *trans*-3 were collected on a CrysAlis Pro, Super Nova (Agilent Technologies) diffractometer using Cu Kα radiation (*λ* = 1.54184 Å), while the data on the remaining compounds were collected on a Bruker SMART CCD diffractometer using Mo Kα radiation (*λ* = 0.71073 Å). X-ray data reduction, structure solution, and refinement were done using the SHELXS-97 and SHELXL-97 packages, and Olex2.^39^ The structures were solved by direct methods. Tables S5–S8 (ESI†) report selected bond distances and angles for the isomeric pairs of compounds.

### General procedure for acceptorless dehydrogenation of alcohols

In a typical run, an oven-dried 10 mL round-bottomed flask was charged with the alcohol substrate (1.0 mmol), KO^*t*^Bu (1.0 mol%), and the desired catalyst precursor (0.1–0.2 mol%), followed the addition of 5 mL of a binary solvent mixture composed of 1 : 4 dichloromethane–toluene. The flask was attached to a condenser fitted with a rubber septum at the end. A needle was pierced through the septum to allow release of uncondensed volatiles. The whole setup was placed in a preheated oil bath for refluxing. After the specified time, the flask was removed from the oil bath and water (20 mL) added, followed by extraction with diethyl ether (4 × 10 mL). The combined organic layers were washed with water (3 × 10 mL), dried over anhydrous Na_2_SO_4_, and filtered. The solvent was removed under vacuum, after which the residue was dissolved in hexane and analyzed by GCMS.

For verification of H_2_ evolution, the open end of the needle was sealed with a rubber block. After about 30 min, the rubber block was removed and the tip of the needle was inserted into ice-cold CDCl_3_, kept in a NMR tube, for 1 min. The resulting solution was examined for presence of H_2_ by ^1^H NMR spectroscopy.

### Computational modeling details

The reported DFT calculations were performed with the hybrid meta exchange–correlation functional M06,^40^ as implemented by the Gaussian 09 program package.^41^ The Ru atoms were described by Stuttgart–Dresden effective core potentials (ECP28MWB) and an SDD basis set,^42^ while a 6-31G(d′) basis set was employed for the remaining atoms.^43^

The reported geometries (gas-phase at 298.15 K) represent fully optimized ground states (positive eigenvalues) and transition states (one imaginary eigenvalue) as verified from the analytical Hessian. The computed frequencies were used to make zero-point and thermal corrections to the electronic energies; the reported enthalpies (Δ*H*) are quoted in kcal mol^−1^ relative to the specified standard. The geometry-optimized structures have been drawn with the JIMP2 molecular visualization and manipulation program.^44^

Energy differences between *cis* and *trans* geometries of complexes 1–4 were done using density functional theory (DFT) with the B3LYP exchange–correlation functional,^45^ as implemented in Gaussian 09 program.^41^ The lanl2dz basis set was used for Ru,^46^ and 6-31G(d) was employed for the other elements.^47^

## Conflicts of interest

The authors declare no conflict of interest.

## Supplementary Material

RA-013-D3RA04750D-s001

RA-013-D3RA04750D-s002
