# Supplementary material for: Ruthenium complexes of 1,4-diazabutadiene ligands with a cis-RuCl2 moiety for catalytic acceptorless dehydrogenation of alcohols: DFT evidence of chemically non-innocent ligand participation
Source: RSC Adv. 2023 Aug 29;13(36):25660–72. doi: 10.1039/d3ra04750d (PMC10463240; doi:10.1039/d3ra04750d)
Supplement: RA-013-D3RA04750D-s001 [file RA-013-D3RA04750D-s001.pdf]

Electronic Supplementary Material (ESI) for RSC Advances.
This journal is © The Royal Society of Chemistry 2023

Electronic supplementary information (ESI)

Ruthenium complexes of 1,4-diazabutadiene ligands with a *cis*-$RuCl_2$ moiety for catalytic acceptorless dehydrogenation of alcohols: DFT evidence for chemically non-innocent ligand participation

Aparajita Mukherjee,[a] Sayanti Datta,[b] Michael G. Richmond*[c] and Samaresh Bhattacharya*[a]

[a] *Department of Chemistry, Inorganic Chemistry Section, Jadavpur University, Kolkata 700 032, India. E-mail: samaresh_b@yahoo.com*

[b] *Department of Chemistry, Brainware University, Kolkata 700 125, India.*

[c] *Department of Chemistry, University of North Texas, Denton, TX 76203, USA*

Contents:

Table S1	Crystallographic data for *trans*-1 and *cis*-1	Pg 3
Table S2	Crystallographic data for *trans*-2 and *cis*-2	Pg 4
Table S3	Crystallographic data for *trans*-3 and *cis*-3	Pg 5
Table S3	Crystallographic data for *trans*-3 and *cis*-3	Pg 6
Fig. S1	Molecular structures of *trans*-1, *trans*-2 and *trans*-4	Pg 7
Fig. S2	Energy difference between the *trans* and *cis* isomers of complex **1-4**.	Pg 8-11
Fig. S3	Molecular structures of *cis*-1, *cis*-2 and *cis*-4	Pg 12
Table S5	Selected bond lengths (Å) and bond angles (deg) for trans-1 and cis-1	Pg 13
Table S6	Selected bond lengths (Å) and bond angles (deg) for *trans*-2 and *cis*-2	Pg 14
Table S7	Selected bond lengths (Å) and bond angles (deg) for *trans*-3 and *cis*-3	Pg 15
Table S8	Selected bond lengths (Å) and bond angles (deg) for *trans*-4 and *cis*-4	Pg 16
Table S9	Cyclic voltammetric data	Pg 17
Fig. S4	Cyclic voltammogram of *cis*-Ru(DAB-OCH_3)$_2Cl_2$ in acetonitrile solution (0.1 M TBHP) at a scan rate of 50 mVs^{-1}.	Pg 18
Fig. S5	Least-squares plot of $E_{½}$ values of Ru(II)-Ru(III) couple versus Hammett substituent constant (4σ) for the *trans*-isomers and the *cis*-isomers.	Pg 19
Table S10	Initial catalytic reactions on benzyl alcohol dehydrogenation and optimization of experimental parameters.	Pg 20
Fig. S6	^1H NMR of H_2 (4.616 ppm) evolved from primary alcohol (benzyl alcohol) oxidation.	Pg 21
Fig. S7	^1H NMR of H_2 (at 4.621 ppm) evolved from secondary alcohol (cyclohexanol) oxidation.	Pg 22
Table S10	DFT data	Pg 23-68

Table S1 Crystallographic data for *trans*-**1** and *cis*-**1**

	trans-**1**	*cis*-**1**
empirical formula	$2(C_{28}H_{24}Cl_2N_4Ru),O$	$C_{28}H_{24}N_4Cl_2Ru{\cdot}2CHCl_3$
formula weight	1192.96	827.22
crystal system	monoclinic	triclinic
space group	$P2_1/c$	$P\bar{1}$
a (Å)	9.8151(7)	10.8657(2)
b (Å)	12.8431(10)	13.3710(2)
c (Å)	19.5758(14)	14.4857(3)
α (◦)	90	112.698(1)
β (◦)	91.003(5)	94.014(1)
γ (◦)	90	113.094(1)
V (Å^3)	2467.3(3)	1724.15(6)
Z	2	2
F (000)	1208	828
crystal size (mm)	0.08 × 0.12 × 0.24	0.10 × 0.15 × 0.24
T (K)	296	298
μ (mm^{-1})	0.880	1.102
R1[a]	0.1179	0.0448
wR2[b]	0.3769	0.1102
GOF[c]	1.069	1.05

[a] $R1 = \Sigma||F_o|-|F_c||/\Sigma|F_o|$.
[b] $wR2 = [\Sigma\{w(F_o^2-F_c^2)^2\}/\Sigma\{w(F_o^2)\}]^{1/2}$.
[c] $GOF = [\Sigma(w(F_o^2-F_c^2)^2)/(M-N)]^{1/2}$, where M is the number of reflections and N is the number of parameters refined.

Table S2 Crystallographic data for *trans*-**2** and *cis*-**2**

	trans-**2**	*cis*-**2**
empirical formula	$C_{32}H_{32}Cl_2N_4Ru$	$C_{32}H_{32}N_4Cl_2Ru$ [+ solvent]
formula weight	644.59	667.66
crystal system	monoclinic	monoclinic
space group	$P2_1/c$	$P2_1/c$
a (Å)	13.4650(4)	14.0932(10)
b (Å)	12.0716(3)	14.4484(10)
c (Å)	9.4185(3)	17.7270(14)
β (◦)	103.729(2)	104.930(6)
V ($Å^3$)	1487.18(8)	3487.8(5)
Z	2	4
F (000)	660	1392
crystal size (mm)	0.14 × 0.11 × 0.10	0.14 × 0.15 × 0.20
T (K)	296	273
μ (mm^{-1})	0.735	0.631
R1[a]	0.0349	0.0647
wR2[b]	0.0910	0.1829
GOF[c]	1.03	0.938

[a] $R1 = \Sigma||F_o|-|F_c||/\Sigma|F_o|$.
[b] $wR2 = [\Sigma\{w(F_o^2-F_c^2)^2\}/\Sigma\{w(F_o^2)\}]^{1/2}$.
[c] $GOF = [\Sigma(w(F_o^2-F_c^2)^2)/(M-N)]^{1/2}$, where M is the number of reflections and N is the number of parameters refined.

Table S3 Crystallographic data for *trans*-**3** and *cis*-**3**

	trans-**3**	*cis*-**3**
formula	$C_{32}H_{32}N_4O_4Cl_2Ru$	$C_{32}H_{29}N_4O_4Cl_2Ru$ [+ solvent]
fw	708.59	708.60
crystal system	monoclinic	triclinic
space group	$P2_1/c$	$P\bar{1}$
a (Å)	14.4288(6)	10.4958(4)
b (Å)	11.9755(4)	13.5516(5)
c (Å)	8.8550(4)	13.8771(5)
α (°)	90	108.633(2)
β (°)	105.424(4)	92.763(2)
γ (°)	90	94.144(3)
V (Å^3)	1474.97(11)	1860.14(12)
Z	2	2
F (000)	724	722
crystal size (mm)	0.03 × 0.09 × 0.12	0.12 × 0.14 × 0.18
T (K)	100	273
μ (mm^{-1})	6.344	0.601
R1[a]	0.0333	0.0660
wR2[b]	0.0878	0.2153
GOF[c]	1.04	1.027

[a] $R1 = \Sigma||F_o| - |F_c|| / \Sigma|F_o|$.
[b] $wR2 = [\Sigma\{w(F_o^2-F_c^2)^2\}/\Sigma\{w(F_o^2)\}]^{1/2}$.
[c] $GOF = [\Sigma(w(F_o^2-F_c^2)^2)/(M-N)]^{1/2}$, where M is the number of reflections and N is the number of parameters refined

Table S4 Crystallographic data for *trans*-**4** and *cis*-**4**

	trans-**4**	*cis*-**4**
empirical formula	$C_{28}H_{20}N_4Cl_6Ru$	$C_{28}H_{20}N_4Cl_6Ru$
formula weight	726.25	726.25
crystal system	triclinic	monoclinic
space group	$P\overline{1}$	C2/c
a (Å)	7.2857(9)	28.7859(12)
b (Å)	13.9992(17)	15.6535(6)
c (Å)	14.5426(18)	14.3195(5)
α (°)	78.661(8)	90
β (°)	89.453(8)	113.084(2)
γ (°)	84.435(8)	90
V (Å^3)	1447.4(3)	5935.7(4)
Z	2	8
F (000)	724	2872
crystal size (mm)	0.10 × 0.10 × 0.20	0.11 × 0.16 × 0.28
T (K)	296	296
μ (mm^{-1})	1.122	1.094
R1[a]	0.1303	0.0483
wR2[b]	0.4469	0.1536
GOF[c]	1.35	0.89

[a] $R1 = \Sigma||F_o| - |F_c|| / \Sigma|F_o|$.
[b] $wR2 = [\Sigma\{w(F_o^2-F_c^2)^2\}/\Sigma\{w(F_o^2)\}]^{1/2}$.
[c] $GOF = [\Sigma(w(F_o^2-F_c^2)^2)/(M-N)]^{1/2}$, where M is the number of reflections and N is the number of parameters refined.

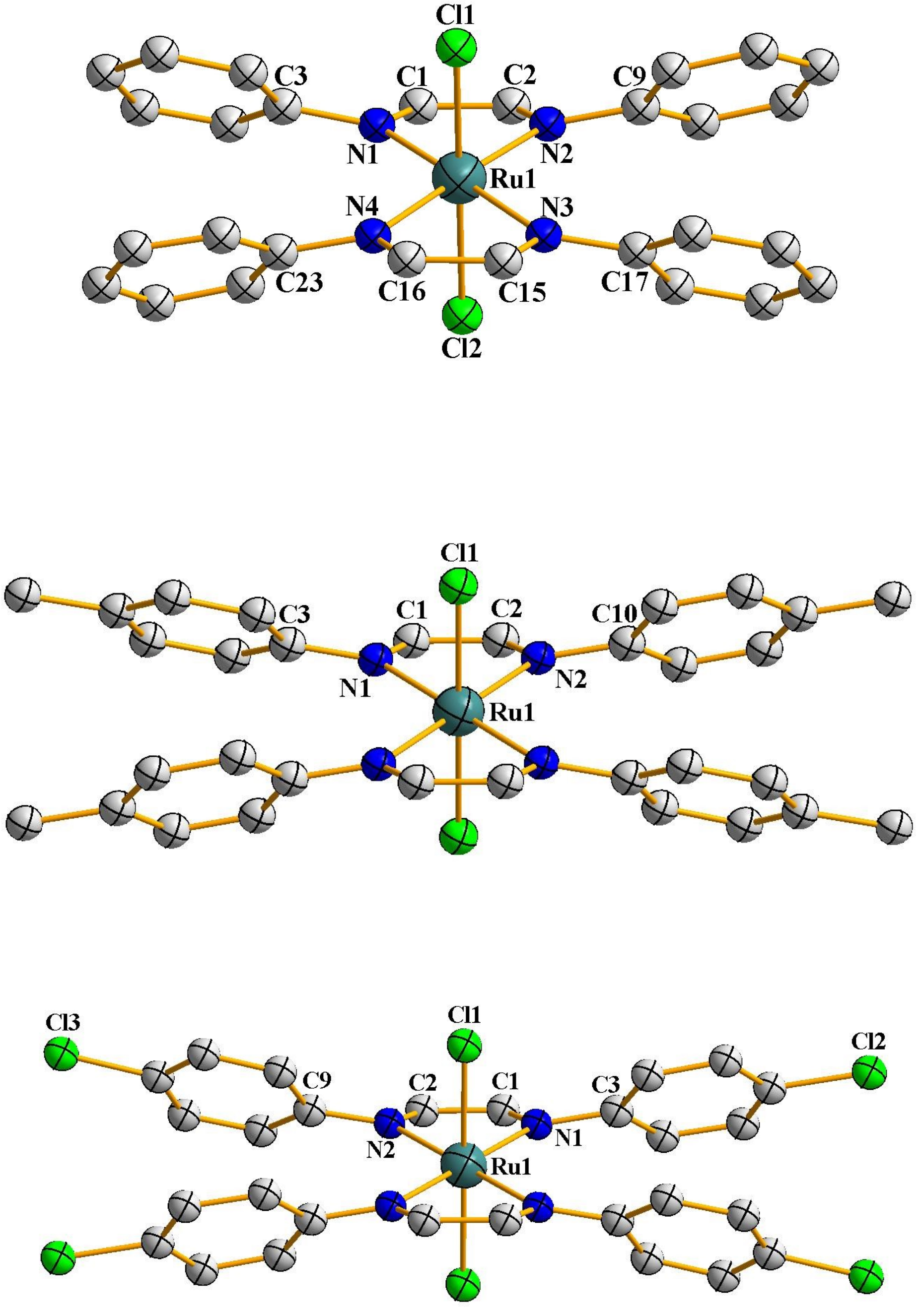

Fig. S1 Molecular structures of *trans*-**1** (top), *trans*-**2** (middle), and *trans*-**4** (bottom).

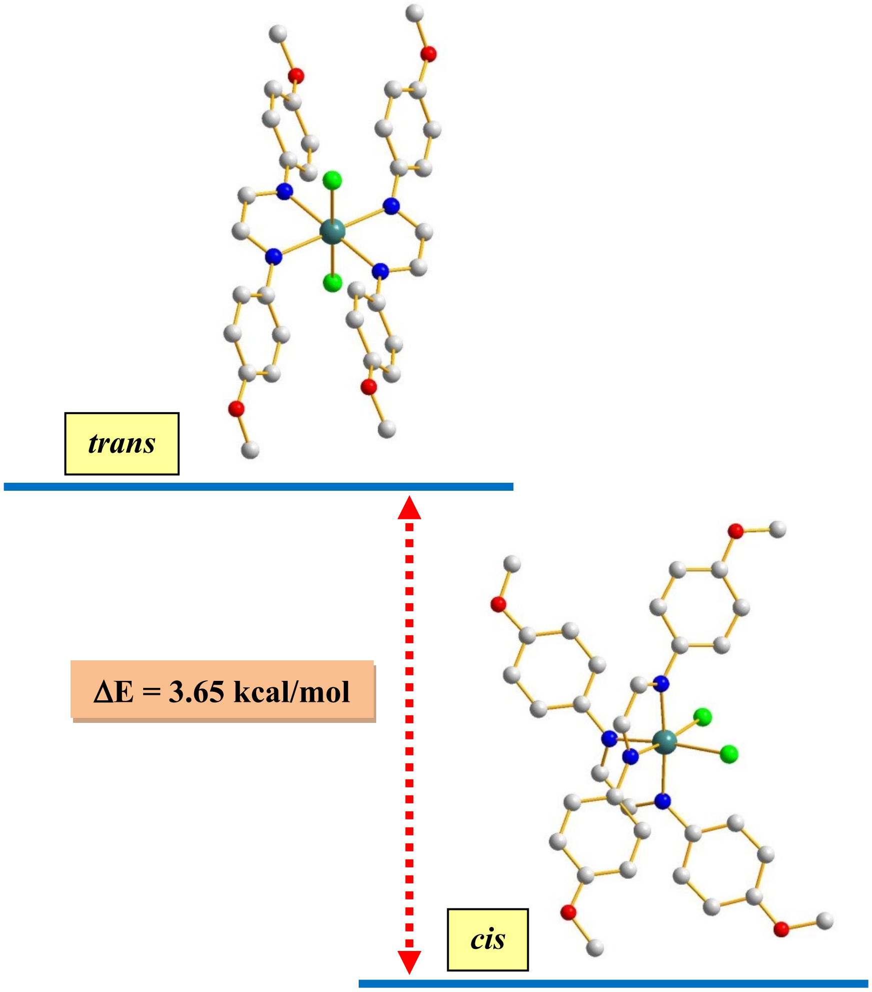

Fig. S2a Energy difference between the *trans* and *cis* isomers of **3**.

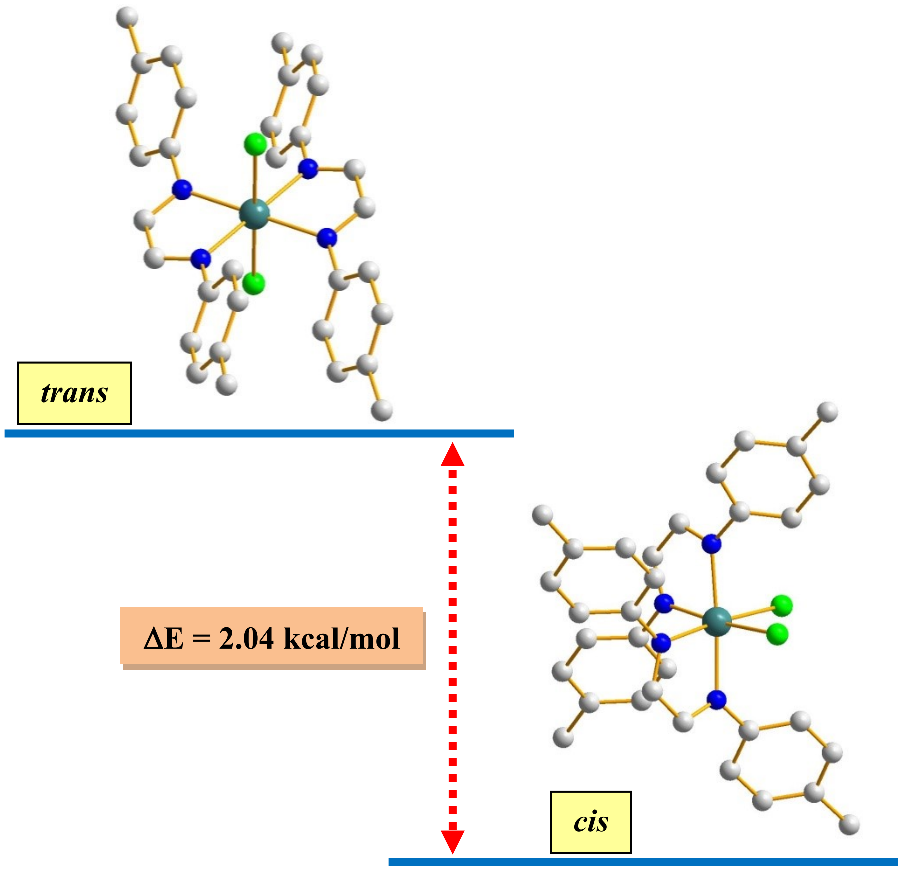

Fig. S2b Energy difference between the *trans* and *cis* isomers of **2**.

.

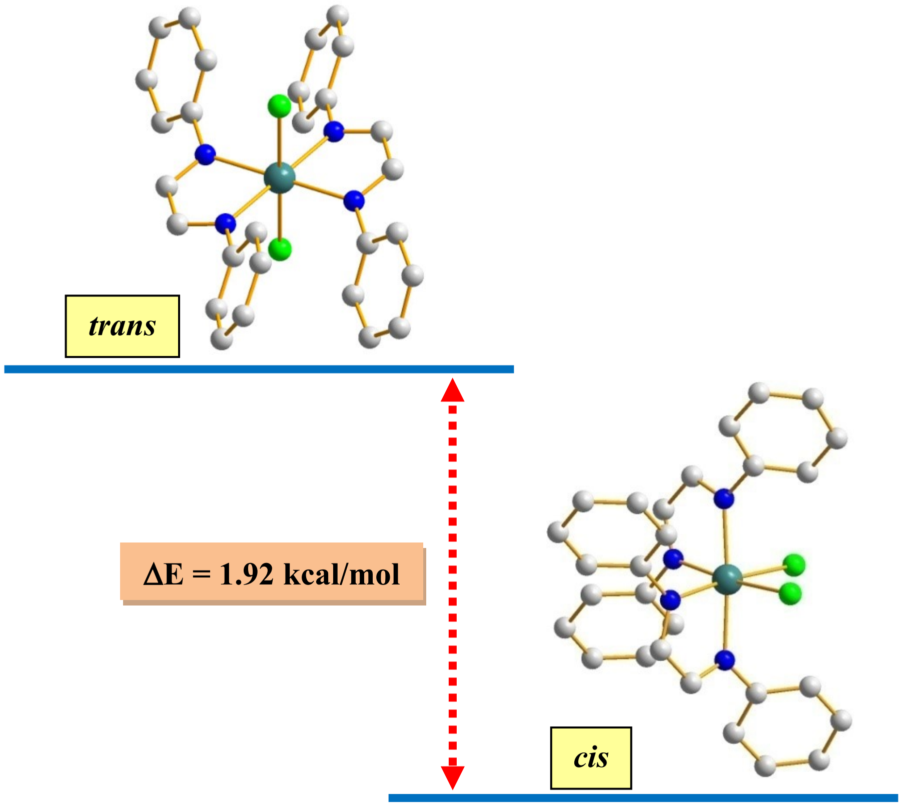

Fig. S2c Energy difference between the *trans* and *cis* isomers of **1**.

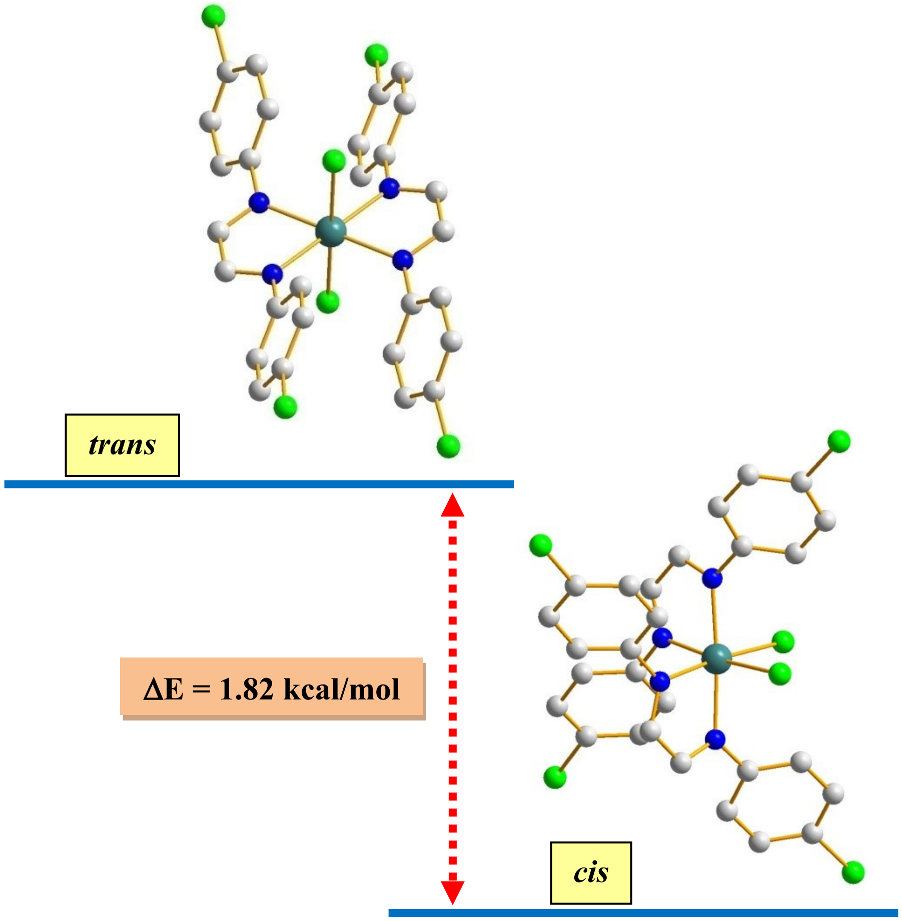

Fig. S2d Energy difference between the *trans* and *cis* isomers of **4**.

.

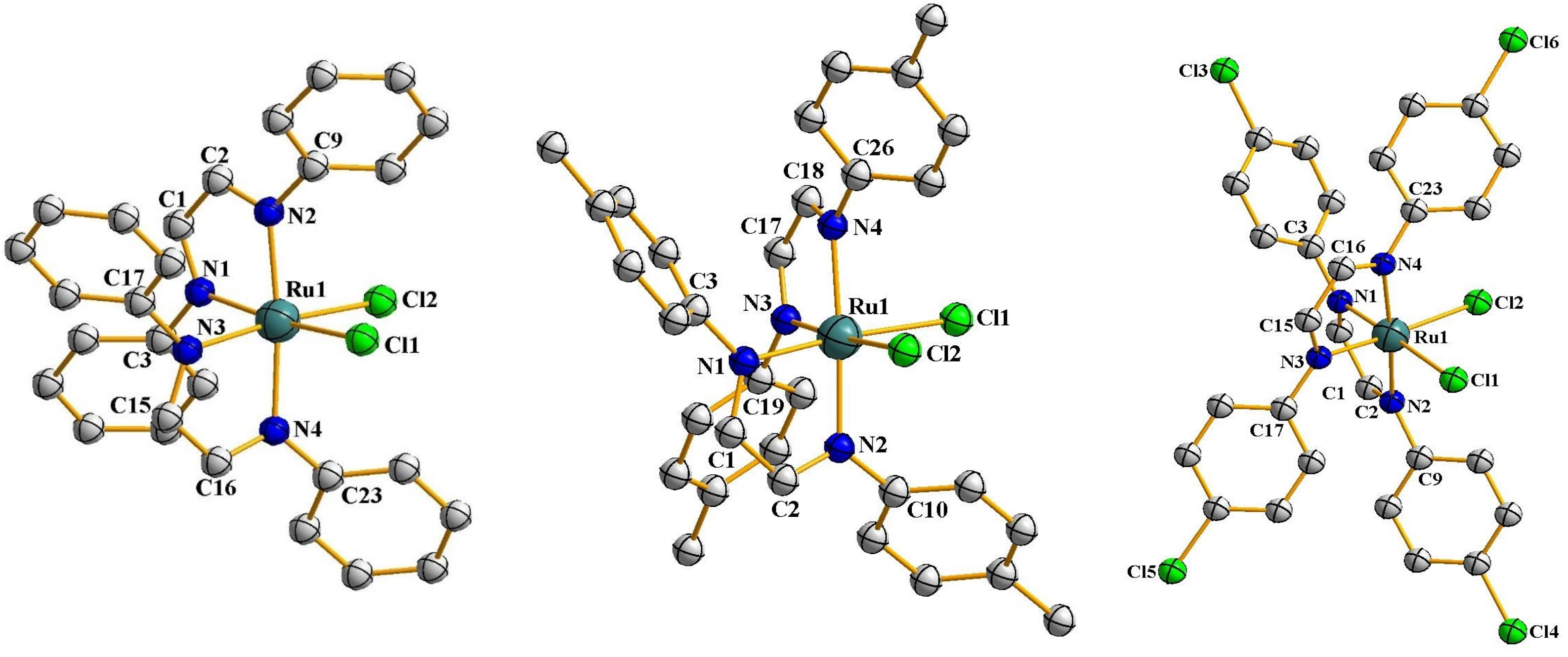

Fig. S3 Molecular structures of *cis*-**1** (left), *cis*-**2** (middle), and *cis*-**4** (right).

Table S5 Selected bond lengths (Å) and bond angles (deg) for *trans*-**1** and *cis*-**1**

trans-**1**		*cis-1*	
Bond lengths (Å)			
Ru1-Cl1	2.392(3)	Ru1-Cl1	2.4009(10)
Ru1-Cl2	2.386(3)	Ru1-Cl2	2.4056(10)
Ru1-N1	2.032(10)	Ru1-N1	2.046(3)
Ru1-N2	2.054(10)	Ru1-N2	2.052(3)
Ru1-N3	2.105(10)	Ru1-N3	2.027(3)
Ru1-N4	2.081(10)	Ru1-N4	2.054(3)
C1-N1	1.319(16)	C1-N1	1.295(5)
C2-N2	1.309(17)	C2-N2	1.294(6)
C1-C2	1.40(2)	C1-C2	1.421(6)
C3-N1	1.455(16)	C3-N1	1.433(5)
C9-N2	1.438(15)	C9-N2	1.449(5)
Bond angles (deg)			
Cl1-Ru1-Cl2	174.70(14)	N1-Ru1-Cl1	171.29(8)
N1-Ru1-N3	178.5(4)	N3-Ru1-Cl2	171.39(8)
N2-Ru1-N4	177.8(4)	N2-Ru1-N4	173.83(13)
		Cl1-Ru1-Cl2	92.29(3)
N1-Ru1-N2	78.4(4)	N1-Ru1-N2	77.45(12)
Ru1-N1-C1	112.2(8)	Ru1-N1-C1	114.7(3)
N1-C1-C2	117.1(11)	N1-C1-C2	116.6(4)
C1-C2-N2	116.3(12)	C1-C2-N2	115.8(4)
C2-N2-Ru1	112.2(9)	C2-N2-Ru1	115.1(3)
N3-Ru1-N4	76.3(4)	N3-Ru1-N4	77.60(11)
Ru1-N3-C15	113.4(9)	Ru1-N3-C15	115.5(2)
N3-C15-C16	116.1(12)	N3-C15-C16	115.7(4)
C15-C16-N4	116.3(12)	C15-C16-N4	116.4(4)
C16-N4-Ru1	115.5(9)	C16-N4-Ru1	114.3(2)

Table S6 Selected bond lengths (Å) and bond angles (deg) for *trans*-**2** and *cis*-**2**

trans-**1**		*cis*-**1**	
Bond lengths (Å)			
Ru1-Cl1	2.3829(7)	Ru1-Cl1	2.391(3)
		Ru1-Cl2	2.419(2)
Ru1-N1	2.081(2)	Ru1-N1	2.014(8)
Ru1-N2	2.083(2)	Ru1-N2	2.035(7)
		Ru1-N3	2.000(7)
		Ru1-N4	2.065(7)
C1-N1	1.294(3)	C1-N1	1.335(13)
C2-N2	1.300(3)	C2-N2	1.310(12)
C1-C2	1.420(4)	C1-C2	1.425(14)
C3-N1	1.426(4)	C3-N1	1.426(12)
C10-N2	1.430(4)	C10-N2	1.438(12)
		C17-N3	1.289(12)
		C18-N4	1.293(11)
		C17-C18	1.401(14)
		C19-N3	1.465(12)
		C26-N4	1.401(12)
Bond angles (deg)			
		N1-Ru1-Cl1	174.9(2)
		N3-Ru1-Cl2	176.3(2)
		N2-Ru1-N4	174.4(2)
		Cl1-Ru1-Cl2	92.19(9)
N1-Ru1-N2	77.02(8)	N1-Ru1-N2	78.4(3)
Ru1-N1-C1	112.67(18)	Ru1-N1-C1	115.3(6)
N1-C1-C2	117.6(2)	N1-C1-C2	115.3(9)
C1-C2-N2	116.2(2)	C1-C2-N2	115.5(9)
C2-N2-Ru1	113.03(18)	C2-N2-Ru1	115.5(6)
		N3-Ru1-N4	77.3(3)
		Ru1-N3-C17	116.5(9)
		N3-C17-C18	115.4(8)
		C17-C18-N4	116.9(9)
		C18-N4-Ru1	113.1(6)

Table S7 Selected bond lengths (Å) and bond angles (deg) for *trans*-**3** and *cis*-**3**

trans-**3**		*cis*-**3**	
Bond lengths (Å)			
Ru1-Cl1	2.3878(6)	Ru1-Cl1	2.4020(17)
Ru1-N1	2.086(2)	Ru1-Cl2	2.4097(19)
Ru1-N2	2.072(2)	Ru1-N1	2.052(5)
C1-N1	1.298(4)	Ru1-N2	2.063(5)
C2-N2	1.302(4)	Ru1-N3	2.053(5)
C1-C2	1.441(4)	Ru1-N4	2.062(4)
C3-N1	1.429(3)	C1-N1	1.310(7)
C9-N2	1.430(4)	C2-N2	1.301(8)
		C1-C2	1.417(9)
		C3-N1	1.394(8)
		C10-N2	1.417(8)
		C17-N3	1.300(8)
		C18-N4	1.306(9)
		C17-C18	1.412(10)
		C19-N3	1.429(8)
		C26-N4	1.435(8)
Bond angles (deg)			
N1-Ru1-N2	76.80(9)	N1-Ru1-Cl1	176.09(14)
Ru1-N1-C1	113.55(18)	N3-Ru1-Cl2	175.63(13)
N1-C1-C2	116.1(2)	N1-Ru1-N2	79.11(19)
C1-C2-N2	116.0(2)	Ru1-N1-C1	112.5(4)
C2-N2-Ru1	114.00(19)	N1-C1-C2	118.0(6)
		C1-C2-N2	116.8(6)
		C2-N2-Ru1	113.2(4)
		N2-Ru1-N4	171.1(2)
		Cl1-Ru1-Cl2	91.25(6)
		N3-Ru1-N4	78.1(2)
		Ru1-N3-C15	113.7(4)
		N3-C17-C18	117.5(6)
		C17-C18-N4	116.4(6)
		C18-N4-Ru1	113.8(4)

Table S8 Selected bond lengths (Å) and bond angles (deg) for *trans*-**4** and *cis*-**4**

trans-**4**		*cis*-**4**	
Bond lengths (Å)			
Ru1-Cl1	2.388(4)	Ru1-Cl1	2.413(2)
		Ru1-Cl2	2.374(2)
Ru1-N1	2.083(13)	Ru1-N1	2.022(6)
Ru1-N2	2.068(13)	Ru1-N2	2.077(7)
		Ru1-N3	2.041(8)
		Ru1-N4	2.046(6)
C1-N1	1.27(2)	C1-N1	1.296(9)
C2-N2	1.30(2)	C2-N2	1.290(11)
C1-C2	1.43(2)	C1-C2	1.461(12)
C3-N1	1.43(2)	C3-N1	1.417(10)
C9-N2	1.42(2)	C9-N2	1.427(11)
		C15-N3	1.310(10)
		C16-N4	1.319(10)
		C15-C16	1.390(13)
		C17-N3	1.412(12)
		C23-N4	1.410(11)
Bond angles (deg)			
		N1-Ru1-Cl1	176.21(19)
		N3-Ru1-Cl2	173.77(18)
		N2-Ru1-N4	173.2(3)
		Cl1-Ru1-Cl2	91.11(8)
N1-Ru1-N2	77.1(5)	N1-Ru1-N2	77.9(3)
Ru1-N1-C1	113.3(11)	Ru1-N1-C1	116.1(5)
N1-C1-C2	117.1(15)	N1-C1-C2	114.9(7)
C1-C2-N2	116.8(14)	C1-C2-N2	116.2(7)
C2-N2-Ru1	112.9(11)	C2-N2-Ru1	113.8(6)
		N3-Ru1-N4	78.0(3)
		Ru1-N3-C15	113.4(6)
		N3-C15-C16	118.4(8)
		C15-C16-N4	115.0(6)
		C16-N4-Ru1	114.7(5)

Table S9 Cyclic voltammetric data[a]

Complex	*E*/ V *vs*. SCE
trans-**1**	0.50[b](70)[c], -1.01[d]
trans-**2**	0.53[b](65)[c], -1.10[d]
trans-**3**	0.58[b](72)[c], -0.94[d]
trans-**4**	0.69[b](70)[c], -0.76[d]
cis-**1**	0.69[b](66)[c], -1.24[d]
cis-**2**	0.75[b](68)[c], -1.10[d]
cis-**3**	0.84[b](70)[c], -1.05[d]
cis-**4**	0.93[b](71)[c], -0.95[d]

[a] Solvent, acetonitrile; supporting electrolyte, TBHP; scan rate, 50 mVs^{-1}.
[b] $E_{½}$ value, where $E_{½} = ½ (E_{pa} + E_{pc})$; E_{pa} = anodic peak potential and E_{pc} = cathodic peak potential.
[c] $\Delta E_p = E_{pa} - E_{pc}$.
[d] E_{pc} value.

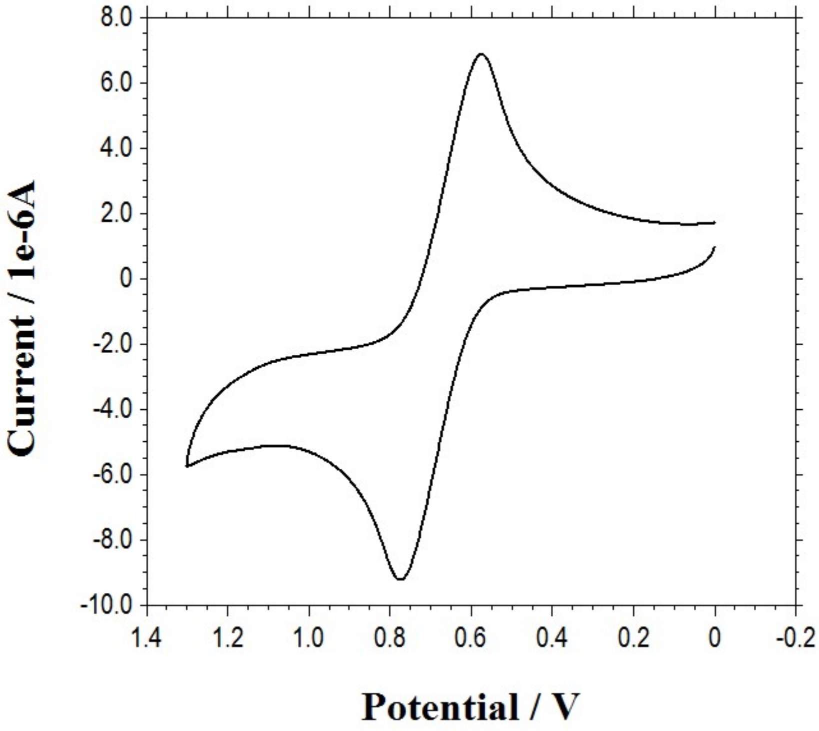

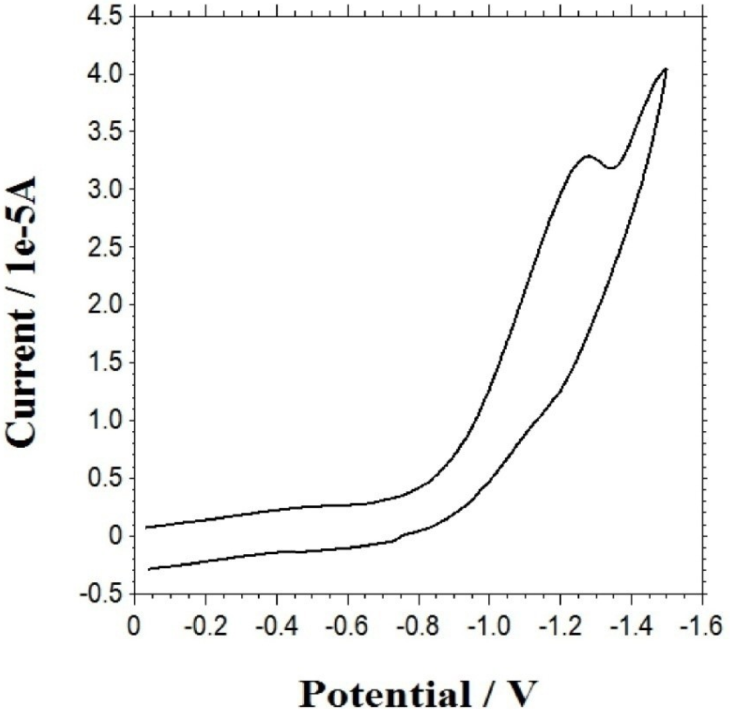

Fig. S4 Cyclic voltammogram of *cis*-**3** in acetonitrile solution (0.1 M TBHP) at a scan rate of 50 mVs^{-1}.

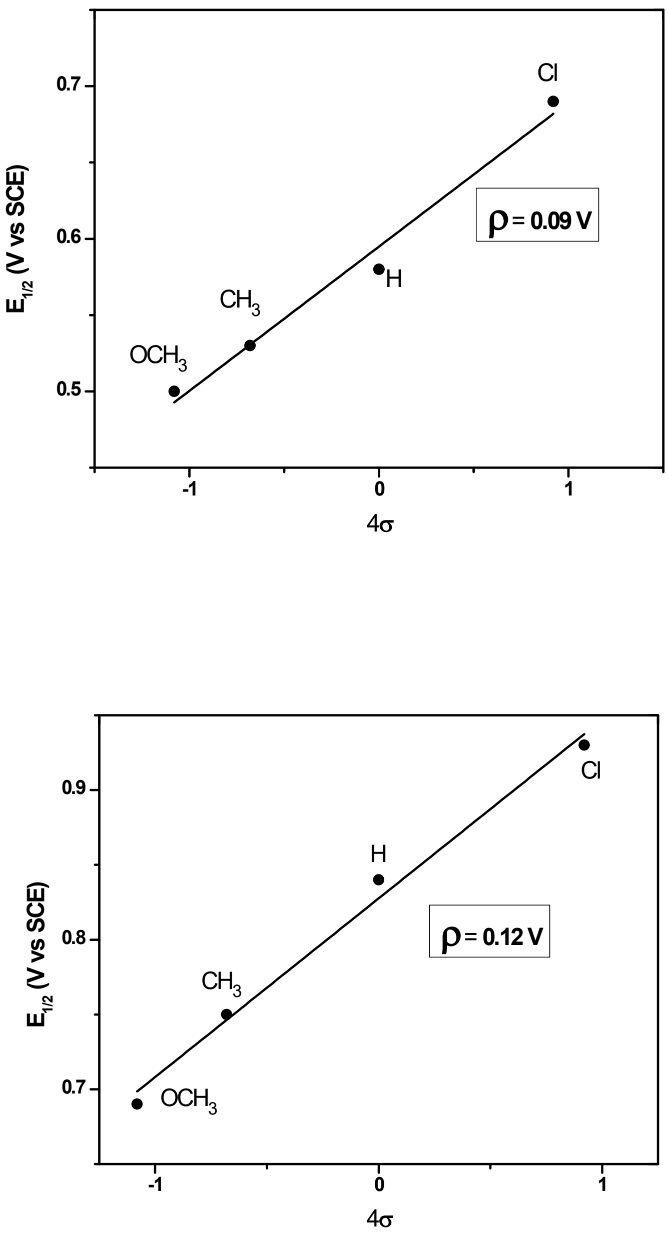

Fig. S5 Least-squares plot of $E_{½}$ values of Ru(II)-Ru(III) couple versus Hammett substituent constant (4σ) for the *trans*-isomers (top) and the *cis*-isomers (bottom).

Table S10 Initial catalytic reactions on benzyl alcohol dehydrogenation and optimization of experimental parameters.[a]

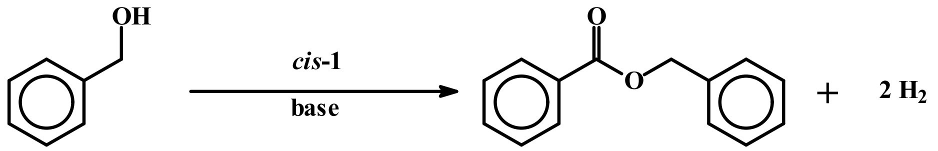

Entry	Solvent	Mole % of catalyst	Base	Base (mol%)	Time, h	Yield[b], %
1	toluene	0.1	KOtBu	1	6	57
2	toluene	0.1	KOtBu	1	10	74
3	toluene	0.1	KO^{t}Bu	1	14	65
4	toluene	0.05	KO^{t}Bu	1	10	48
5	toluene	0.2	KO^{t}Bu	1	10	79
6	toluene	-	KO^{t}Bu	1	10	NO[c]
7	toluene	0.1	-	-	10	15
8	chloroform	0.1	KO^{t}Bu	1	10	NO[c]
9	acetonitrile	0.1	KO^{t}Bu	1	10	NO[c]
10	toluene	0.1	KO^{t}Bu	0.5	10	31
11	toluene	0.1	K_3PO_4	1	10	14
12	toluene	0.1	KOH	1	10	29
13[d]	toluene	0.1	KO^{t}Bu	1	10	43
14[e]	toluene	0.1	KO^{t}Bu	1	10	51
15[f]	toluene	0.1	KO^{t}Bu	1	10	66

[a] Reaction conditions: benzyl alcohol (1.0 mmol), solvent (4.0 mL), refluxing condition.

[b] Determined by GCMS.

[c]Not observed.

[d] The catalyst was *cis*-3.

[e] The catalyst was *cis*-2.

[f] The catalyst was *cis*-4.

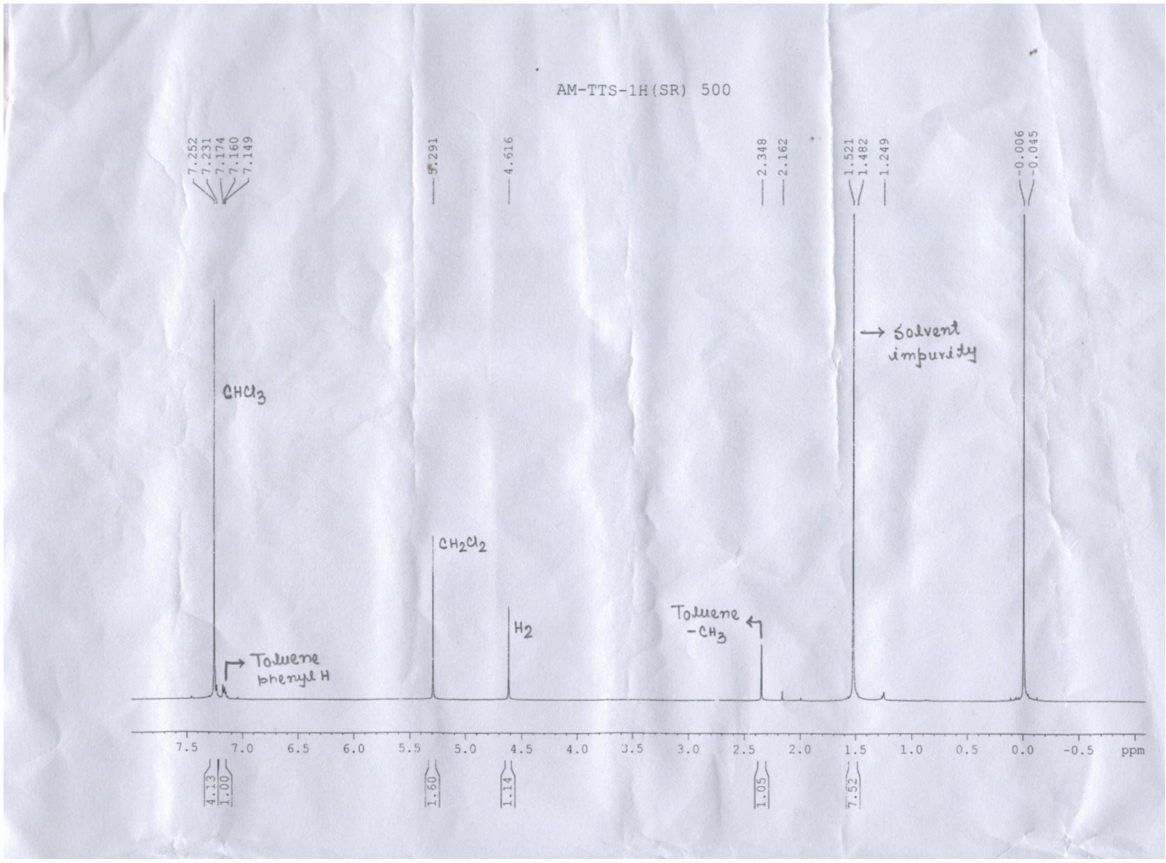

Fig. S6 ^{1}H NMR of H_2 (4.616 ppm) evolved from primary alcohol (benzyl alcohol) oxidation. Peaks arising from toluene and CH_2Cl_2 (the reaction medium), and from impurities in $CDCl_3$ (the NMR solvent) are annotated.

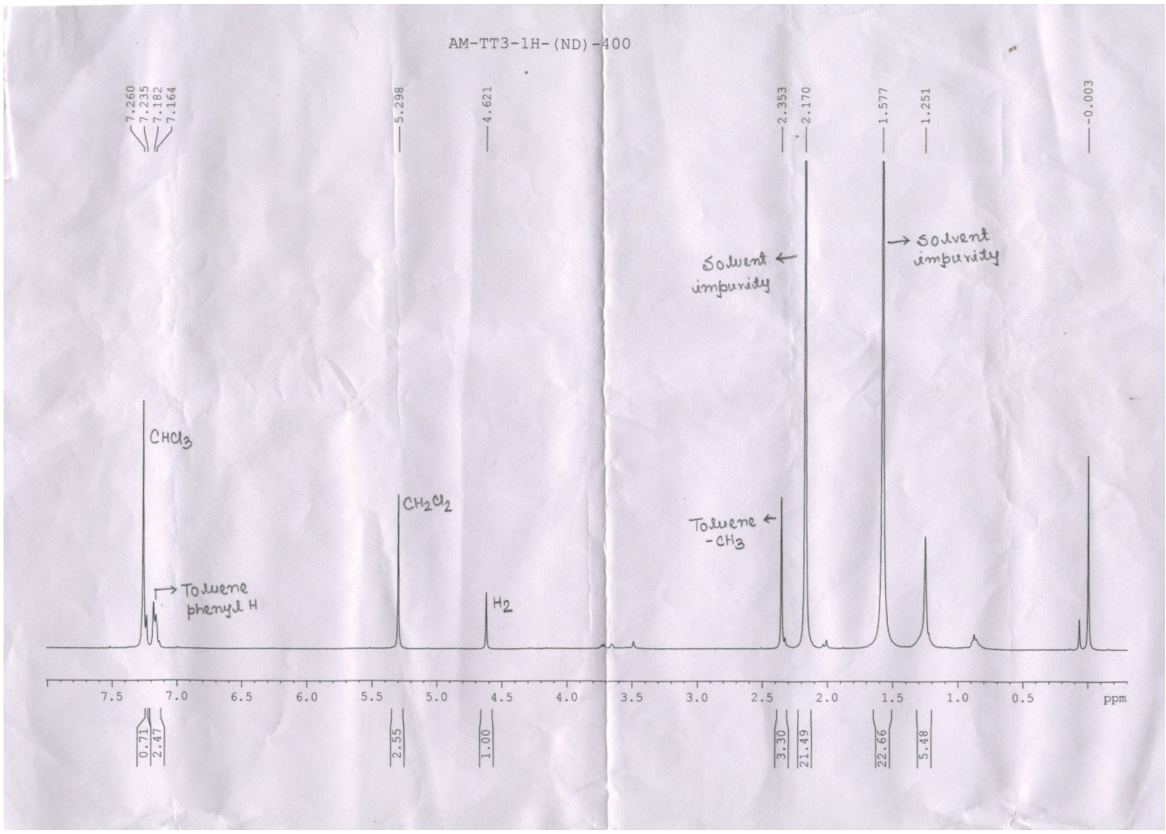

Fig. S7 ^{1}H NMR of H_2 (at 4.621 ppm) evolved from secondary alcohol (cyclohexanol) oxidation. Peaks arising from toluene and CH_2Cl_2 (the reaction medium), and from impurities in $CDCl_3$ (the NMR solvent) are annotated.

Table S10 DFT data

Species A1
HF energy= -2314.74996651
No Imaginary frequency
Zero-point correction= 0.455136 (Hartree/Particle)
Thermal correction to Energy= 0.486487
Thermal correction to Enthalpy= 0.487431
Thermal correction to Gibbs Free Energy= 0.391960
Sum of electronic and zero-point Energies= -2314.294830
Sum of electronic and thermal Energies= -2314.263480
Sum of electronic and thermal Enthalpies= -2314.262536
Sum of electronic and thermal Free Energies= -2314.358006

Coordinates: **A1**

Ru	0.01840000	0.07060000	0.39140000
Cl	-1.03730000	1.53610000	2.00150000
Cl	1.07630000	-1.39360000	-1.22000000
N	1.16370000	-0.70190000	1.96360000
N	1.75130000	1.24670000	0.34850000
N	-1.19610000	0.93900000	-1.07480000
N	-1.65110000	-1.19350000	0.33780000
C	2.30220000	-0.10890000	2.15500000
H	2.96210000	-0.39480000	2.97810000
C	2.63440000	0.95230000	1.25280000
H	3.59550000	1.47050000	1.30070000
C	2.11330000	2.28230000	-0.55370000
C	2.15180000	2.01400000	-1.92240000
H	1.87800000	1.01580000	-2.26910000
C	2.56930000	3.01330000	-2.79320000
H	2.61130000	2.80650000	-3.86250000
C	2.92230000	4.27340000	-2.31080000
H	3.23900000	5.05270000	-3.00360000
C	2.86360000	4.53700000	-0.94500000
H	3.12720000	5.52320000	-0.56350000
C	2.46440000	3.54030000	-0.06020000
H	2.38720000	3.73170000	1.01120000
C	0.84950000	-1.74380000	2.87640000
C	-0.30540000	-1.64740000	3.65360000
H	-0.95470000	-0.77970000	3.52340000
C	-0.57170000	-2.63930000	4.58980000
H	-1.46570000	-2.56370000	5.20860000
C	0.28830000	-3.72770000	4.73500000
H	0.06450000	-4.50480000	5.46550000
C	1.42900000	-3.82380000	3.94260000
H	2.09860000	-4.67750000	4.04400000
C	1.71740000	-2.82840000	3.01410000
H	2.59250000	-2.89450000	2.36590000

C	-0.97870000	2.11760000	-1.83730000
C	-1.01330000	2.05540000	-3.23140000
H	-1.13970000	1.08660000	-3.71700000
C	-0.84290000	3.21980000	-3.97380000
H	-0.85770000	3.17270000	-5.06240000
C	-0.65200000	4.43720000	-3.32600000
H	-0.52220000	5.34990000	-3.90710000
C	-0.62010000	4.48800000	-1.93250000
H	-0.46760000	5.44000000	-1.42440000
C	-0.77110000	3.33010000	-1.17900000
H	-0.75890000	3.33910000	-0.08750000
C	-2.32590000	0.33410000	-1.27960000
H	-3.06100000	0.72490000	-1.98780000
C	-2.56850000	-0.85160000	-0.51420000
H	-3.46610000	-1.45820000	-0.65920000
C	-1.89670000	-2.38870000	1.06500000
C	-0.98350000	-3.44010000	0.97880000
H	-0.08730000	-3.30790000	0.37000000
C	-1.26090000	-4.62950000	1.64160000
H	-0.55700000	-5.45830000	1.56870000
C	-2.42300000	-4.76460000	2.40080000
H	-2.62620000	-5.69860000	2.92430000
C	-3.31970000	-3.70370000	2.49500000
H	-4.22360000	-3.79950000	3.09620000
C	-3.06290000	-2.51310000	1.82230000
H	-3.73890000	-1.66040000	1.90180000

Species A2
HF energy= -2314.75295509
No Imaginary frequency
Zero-point correction= 0.455256 (Hartree/Particle)
Thermal correction to Energy= 0.486419
Thermal correction to Enthalpy= 0.487364
Thermal correction to Gibbs Free Energy= 0.391713
Sum of electronic and zero-point Energies= -2314.297699
Sum of electronic and thermal Energies= -2314.266536
Sum of electronic and thermal Enthalpies= -2314.265592
Sum of electronic and thermal Free Energies= -2314.361242

Coordinates: **A2**

Ru	0.00000000	0.00000000	0.44810000
Cl	-1.06500000	1.24420000	2.17780000
Cl	1.06500000	-1.24420000	2.17780000
N	1.06540000	-0.82550000	-1.11440000
N	1.63650000	1.23460000	0.30040000
N	-1.06540000	0.82550000	-1.11440000
N	-1.63650000	-1.23460000	0.30040000
C	2.08870000	-0.12980000	-1.49590000

H	2.58080000	-0.35740000	-2.25050000
C	2.42050000	1.00140000	-0.70170000
H	3.15610000	1.53950000	-0.89110000
C	2.00450000	2.32630000	1.17890000
C	2.28620000	3.57640000	0.66730000
H	2.22590000	3.73590000	-0.24710000
C	2.65900000	4.58750000	1.53240000
H	2.83360000	5.43870000	1.20090000
C	2.77500000	4.34210000	2.88930000
H	3.02000000	5.02580000	3.46960000
C	2.52610000	3.09530000	3.36910000
H	2.64000000	2.92340000	4.27630000
C	2.10800000	2.07980000	2.53260000
H	1.90090000	1.24150000	2.87600000
C	0.68810000	-1.96570000	-1.89610000
C	0.39830000	-3.14170000	-1.26070000
H	0.42530000	-3.19180000	-0.33220000
C	0.06560000	-4.26080000	-2.01610000
H	-0.11230000	-5.07200000	-1.59800000
C	0.00000000	-4.16020000	-3.37880000
H	-0.24340000	-4.90290000	-3.88310000
C	0.28450000	-2.99910000	-4.00390000
H	0.25190000	-2.95350000	-4.93170000
C	0.62240000	-1.88010000	-3.27680000
H	0.80560000	-1.07600000	-3.70820000
C	-0.68810000	1.96570000	-1.89610000
C	-0.62240000	1.88010000	-3.27680000
H	-0.80560000	1.07600000	-3.70820000
C	-0.28450000	2.99910000	-4.00390000
H	-0.25190000	2.95350000	-4.93170000
C	0.00000000	4.16020000	-3.37880000
H	0.24340000	4.90290000	-3.88310000
C	-0.06560000	4.26080000	-2.01610000
H	0.11230000	5.07200000	-1.59800000
C	-0.39830000	3.14170000	-1.26070000
H	-0.42530000	3.19180000	-0.33220000
C	-2.08870000	0.12980000	-1.49590000
H	-2.58080000	0.35740000	-2.25050000
C	-2.42050000	-1.00140000	-0.70170000
H	-3.15610000	-1.53950000	-0.89110000
C	-2.00450000	-2.32630000	1.17890000
C	-2.28620000	-3.57640000	0.66730000
H	-2.22590000	-3.73590000	-0.24710000
C	-2.65900000	-4.58750000	1.53240000
H	-2.83360000	-5.43870000	1.20090000
C	-2.77500000	-4.34210000	2.88930000
H	-3.02000000	-5.02580000	3.46960000
C	-2.52610000	-3.09530000	3.36910000

H	-2.64000000	-2.92340000	4.27630000
C	-2.10800000	-2.07980000	2.53260000
H	-1.90090000	-1.24150000	2.87600000

Species B
HF energy= -346.502087024
No Imaginary frequency
Zero-point correction= 0.132611 (Hartree/Particle)
Thermal correction to Energy= 0.139759
Thermal correction to Enthalpy= 0.140703
Thermal correction to Gibbs Free Energy= 0.100495
Sum of electronic and zero-point Energies= -346.369476
Sum of electronic and thermal Energies= -346.362328
Sum of electronic and thermal Enthalpies= -346.361384
Sum of electronic and thermal Free Energies= -346.401592

Coordinates: **B**

C	-0.65120000	0.10060000	0.09850000
C	0.74150000	0.09110000	0.15150000
C	1.45260000	1.28510000	0.06510000
C	0.75450000	2.48650000	-0.07490000
C	-0.63410000	2.49670000	-0.12720000
C	-1.34300000	1.29940000	-0.04040000
H	-1.19870000	-0.83980000	0.16630000
H	1.29050000	-0.84220000	0.25940000
H	1.31010000	3.42530000	-0.14370000
H	-1.16630000	3.44180000	-0.23680000
H	-2.43210000	1.30370000	-0.08160000
C	2.95420000	1.31490000	0.12220000
H	3.33900000	1.82050000	-0.78580000
H	3.26930000	1.94090000	0.98070000
O	3.45990000	0.00920000	0.23190000
H	4.41980000	0.05470000	0.27670000

Species C
HF energy= -2200.47219986
No Imaginary frequency
Zero-point correction= 0.576574 (Hartree/Particle)
Thermal correction to Energy= 0.613235
Thermal correction to Enthalpy= 0.614179
Thermal correction to Gibbs Free Energy= 0.507058
Sum of electronic and zero-point Energies= -2199.895625
Sum of electronic and thermal Energies= -2199.858965
Sum of electronic and thermal Enthalpies= -2199.858021
Sum of electronic and thermal Free Energies= -2199.965142

Coordinates: **C**

Ru	0.62860000	2.34290000	2.72910000

O	2.60620000	1.87540000	2.74170000
Cl	0.85660000	3.18960000	4.99430000
N	0.70770000	1.54780000	0.87900000
N	1.06950000	3.99880000	1.56870000
N	-1.37270000	2.67200000	2.97760000
N	-0.01650000	0.60020000	3.67070000
C	1.37740000	2.28850000	0.02270000
H	1.70830000	1.88850000	-0.93890000
C	1.53460000	3.64660000	0.38320000
H	1.96930000	4.38040000	-0.29880000
C	0.91810000	5.38610000	1.82020000
C	0.47360000	6.24350000	0.80430000
H	0.19620000	5.82970000	-0.16580000
C	0.33190000	7.60420000	1.04650000
H	-0.02870000	8.25680000	0.25140000
C	0.63050000	8.12560000	2.30260000
H	0.51610000	9.19230000	2.49400000
C	1.06310000	7.27310000	3.31590000
H	1.29430000	7.67040000	4.30400000
C	1.20060000	5.90940000	3.08530000
H	1.51420000	5.23370000	3.87950000
C	0.45520000	0.18510000	0.59550000
C	1.51880000	-0.70260000	0.42350000
H	2.53740000	-0.32710000	0.52600000
C	1.25590000	-2.04720000	0.18030000
H	2.08530000	-2.74430000	0.06430000
C	-0.05760000	-2.50290000	0.09730000
H	-0.25850000	-3.55590000	-0.09840000
C	-1.11540000	-1.61200000	0.27460000
H	-2.14470000	-1.96500000	0.20940000
C	-0.86340000	-0.26980000	0.53730000
H	-1.67530000	0.44360000	0.68850000
C	-2.02980000	3.85650000	2.56440000
C	-2.22510000	4.08440000	1.20100000
H	-1.88410000	3.33480000	0.48470000
C	-2.84900000	5.25460000	0.78570000
H	-3.01630000	5.42840000	-0.27730000
C	-3.24550000	6.20920000	1.72150000
H	-3.71700000	7.13390000	1.39050000
C	-3.02600000	5.98530000	3.07800000
H	-3.31840000	6.73660000	3.81080000
C	-2.42710000	4.80450000	3.50770000
H	-2.21400000	4.62210000	4.56160000
C	-1.97500000	1.84620000	3.79540000
H	-2.99670000	2.02540000	4.13870000
C	-1.23460000	0.68140000	4.14470000
H	-1.66620000	-0.12330000	4.74400000
C	0.69330000	-0.61400000	3.83690000

C	0.05590000	-1.84050000	3.61750000
H	-0.97820000	-1.85570000	3.27100000
C	0.76260000	-3.02710000	3.77150000
H	0.26650000	-3.97810000	3.57780000
C	2.10180000	-2.99580000	4.15210000
H	2.65720000	-3.92590000	4.27170000
C	2.73160000	-1.77200000	4.37340000
H	3.77850000	-1.74480000	4.67490000
C	2.03980000	-0.57790000	4.21130000
H	2.51500000	0.39230000	4.34040000
C	5.20040000	3.38760000	-0.30190000
C	4.66290000	2.75180000	0.81140000
C	4.22920000	3.48620000	1.92040000
C	4.37260000	4.87250000	1.89580000
C	4.90400000	5.51920000	0.77950000
C	5.31480000	4.77910000	-0.32510000
H	5.53710000	2.79910000	-1.15670000
H	4.55530000	1.66510000	0.83160000
H	4.03750000	5.45860000	2.75490000
H	4.99160000	6.60620000	0.77370000
H	5.73150000	5.28090000	-1.19880000
C	3.60400000	2.76980000	3.10160000
H	4.40520000	2.19490000	3.61540000
H	3.25870000	3.51790000	3.84170000

Species D
HF energy= -460.756039346
No Imaginary frequency
Zero-point correction= 0.006754 (Hartree/Particle)
Thermal correction to Energy= 0.009115
Thermal correction to Enthalpy= 0.010059
Thermal correction to Gibbs Free Energy= -0.011134
Sum of electronic and zero-point Energies= -460.749285
Sum of electronic and thermal Energies= -460.746925
Sum of electronic and thermal Enthalpies= -460.745980
Sum of electronic and thermal Free Energies= -460.767174

Coordinates: **D**

Cl	-0.41530000	-0.11820000	0.00000000
H	-1.70210000	-0.11820000	0.00000000

Species E
HF energy= -1739.64576610
No Imaginary frequency
Zero-point correction= 0.562130 (Hartree/Particle)
Thermal correction to Energy= 0.597199
Thermal correction to Enthalpy= 0.598144
Thermal correction to Gibbs Free Energy= 0.494603

Sum of electronic and zero-point Energies= -1739.083636
Sum of electronic and thermal Energies= -1739.048567
Sum of electronic and thermal Enthalpies= -1739.047622
Sum of electronic and thermal Free Energies= -1739.151163

Coordinates: **E**

Ru	-0.48520000	-0.04960000	0.01860000
N	0.81760000	-1.26760000	-0.76430000
N	1.36790000	0.79760000	0.62230000
N	-0.95360000	1.29300000	-1.30440000
N	-2.25000000	-0.81540000	-0.76690000
C	2.13640000	-0.97770000	-0.65100000
H	2.87950000	-1.58310000	-1.16840000
C	2.41440000	0.14240000	0.11330000
H	3.43380000	0.49010000	0.30220000
C	1.57380000	1.95840000	1.38690000
C	2.48480000	2.93840000	0.97530000
H	3.00340000	2.81140000	0.02390000
C	2.67140000	4.09010000	1.73140000
H	3.37360000	4.84840000	1.38240000
C	1.95500000	4.28610000	2.90870000
H	2.10120000	5.19060000	3.49830000
C	1.04250000	3.31530000	3.32360000
H	0.47660000	3.45760000	4.24490000
C	0.84600000	2.16360000	2.57190000
H	0.15000000	1.39410000	2.89870000
C	0.47890000	-2.40850000	-1.54000000
C	0.87260000	-3.67980000	-1.10510000
H	1.42650000	-3.76870000	-0.16720000
C	0.52720000	-4.80720000	-1.84560000
H	0.82590000	-5.79560000	-1.49160000
C	-0.20020000	-4.67410000	-3.02730000
H	-0.47060000	-5.55560000	-3.60820000
C	-0.57480000	-3.40400000	-3.46720000
H	-1.13260000	-3.29020000	-4.39800000
C	-0.23510000	-2.27220000	-2.73350000
H	-0.52750000	-1.27500000	-3.06810000
C	-0.07590000	2.34370000	-1.68250000
C	0.89550000	2.14790000	-2.66580000
H	0.97110000	1.17040000	-3.14450000
C	1.74510000	3.19460000	-3.01680000
H	2.50250000	3.03850000	-3.78560000
C	1.62190000	4.43990000	-2.39820000
H	2.28270000	5.25920000	-2.68070000
C	0.65010000	4.63270000	-1.41920000
H	0.55320000	5.59860000	-0.92230000
C	-0.19270000	3.58740000	-1.05490000
H	-0.94820000	3.71540000	-0.27710000

C	-1.97170000	0.99340000	-2.17440000
H	-2.14140000	1.61630000	-3.05250000
C	-2.70420000	-0.12150000	-1.83420000
H	-3.56670000	-0.46820000	-2.40500000
C	-3.02220000	-1.85370000	-0.23130000
C	-2.38750000	-2.91340000	0.43150000
H	-1.29840000	-2.93380000	0.45180000
C	-3.12950000	-3.91010000	1.05090000
H	-2.60970000	-4.72640000	1.55470000
C	-4.52370000	-3.88180000	1.02360000
H	-5.09970000	-4.66500000	1.51550000
C	-5.16720000	-2.83870000	0.36020000
H	-6.25630000	-2.78990000	0.33990000
C	-4.42980000	-1.83500000	-0.25950000
H	-4.94700000	-0.99370000	-0.72340000
C	-5.61660000	0.03030000	3.69860000
C	-4.32040000	-0.46380000	3.73000000
C	-3.40690000	-0.12820000	2.72100000
C	-3.80930000	0.72070000	1.67580000
C	-5.09910000	1.23220000	1.66220000
C	-6.00430000	0.88170000	2.66420000
H	-6.32610000	-0.24180000	4.47910000
H	-4.00000000	-1.12710000	4.53610000
H	-3.10290000	0.97080000	0.88110000
H	-5.40840000	1.89380000	0.85310000
H	-7.02090000	1.27460000	2.63820000
C	-2.06430000	-0.68440000	2.81220000
H	-1.86780000	-1.29330000	3.71890000
O	-1.13130000	-0.54380000	2.01690000

Species F
HF energy= -345.313062600
No Imaginary frequency
Zero-point correction= 0.109463 (Hartree/Particle)
Thermal correction to Energy= 0.115805
Thermal correction to Enthalpy= 0.116749
Thermal correction to Gibbs Free Energy= 0.078920
Sum of electronic and zero-point Energies= -345.203600
Sum of electronic and thermal Energies= -345.197258
Sum of electronic and thermal Enthalpies= -345.196313
Sum of electronic and thermal Free Energies= -345.234142

Coordinates: **F**

C	-1.11170000	-0.30150000	0.26120000
C	0.27880000	-0.28370000	0.26870000
C	0.96410000	0.90350000	0.00690000
C	0.25640000	2.07870000	-0.26380000
C	-1.13070000	2.06130000	-0.27160000

C	-1.81310000	0.87150000	-0.00910000
H	-1.65000000	-1.22640000	0.46510000
H	0.84560000	-1.19350000	0.47840000
H	0.82150000	2.98880000	-0.46420000
H	-1.68830000	2.97350000	-0.48170000
H	-2.90300000	0.86040000	-0.01570000
C	2.43910000	0.91610000	0.01610000
H	2.90340000	-0.07800000	0.24380000
O	3.12230000	1.88820000	-0.19720000

Species ^{3}G

HF energy= -1394.31073738

No Imaginary frequency

Zero-point correction= 0.450171 (Hartree/Particle)

Thermal correction to Energy= 0.477999

Thermal correction to Enthalpy= 0.478943

Thermal correction to Gibbs Free Energy= 0.390024

Sum of electronic and zero-point Energies= -1393.860566

Sum of electronic and thermal Energies= -1393.832739

Sum of electronic and thermal Enthalpies= -1393.831794

Sum of electronic and thermal Free Energies= -1393.920714

Coordinates: **^{3}G**

Ru	-0.00010000	0.00000000	-0.43860000
N	1.32620000	-1.56260000	-0.69020000
N	1.82470000	0.93300000	-0.18660000
N	-1.32570000	1.56270000	-0.69310000
N	-1.82570000	-0.93320000	-0.19280000
C	2.61660000	-1.21460000	-0.58930000
H	3.40950000	-1.94240000	-0.77130000
C	2.88260000	0.11680000	-0.29150000
H	3.89460000	0.48450000	-0.11170000
C	2.08400000	2.27710000	0.16580000
C	3.13470000	2.99530000	-0.41750000
H	3.75390000	2.51700000	-1.17760000
C	3.35440000	4.32280000	-0.06740000
H	4.16830000	4.87440000	-0.53830000
C	2.53180000	4.95020000	0.86490000
H	2.70110000	5.99250000	1.13300000
C	1.48540000	4.23680000	1.44620000
H	0.83050000	4.71970000	2.17160000
C	1.25950000	2.91050000	1.10010000
H	0.44030000	2.34120000	1.54290000
C	1.04920000	-2.90420000	-1.03840000
C	1.74670000	-3.96860000	-0.45480000
H	2.50440000	-3.76210000	0.30230000
C	1.44050000	-5.28000000	-0.80100000
H	1.98320000	-6.10000000	-0.33030000

C	0.43730000	-5.54680000	-1.72910000
H	0.19390000	-6.57520000	-1.99420000
C	-0.25900000	-4.48850000	-2.30990000
H	-1.05140000	-4.68580000	-3.03200000
C	0.04180000	-3.17600000	-1.96860000
H	-0.49700000	-2.33730000	-2.41310000
C	-1.04780000	2.90460000	-1.03910000
C	-0.03720000	3.17750000	-1.96560000
H	0.50330000	2.33950000	-2.40910000
C	0.26460000	4.49050000	-2.30440000
H	1.05950000	4.68850000	-3.02360000
C	-0.43390000	5.54800000	-1.72500000
H	-0.18970000	6.57670000	-1.98810000
C	-1.44030000	5.28010000	-0.80060000
H	-1.98480000	6.09950000	-0.33110000
C	-1.74740000	3.96830000	-0.45680000
H	-2.50770000	3.76100000	0.29740000
C	-2.61640000	1.21450000	-0.59650000
H	-3.40870000	1.94240000	-0.78060000
C	-2.88330000	-0.11710000	-0.30050000
H	-3.89580000	-0.48500000	-0.12410000
C	-2.08570000	-2.27750000	0.15800000
C	-3.13520000	-2.99540000	-0.42790000
H	-3.75290000	-2.51670000	-1.18890000
C	-3.35550000	-4.32320000	-0.07910000
H	-4.16840000	-4.87450000	-0.55210000
C	-2.53480000	-4.95120000	0.85430000
H	-2.70460000	-5.99370000	1.12130000
C	-1.48940000	-4.23820000	1.43810000
H	-0.83600000	-4.72160000	2.16450000
C	-1.26300000	-2.91170000	1.09350000
H	-0.44480000	-2.34260000	1.53830000

Species ^{1}**G**
HF energy= -1394.29281003
No Imaginary frequency
Zero-point correction= 0.449700 (Hartree/Particle)
Thermal correction to Energy= 0.477665
Thermal correction to Enthalpy= 0.478609
Thermal correction to Gibbs Free Energy= 0.387935
Sum of electronic and zero-point Energies= -1393.843110
Sum of electronic and thermal Energies= -1393.815145
Sum of electronic and thermal Enthalpies= -1393.814201
Sum of electronic and thermal Free Energies= -1393.904876

Coordinates: ^{1}**G**

Ru	0.00000000	0.00000000	0.13750000

N	1.18310000	-1.01350000	-1.06300000
N	1.65800000	1.22270000	0.04040000
N	-1.18330000	1.01360000	-1.06280000
N	-1.65810000	-1.22260000	0.04060000
C	2.15720000	-0.26990000	-1.64100000
H	2.69710000	-0.64630000	-2.51000000
C	2.45550000	0.92270000	-0.99090000
H	3.32980000	1.52430000	-1.24450000
C	1.93390000	2.30080000	0.89410000
C	2.65830000	3.43220000	0.49710000
H	3.02490000	3.51090000	-0.52660000
C	2.86220000	4.48260000	1.38680000
H	3.41900000	5.35970000	1.05580000
C	2.35520000	4.42810000	2.68180000
H	2.51530000	5.25520000	3.37220000
C	1.62450000	3.30750000	3.08000000
H	1.21460000	3.25080000	4.08840000
C	1.40660000	2.26360000	2.19330000
H	0.81880000	1.38970000	2.49250000
C	0.76910000	-2.21070000	-1.69890000
C	0.59140000	-3.36700000	-0.93610000
H	0.80480000	-3.32980000	0.13290000
C	0.13250000	-4.53320000	-1.53630000
H	-0.01140000	-5.42700000	-0.92930000
C	-0.15400000	-4.55620000	-2.90040000
H	-0.51960000	-5.47010000	-3.36780000
C	0.02570000	-3.40470000	-3.66360000
H	-0.20540000	-3.41270000	-4.72880000
C	0.48620000	-2.23470000	-3.06800000
H	0.60340000	-1.31830000	-3.64890000
C	-0.76930000	2.21080000	-1.69880000
C	-0.48670000	2.23480000	-3.06790000
H	-0.60410000	1.31840000	-3.64880000
C	-0.02610000	3.40480000	-3.66360000
H	0.20480000	3.41280000	-4.72880000
C	0.15390000	4.55620000	-2.90040000
H	0.51960000	5.47010000	-3.36780000
C	-0.13240000	4.53320000	-1.53630000
H	0.01170000	5.42700000	-0.92920000
C	-0.59140000	3.36710000	-0.93600000
H	-0.80460000	3.32990000	0.13300000
C	-2.15750000	0.27010000	-1.64070000
H	-2.69760000	0.64650000	-2.50960000
C	-2.45570000	-0.92260000	-0.99060000
H	-3.33010000	-1.52410000	-1.24410000
C	-1.93370000	-2.30080000	0.89420000
C	-2.65800000	-3.43230000	0.49700000
H	-3.02450000	-3.51090000	-0.52670000

C	-2.86170000	-4.48290000	1.38660000
H	-3.41830000	-5.36000000	1.05540000
C	-2.35470000	-4.42840000	2.68160000
H	-2.51480000	-5.25560000	3.37190000
C	-1.62430000	-3.30760000	3.07990000
H	-1.21440000	-3.25100000	4.08840000
C	-1.40650000	-2.26370000	2.19340000
H	-0.81900000	-1.38960000	2.49270000

Species H

HF energy= -1740.83802348

No Imaginary frequency

Zero-point correction= 0.586712 (Hartree/Particle)

Thermal correction to Energy= 0.621970

Thermal correction to Enthalpy= 0.622914

Thermal correction to Gibbs Free Energy= 0.519433

Sum of electronic and zero-point Energies= -1740.251312

Sum of electronic and thermal Energies= -1740.216054

Sum of electronic and thermal Enthalpies= -1740.215110

Sum of electronic and thermal Free Energies= -1740.318590

Coordinates: **H**

Ru	-0.45760000	0.40220000	0.38810000
N	0.71090000	-0.53880000	-0.90790000
N	1.29470000	1.45860000	0.54580000
N	-1.38560000	1.45050000	-1.02490000
N	-2.11090000	-0.78520000	0.01540000
C	1.81610000	0.16200000	-1.28620000
H	2.45770000	-0.20790000	-2.08590000
C	2.13250000	1.24370000	-0.49150000
H	3.03890000	1.84000000	-0.61300000
C	1.52910000	2.52020000	1.42520000
C	1.77010000	3.82130000	0.95720000
H	1.79710000	3.99590000	-0.11960000
C	1.92820000	4.87180000	1.85350000
H	2.10520000	5.87740000	1.47040000
C	1.84800000	4.65550000	3.22900000
H	1.96900000	5.48430000	3.92530000
C	1.62830000	3.36460000	3.70380000
H	1.59620000	3.17420000	4.77690000
C	1.47730000	2.30310000	2.81350000
H	1.37730000	1.27570000	3.17450000
C	0.36410000	-1.69640000	-1.64270000
C	-0.10890000	-2.82710000	-0.96930000
H	-0.15160000	-2.80760000	0.11860000
C	-0.52990000	-3.94670000	-1.67690000
H	-0.90840000	-4.81180000	-1.13200000
C	-0.46120000	-3.96850000	-3.06820000

H	-0.79030000	-4.84690000	-3.62280000
C	0.04070000	-2.85830000	-3.74430000
H	0.09850000	-2.86090000	-4.83300000
C	0.45020000	-1.73140000	-3.04040000
H	0.80650000	-0.85110000	-3.57770000
C	-0.82600000	2.60860000	-1.62060000
C	-0.03940000	2.51160000	-2.77430000
H	0.12870000	1.52560000	-3.20870000
C	0.53030000	3.65150000	-3.33240000
H	1.15000000	3.56300000	-4.22460000
C	0.31560000	4.90050000	-2.75320000
H	0.76060000	5.79230000	-3.19400000
C	-0.47360000	5.00300000	-1.60920000
H	-0.65070000	5.97590000	-1.15030000
C	-1.03590000	3.86320000	-1.04380000
H	-1.64960000	3.93290000	-0.14550000
C	-2.17740000	0.65930000	-1.78980000
H	-2.46060000	0.97280000	-2.79620000
C	-2.62970000	-0.50850000	-1.19190000
H	-3.36050000	-1.15870000	-1.67570000
C	-2.67950000	-1.74250000	0.85860000
C	-3.91280000	-2.36840000	0.61530000
H	-4.50220000	-2.09900000	-0.25990000
C	-4.42280000	-3.29760000	1.51300000
H	-5.38410000	-3.76820000	1.30280000
C	-3.73350000	-3.61520000	2.68190000
H	-4.14050000	-4.34370000	3.38260000
C	-2.50980000	-2.99740000	2.93530000
H	-1.94890000	-3.24190000	3.83780000
C	-1.98130000	-2.09070000	2.02730000
H	-1.00470000	-1.62910000	2.20080000
C	-5.40810000	-0.12250000	2.85180000
C	-4.19940000	0.54200000	3.01570000
C	-3.74800000	1.44010000	2.04440000
C	-4.51610000	1.65170000	0.90160000
C	-5.73040000	0.98890000	0.73480000
C	-6.17860000	0.10540000	1.71160000
H	-5.74990000	-0.82510000	3.61150000
H	-3.58830000	0.36210000	3.90240000
H	-4.15660000	2.34380000	0.13720000
H	-6.32620000	1.16680000	-0.16050000
H	-7.13020000	-0.41150000	1.58500000
C	-2.48170000	2.20430000	2.26260000
H	-2.34720000	2.95990000	1.47220000
H	-2.51670000	2.72800000	3.23260000
O	-1.34460000	1.33190000	2.28630000
H	-0.59290000	1.84320000	2.63710000

Species TSHI
HF energy= -1740.78766817
One imaginary frequency (1356*i*)
Zero-point correction= 0.581330 (Hartree/Particle)
Thermal correction to Energy= 0.616267
Thermal correction to Enthalpy= 0.617212
Thermal correction to Gibbs Free Energy= 0.514600
Sum of electronic and zero-point Energies= -1740.206338
Sum of electronic and thermal Energies= -1740.171401
Sum of electronic and thermal Enthalpies= -1740.170457
Sum of electronic and thermal Free Energies= -1740.273068

Coordinates: **TSHI**

Ru	-0.54500000	0.13140000	0.73200000
N	0.83680000	-0.53640000	-0.60990000
N	1.07730000	1.23580000	1.19050000
N	-1.81250000	0.66360000	-0.90740000
N	-2.04060000	-1.32570000	0.68920000
C	2.02140000	0.10590000	-0.58530000
H	2.79530000	-0.13350000	-1.31530000
C	2.17110000	1.04040000	0.42030000
H	3.09590000	1.57900000	0.62900000
C	1.13530000	2.30330000	2.12140000
C	1.41650000	3.60080000	1.67660000
H	1.62320000	3.76160000	0.61640000
C	1.40780000	4.66640000	2.57120000
H	1.61770000	5.67390000	2.21180000
C	1.12890000	4.44660000	3.91730000
H	1.11870000	5.28100000	4.61790000
C	0.87220000	3.15170000	4.36500000
H	0.66620000	2.96980000	5.41970000
C	0.87500000	2.08300000	3.47600000
H	0.66610000	1.06870000	3.81210000
C	0.51000000	-1.27370000	-1.77010000
C	-0.15640000	-2.49960000	-1.66770000
H	-0.37950000	-2.89640000	-0.67760000
C	-0.51380000	-3.20240000	-2.81170000
H	-1.02060000	-4.16260000	-2.71320000
C	-0.22020000	-2.69360000	-4.07650000
H	-0.50430000	-3.24770000	-4.97080000
C	0.43800000	-1.47180000	-4.18510000
H	0.66130000	-1.05460000	-5.16750000
C	0.79890000	-0.76350000	-3.04350000
H	1.27030000	0.21750000	-3.12740000
C	-1.46300000	1.62760000	-1.86500000
C	-1.76730000	1.45540000	-3.22100000
H	-2.26780000	0.54390000	-3.54990000
C	-1.35970000	2.40560000	-4.15020000

H	-1.58820000	2.25590000	-5.20540000
C	-0.64340000	3.52900000	-3.74240000
H	-0.31880000	4.26680000	-4.47540000
C	-0.32300000	3.69080000	-2.39440000
H	0.24810000	4.56030000	-2.06760000
C	-0.71840000	2.74150000	-1.46170000
H	-0.45950000	2.83220000	-0.40680000
C	-2.87800000	-0.04880000	-1.05130000
H	-3.62840000	0.17130000	-1.81440000
C	-3.02400000	-1.13130000	-0.14460000
H	-3.89520000	-1.78960000	-0.17500000
C	-2.15330000	-2.35780000	1.64780000
C	-3.38110000	-2.66980000	2.24290000
H	-4.26380000	-2.07710000	1.99950000
C	-3.46010000	-3.69320000	3.18180000
H	-4.41710000	-3.91600000	3.65300000
C	-2.32310000	-4.41660000	3.53010000
H	-2.38810000	-5.21640000	4.26700000
C	-1.09560000	-4.09500000	2.94970000
H	-0.19810000	-4.64640000	3.22860000
C	-1.00360000	-3.06080000	2.02820000
H	-0.04550000	-2.76710000	1.59800000
C	-5.56810000	1.39510000	0.84620000
C	-4.43880000	1.34180000	1.65500000
C	-3.41950000	2.29050000	1.52620000
C	-3.57080000	3.30610000	0.58360000
C	-4.69770000	3.36420000	-0.23560000
C	-5.69720000	2.40510000	-0.11150000
H	-6.35510000	0.64700000	0.95990000
H	-4.31480000	0.54530000	2.39140000
H	-2.78230000	4.05450000	0.47520000
H	-4.78940000	4.15820000	-0.97770000
H	-6.58110000	2.44710000	-0.74880000
C	-2.17870000	2.17090000	2.38200000
H	-1.43130000	2.92300000	2.05410000
H	-2.43090000	2.43020000	3.42950000
O	-1.66950000	0.85990000	2.39340000
H	-0.62790000	-0.04960000	2.33500000

Species I

HF energy= -1740.83327220

No Imaginary frequency

Zero-point correction= 0.583429 (Hartree/Particle)

Thermal correction to Energy= 0.618470

Thermal correction to Enthalpy= 0.619414

Thermal correction to Gibbs Free Energy= 0.516458

Sum of electronic and zero-point Energies= -1740.249843

Sum of electronic and thermal Energies= -1740.214802

Sum of electronic and thermal Enthalpies= -1740.213858
Sum of electronic and thermal Free Energies= -1740.316814

Coordinates: **I**

Ru	-0.46750000	0.13060000	0.82300000
N	0.84620000	-0.49800000	-0.64700000
N	1.14620000	1.37790000	1.04200000
N	-1.67930000	0.87600000	-0.88200000
N	-1.99250000	-1.22290000	0.64880000
C	1.97270000	0.18360000	-0.74940000
H	2.69460000	-0.02810000	-1.54030000
C	2.16010000	1.19170000	0.21890000
H	3.07900000	1.77360000	0.31000000
C	1.24320000	2.42170000	1.99650000
C	1.61750000	3.70650000	1.59000000
H	1.84230000	3.88870000	0.53740000
C	1.66110000	4.74370000	2.51650000
H	1.93900000	5.74620000	2.19190000
C	1.34250000	4.49950000	3.84920000
H	1.37320000	5.31140000	4.57540000
C	0.98370000	3.21400000	4.25260000
H	0.73910000	3.01870000	5.29630000
C	0.92350000	2.17270000	3.33440000
H	0.62070000	1.17000000	3.62410000
C	0.49580000	-1.36790000	-1.70870000
C	-0.03160000	-2.63220000	-1.43210000
H	-0.13720000	-2.94620000	-0.39480000
C	-0.40780000	-3.47080000	-2.47400000
H	-0.80440000	-4.46030000	-2.24820000
C	-0.28550000	-3.05180000	-3.79810000
H	-0.59320000	-3.70860000	-4.61100000
C	0.22460000	-1.78650000	-4.07530000
H	0.31170000	-1.44350000	-5.10620000
C	0.61810000	-0.94660000	-3.03810000
H	0.98430000	0.05950000	-3.24750000
C	-1.31230000	1.88600000	-1.78380000
C	-1.50610000	1.77340000	-3.16950000
H	-1.92830000	0.85760000	-3.58440000
C	-1.11300000	2.80310000	-4.01680000
H	-1.26260000	2.69920000	-5.09150000
C	-0.52080000	3.95490000	-3.50290000
H	-0.21460000	4.75880000	-4.17150000
C	-0.30800000	4.06300000	-2.12930000
H	0.15680000	4.95850000	-1.71560000
C	-0.68590000	3.03310000	-1.27830000
H	-0.52170000	3.10060000	-0.20230000
C	-2.68740000	0.08620000	-1.15970000
H	-3.35690000	0.27420000	-2.00430000

C	-2.88900000	-1.01780000	-0.30180000
H	-3.73030000	-1.69880000	-0.44020000
C	-2.25340000	-2.26830000	1.57160000
C	-3.56230000	-2.55030000	1.98510000
H	-4.38240000	-1.92160000	1.63790000
C	-3.81060000	-3.58790000	2.87680000
H	-4.83280000	-3.78190000	3.20070000
C	-2.76250000	-4.36280000	3.36400000
H	-2.95840000	-5.17500000	4.06330000
C	-1.45750000	-4.07550000	2.96860000
H	-0.62630000	-4.66470000	3.35490000
C	-1.20030000	-3.02960000	2.09210000
H	-0.17930000	-2.77720000	1.80970000
C	-5.53450000	0.94370000	1.12290000
C	-4.31370000	1.07390000	1.77730000
C	-3.44340000	2.12260000	1.46620000
C	-3.83380000	3.04770000	0.49870000
C	-5.05120000	2.91800000	-0.16820000
C	-5.90380000	1.86030000	0.13750000
H	-6.20810000	0.12450000	1.38320000
H	-4.00470000	0.35410000	2.53820000
H	-3.16500000	3.87610000	0.25210000
H	-5.33270000	3.64440000	-0.93150000
H	-6.85860000	1.75670000	-0.37900000
C	-2.11420000	2.22900000	2.18860000
H	-1.49300000	3.00110000	1.67390000
H	-2.30510000	2.65740000	3.19650000
O	-1.46770000	1.02500000	2.36030000
H	0.15720000	-0.69230000	2.05310000

Species TSIJ
HF energy= -1740.79407888
One imaginary frequency (1205*i*)
Zero-point correction= 0.580949 (Hartree/Particle)
Thermal correction to Energy= 0.615720
Thermal correction to Enthalpy= 0.616664
Thermal correction to Gibbs Free Energy= 0.513291
Sum of electronic and zero-point Energies= -1740.213130
Sum of electronic and thermal Energies= -1740.178359
Sum of electronic and thermal Enthalpies= -1740.177415
Sum of electronic and thermal Free Energies= -1740.280788

Coordinates: **TSIJ**

Ru	-1.32110000	0.08740000	0.52960000
N	0.23400000	-0.85710000	-0.25210000
N	1.94730000	1.13350000	1.17430000
N	-1.76090000	1.35750000	-0.88620000
N	-2.81760000	-0.83460000	-0.39700000

C	1.26220000	-0.11600000	-0.80600000
H	1.51090000	-0.29030000	-1.85710000
C	2.02530000	0.79890000	-0.14340000
H	2.77420000	1.33210000	-0.72630000
C	2.60650000	2.19630000	1.78580000
C	3.64480000	2.91940000	1.18300000
H	4.00200000	2.65780000	0.18790000
C	4.23500000	3.98230000	1.85890000
H	5.04230000	4.53150000	1.37360000
C	3.81590000	4.34610000	3.13580000
H	4.28630000	5.17970000	3.65520000
C	2.79050000	3.62020000	3.73990000
H	2.44990000	3.88310000	4.74150000
C	2.19050000	2.55970000	3.07620000
H	1.39110000	1.98670000	3.54960000
C	-0.10520000	-2.02740000	-0.98940000
C	-0.14480000	-3.25470000	-0.32760000
H	0.09560000	-3.27730000	0.73610000
C	-0.48570000	-4.41410000	-1.01900000
H	-0.51830000	-5.36650000	-0.49010000
C	-0.79460000	-4.35430000	-2.37400000
H	-1.06500000	-5.26090000	-2.91510000
C	-0.76920000	-3.12650000	-3.03660000
H	-1.02330000	-3.07280000	-4.09560000
C	-0.42540000	-1.96770000	-2.35160000
H	-0.42850000	-0.99800000	-2.85530000
C	-0.86820000	2.38080000	-1.25600000
C	-0.56190000	2.62620000	-2.60010000
H	-1.04260000	2.02420000	-3.37270000
C	0.38530000	3.58750000	-2.93350000
H	0.62860000	3.76070000	-3.98190000
C	1.03670000	4.31390000	-1.93690000
H	1.78300000	5.06150000	-2.20380000
C	0.72290000	4.08350000	-0.59880000
H	1.22290000	4.64640000	0.19030000
C	-0.22640000	3.12670000	-0.26010000
H	-0.46980000	2.92810000	0.78530000
C	-2.88850000	1.11140000	-1.60850000
H	-3.26150000	1.84880000	-2.31800000
C	-3.48970000	-0.08730000	-1.30800000
H	-4.44910000	-0.41970000	-1.70270000
C	-3.22840000	-2.14570000	-0.10420000
C	-3.81340000	-2.96610000	-1.07830000
H	-3.93500000	-2.58940000	-2.09400000
C	-4.17540000	-4.27140000	-0.76840000
H	-4.61650000	-4.90080000	-1.54170000
C	-3.96210000	-4.78370000	0.50950000
H	-4.24760000	-5.80830000	0.74600000

C	-3.37440000	-3.97420000	1.47960000
H	-3.20050000	-4.36130000	2.48380000
C	-3.00930000	-2.66810000	1.17750000
H	-2.55580000	-2.02530000	1.93460000
C	-4.90030000	-0.03320000	2.90080000
C	-3.59010000	0.15520000	3.32580000
C	-2.81700000	1.20250000	2.81440000
C	-3.38980000	2.06940000	1.87770000
C	-4.70640000	1.88800000	1.45510000
C	-5.45970000	0.83350000	1.96230000
H	-5.48780000	-0.86050000	3.29870000
H	-3.14330000	-0.52200000	4.05770000
H	-2.79920000	2.89890000	1.48280000
H	-5.13980000	2.57400000	0.72740000
H	-6.48770000	0.68780000	1.63090000
C	-1.35560000	1.30940000	3.17590000
H	-1.02440000	2.36550000	3.10300000
H	-1.19840000	0.99090000	4.22260000
O	-0.58820000	0.49390000	2.32960000
H	0.66400000	0.30240000	1.42920000

Species J

HF energy= -1740.82580422

No Imaginary frequency

Zero-point correction= 0.586208 (Hartree/Particle)

Thermal correction to Energy= 0.621723

Thermal correction to Enthalpy= 0.622667

Thermal correction to Gibbs Free Energy= 0.518071

Sum of electronic and zero-point Energies= -1740.239596

Sum of electronic and thermal Energies= -1740.204081

Sum of electronic and thermal Enthalpies= -1740.203137

Sum of electronic and thermal Free Energies= -1740.307733

Coordinates: **J**

Ru	-1.32110000	0.08740000	0.52960000
N	0.23400000	-0.85710000	-0.25210000
N	1.94730000	1.13350000	1.17430000
N	-1.76090000	1.35750000	-0.88620000
N	-2.81760000	-0.83460000	-0.39700000
C	1.26220000	-0.11600000	-0.80600000
H	1.51090000	-0.29030000	-1.85710000
C	2.02530000	0.79890000	-0.14340000
H	2.77420000	1.33210000	-0.72630000
C	2.60650000	2.19630000	1.78580000
C	3.64480000	2.91940000	1.18300000
H	4.00200000	2.65780000	0.18790000
C	4.23500000	3.98230000	1.85890000
H	5.04230000	4.53150000	1.37360000

C	3.81590000	4.34610000	3.13580000
H	4.28630000	5.17970000	3.65520000
C	2.79050000	3.62020000	3.73990000
H	2.44990000	3.88310000	4.74150000
C	2.19050000	2.55970000	3.07620000
H	1.39110000	1.98670000	3.54960000
C	-0.10520000	-2.02740000	-0.98940000
C	-0.14480000	-3.25470000	-0.32760000
H	0.09560000	-3.27730000	0.73610000
C	-0.48570000	-4.41410000	-1.01900000
H	-0.51830000	-5.36650000	-0.49010000
C	-0.79460000	-4.35430000	-2.37400000
H	-1.06500000	-5.26090000	-2.91510000
C	-0.76920000	-3.12650000	-3.03660000
H	-1.02330000	-3.07280000	-4.09560000
C	-0.42540000	-1.96770000	-2.35160000
H	-0.42850000	-0.99800000	-2.85530000
C	-0.86820000	2.38080000	-1.25600000
C	-0.56190000	2.62620000	-2.60010000
H	-1.04260000	2.02420000	-3.37270000
C	0.38530000	3.58750000	-2.93350000
H	0.62860000	3.76070000	-3.98190000
C	1.03670000	4.31390000	-1.93690000
H	1.78300000	5.06150000	-2.20380000
C	0.72290000	4.08350000	-0.59880000
H	1.22290000	4.64640000	0.19030000
C	-0.22640000	3.12670000	-0.26010000
H	-0.46980000	2.92810000	0.78530000
C	-2.88850000	1.11140000	-1.60850000
H	-3.26150000	1.84880000	-2.31800000
C	-3.48970000	-0.08730000	-1.30800000
H	-4.44910000	-0.41970000	-1.70270000
C	-3.22840000	-2.14570000	-0.10420000
C	-3.81340000	-2.96610000	-1.07830000
H	-3.93500000	-2.58940000	-2.09400000
C	-4.17540000	-4.27140000	-0.76840000
H	-4.61650000	-4.90080000	-1.54170000
C	-3.96210000	-4.78370000	0.50950000
H	-4.24760000	-5.80830000	0.74600000
C	-3.37440000	-3.97420000	1.47960000
H	-3.20050000	-4.36130000	2.48380000
C	-3.00930000	-2.66810000	1.17750000
H	-2.55580000	-2.02530000	1.93460000
C	-4.90030000	-0.03320000	2.90080000
C	-3.59010000	0.15520000	3.32580000
C	-2.81700000	1.20250000	2.81440000
C	-3.38980000	2.06940000	1.87770000
C	-4.70640000	1.88800000	1.45510000

C	-5.45970000	0.83350000	1.96230000
H	-5.48780000	-0.86050000	3.29870000
H	-3.14330000	-0.52200000	4.05770000
H	-2.79920000	2.89890000	1.48280000
H	-5.13980000	2.57400000	0.72740000
H	-6.48770000	0.68780000	1.63090000
C	-1.35560000	1.30940000	3.17590000
H	-1.02440000	2.36550000	3.10300000
H	-1.19840000	0.99090000	4.22260000
O	-0.58820000	0.49390000	2.32960000
H	1.16710000	0.74200000	1.71410000

Species TSJK

HF energy= -1740.80244460

One imaginary frequency (368*i*)

Zero-point correction= 0.582779 (Hartree/Particle)

Thermal correction to Energy= 0.617515

Thermal correction to Enthalpy= 0.618460

Thermal correction to Gibbs Free Energy= 0.517358

Sum of electronic and zero-point Energies= -1740.219665

Sum of electronic and thermal Energies= -1740.184929

Sum of electronic and thermal Enthalpies= -1740.183985

Sum of electronic and thermal Free Energies= -1740.285087

Coordinates: **TSJK**

Ru	-1.36670000	0.72810000	1.75380000
N	0.30920000	0.01240000	0.84170000
N	2.33070000	1.57830000	2.01600000
N	-1.63350000	2.16710000	0.41400000
N	-2.77790000	-0.12330000	0.43130000
C	1.06140000	0.92710000	0.09800000
H	0.87050000	1.04500000	-0.97380000
C	1.96260000	1.73090000	0.69390000
H	2.46800000	2.52480000	0.14300000
C	2.65120000	2.61490000	2.88560000
C	2.42310000	3.95510000	2.54790000
H	1.95460000	4.20240000	1.59460000
C	2.73790000	4.96230000	3.45280000
H	2.53690000	5.99840000	3.17610000
C	3.27140000	4.66140000	4.70430000
H	3.51000000	5.45590000	5.41010000
C	3.49270000	3.32620000	5.04230000
H	3.90940000	3.07070000	6.01670000
C	3.19190000	2.31010000	4.14400000
H	3.37750000	1.26580000	4.40380000
C	0.09670000	-1.26900000	0.30780000
C	-0.33450000	-2.27490000	1.18980000
H	-0.46900000	-2.01890000	2.24290000

C	-0.57880000	-3.56630000	0.74220000
H	-0.92660000	-4.32070000	1.44800000
C	-0.38230000	-3.89250000	-0.59730000
H	-0.56830000	-4.90590000	-0.95200000
C	0.07100000	-2.91110000	-1.47760000
H	0.24410000	-3.15780000	-2.52590000
C	0.31240000	-1.61440000	-1.03760000
H	0.68080000	-0.87220000	-1.74400000
C	-1.06500000	3.46760000	0.49760000
C	-0.41610000	4.03720000	-0.60180000
H	-0.33140000	3.46490000	-1.52660000
C	0.13820000	5.31050000	-0.50080000
H	0.65130000	5.74170000	-1.36040000
C	0.04610000	6.02660000	0.69100000
H	0.48400000	7.02160000	0.76840000
C	-0.60560000	5.45850000	1.78450000
H	-0.68210000	6.00170000	2.72670000
C	-1.15860000	4.18690000	1.69110000
H	-1.67670000	3.74300000	2.54180000
C	-2.45250000	1.91110000	-0.61600000
H	-2.70080000	2.69580000	-1.33080000
C	-3.05270000	0.65140000	-0.61200000
H	-3.77390000	0.34560000	-1.37290000
C	-3.34550000	-1.41240000	0.49430000
C	-3.53210000	-2.19830000	-0.64980000
H	-3.17240000	-1.83820000	-1.61440000
C	-4.09850000	-3.46310000	-0.54180000
H	-4.22310000	-4.07290000	-1.43670000
C	-4.48650000	-3.95730000	0.70090000
H	-4.93100000	-4.94890000	0.78230000
C	-4.29150000	-3.18000000	1.84230000
H	-4.58830000	-3.56040000	2.81970000
C	-3.71950000	-1.91830000	1.74560000
H	-3.55110000	-1.30520000	2.63110000
C	-0.67850000	4.24020000	5.65340000
C	-0.31400000	3.04560000	5.04300000
C	-1.29380000	2.15820000	4.59340000
C	-2.64310000	2.46580000	4.77860000
C	-3.00630000	3.66000000	5.39540000
C	-2.02650000	4.55220000	5.82730000
H	0.09700000	4.92780000	5.99210000
H	0.74220000	2.80510000	4.89650000
H	-3.39830000	1.76010000	4.43250000
H	-4.06070000	3.89480000	5.54110000
H	-2.31360000	5.48780000	6.30730000
C	-0.89370000	0.91280000	3.87310000
H	-0.26300000	1.66010000	2.54530000
H	0.10460000	0.52650000	4.16090000

O	-1.81640000	0.00340000	3.60700000
H	2.04540000	0.69640000	2.42870000

Species K
HF energy= -1740.80628016
No Imaginary frequency
Zero-point correction= 0.583302 (Hartree/Particle)
Thermal correction to Energy= 0.618720
Thermal correction to Enthalpy= 0.619664
Thermal correction to Gibbs Free Energy= 0.516650
Sum of electronic and zero-point Energies= -1740.222979
Sum of electronic and thermal Energies= -1740.187560
Sum of electronic and thermal Enthalpies= -1740.186616
Sum of electronic and thermal Free Energies= -1740.289630

Coordinates: **K**

Ru	-1.11400000	0.88750000	1.88540000
N	0.41370000	-0.00420000	0.85900000
N	2.48800000	1.54750000	2.00200000
N	-1.46140000	2.27210000	0.48650000
N	-2.65980000	-0.04300000	0.59330000
C	1.19030000	0.87950000	0.10420000
H	1.02860000	0.97220000	-0.97430000
C	2.12470000	1.66880000	0.67460000
H	2.65530000	2.42660000	0.09740000
C	2.71840000	2.61430000	2.86770000
C	2.49010000	3.94340000	2.48820000
H	2.08880000	4.16680000	1.49850000
C	2.72230000	4.97330000	3.39350000
H	2.53270000	6.00160000	3.08210000
C	3.16600000	4.70770000	4.68700000
H	3.33910000	5.52010000	5.39150000
C	3.38450000	3.38350000	5.06770000
H	3.73200000	3.15360000	6.07510000
C	3.16910000	2.34600000	4.16970000
H	3.35580000	1.31060000	4.46270000
C	0.08570000	-1.24770000	0.28170000
C	-0.31160000	-2.28700000	1.14040000
H	-0.37780000	-2.07520000	2.20740000
C	-0.62380000	-3.54510000	0.64390000
H	-0.93460000	-4.32900000	1.33510000
C	-0.55340000	-3.80420000	-0.72330000
H	-0.79920000	-4.79150000	-1.11360000
C	-0.15530000	-2.78480000	-1.58510000
H	-0.08670000	-2.97170000	-2.65760000
C	0.16550000	-1.52320000	-1.09470000
H	0.48520000	-0.75440000	-1.79610000
C	-0.92760000	3.59010000	0.52520000

C	-0.24700000	4.12440000	-0.57020000
H	-0.10980000	3.51550000	-1.46480000
C	0.28160000	5.41130000	-0.49540000
H	0.82500000	5.81840000	-1.34780000
C	0.12590000	6.17010000	0.66210000
H	0.54420000	7.17490000	0.71810000
C	-0.56250000	5.63430000	1.75050000
H	-0.68930000	6.21240000	2.66630000
C	-1.08600000	4.35010000	1.68570000
H	-1.62260000	3.92030000	2.53230000
C	-2.25400000	1.95350000	-0.53130000
H	-2.48250000	2.69130000	-1.30220000
C	-2.89510000	0.70010000	-0.46570000
H	-3.62700000	0.40630000	-1.22360000
C	-3.32300000	-1.27900000	0.72180000
C	-3.64890000	-2.06940000	-0.38870000
H	-3.32370000	-1.76380000	-1.38390000
C	-4.30740000	-3.28040000	-0.21370000
H	-4.54000000	-3.89400000	-1.08420000
C	-4.64960000	-3.71880000	1.06310000
H	-5.16430000	-4.66990000	1.19670000
C	-4.30880000	-2.94250000	2.17020000
H	-4.55790000	-3.28390000	3.17480000
C	-3.64260000	-1.73570000	2.00650000
H	-3.33560000	-1.13900000	2.86500000
C	-1.09890000	4.04930000	5.38590000
C	-0.46880000	2.89190000	4.95910000
C	-1.22600000	1.79950000	4.50870000
C	-2.62520000	1.87850000	4.51620000
C	-3.25350000	3.05020000	4.94240000
C	-2.49600000	4.13550000	5.37090000
H	-0.49870000	4.89430000	5.72530000
H	0.62240000	2.82590000	4.94030000
H	-3.21000000	1.01120000	4.20800000
H	-4.34180000	3.10760000	4.94750000
H	-2.99000000	5.04740000	5.70590000
C	-0.54710000	0.63220000	3.89580000
H	0.02310000	1.90960000	2.30450000
H	0.53650000	0.53960000	4.09520000
O	-1.22260000	-0.42110000	3.55550000
H	2.15840000	0.68720000	2.42940000

Species TSKL
HF energy= -1740.78667791
One imaginary frequency (171*i*)
Zero-point correction= 0.580427 (Hartree/Particle)
Thermal correction to Energy= 0.616336
Thermal correction to Enthalpy= 0.617281

Thermal correction to Gibbs Free Energy= 0.510867
Sum of electronic and zero-point Energies= -1740.206251
Sum of electronic and thermal Energies= -1740.170341
Sum of electronic and thermal Enthalpies= -1740.169397
Sum of electronic and thermal Free Energies= -1740.275811

Coordinates: **TSKL**

Ru	-0.71040000	0.34340000	2.03960000
N	0.58610000	-0.11820000	0.63130000
N	2.38030000	2.10130000	1.62490000
N	-1.26130000	2.07530000	1.30030000
N	-2.45840000	-0.20490000	0.89970000
C	1.47800000	0.64610000	-0.10110000
H	1.61860000	0.34830000	-1.14270000
C	2.27480000	1.65650000	0.33770000
H	2.92390000	2.13550000	-0.39260000
C	3.08070000	3.20870000	2.07410000
C	3.75290000	4.09670000	1.22220000
H	3.72960000	3.95090000	0.14280000
C	4.42620000	5.19380000	1.74800000
H	4.94010000	5.87320000	1.06690000
C	4.44610000	5.43780000	3.11890000
H	4.97700000	6.29990000	3.52060000
C	3.77770000	4.55550000	3.96770000
H	3.78200000	4.72410000	5.04520000
C	3.10590000	3.45540000	3.45680000
H	2.58350000	2.76410000	4.12210000
C	0.15860000	-1.31740000	0.01640000
C	0.08730000	-2.47160000	0.80620000
H	0.44050000	-2.41320000	1.83960000
C	-0.40360000	-3.66400000	0.28100000
H	-0.46990000	-4.54700000	0.91630000
C	-0.80430000	-3.72550000	-1.04910000
H	-1.18980000	-4.65650000	-1.46370000
C	-0.72360000	-2.58300000	-1.84700000
H	-1.05370000	-2.62160000	-2.88570000
C	-0.25070000	-1.38720000	-1.32360000
H	-0.23750000	-0.48320000	-1.93430000
C	-0.60820000	3.32260000	1.49520000
C	-0.15950000	4.07160000	0.40140000
H	-0.26370000	3.65730000	-0.60200000
C	0.45780000	5.30170000	0.60200000
H	0.82630000	5.86440000	-0.25590000
C	0.62520000	5.80400000	1.89010000
H	1.12830000	6.75770000	2.04660000
C	0.18190000	5.05780000	2.97920000
H	0.32280000	5.43320000	3.99310000
C	-0.42520000	3.82240000	2.78620000

H	-0.76320000	3.22590000	3.63270000
C	-2.20720000	2.02950000	0.33450000
H	-2.48200000	2.94330000	-0.19190000
C	-2.87170000	0.82200000	0.15470000
H	-3.74940000	0.74770000	-0.49320000
C	-3.15650000	-1.42180000	0.82910000
C	-3.74080000	-1.88360000	-0.36030000
H	-3.62260000	-1.30400000	-1.27650000
C	-4.41270000	-3.10050000	-0.39240000
H	-4.84960000	-3.44450000	-1.33060000
C	-4.51500000	-3.88350000	0.75410000
H	-5.04180000	-4.83690000	0.72560000
C	-3.92870000	-3.43540000	1.93710000
H	-3.99940000	-4.03750000	2.84350000
C	-3.25250000	-2.22300000	1.97700000
H	-2.79360000	-1.87100000	2.90050000
C	-2.94070000	3.77550000	6.01440000
C	-2.20000000	2.59990000	5.98890000
C	-2.38550000	1.67800000	4.95220000
C	-3.34040000	1.92130000	3.95220000
C	-4.08330000	3.09010000	3.98800000
C	-3.87790000	4.01900000	5.01230000
H	-2.79040000	4.50110000	6.81250000
H	-1.45860000	2.39300000	6.76320000
H	-3.47050000	1.19250000	3.15000000
H	-4.82070000	3.28770000	3.21110000
H	-4.45710000	4.94180000	5.02770000
C	-1.55450000	0.48260000	4.91780000
H	0.48400000	0.96160000	2.99760000
H	-0.90340000	0.31600000	5.80380000
O	-1.54710000	-0.34500000	4.00650000
H	1.75230000	1.64240000	2.29080000

Species L
HF energy= -1740.79154422
No Imaginary frequency
Zero-point correction= 0.582004 (Hartree/Particle)
Thermal correction to Energy= 0.618026
Thermal correction to Enthalpy= 0.618970
Thermal correction to Gibbs Free Energy= 0.512680
Sum of electronic and zero-point Energies= -1740.209540
Sum of electronic and thermal Energies= -1740.173518
Sum of electronic and thermal Enthalpies= -1740.172574
Sum of electronic and thermal Free Energies= -1740.278864

Coordinates: **L**

Ru	-1.45450000	-0.28080000	0.30160000
N	0.26800000	-1.17920000	-0.11040000

N	1.66940000	1.34160000	0.94000000
N	-1.29950000	1.05450000	-1.15220000
N	-2.50780000	-1.25850000	-1.35680000
C	1.43530000	-0.58410000	-0.54730000
H	1.98050000	-1.12730000	-1.32340000
C	2.06190000	0.53550000	-0.08930000
H	2.99380000	0.80870000	-0.58060000
C	2.22020000	2.56260000	1.29890000
C	3.26930000	3.17270000	0.59730000
H	3.68300000	2.70550000	-0.29530000
C	3.76760000	4.40160000	1.01540000
H	4.58330000	4.85890000	0.45430000
C	3.23570000	5.05500000	2.12400000
H	3.63230000	6.01780000	2.44360000
C	2.18730000	4.45280000	2.81930000
H	1.75400000	4.94500000	3.69070000
C	1.68670000	3.22350000	2.41770000
H	0.86530000	2.75220000	2.96240000
C	0.07250000	-2.48950000	-0.62850000
C	-0.25240000	-3.52750000	0.24920000
H	-0.34740000	-3.29570000	1.30910000
C	-0.44480000	-4.82150000	-0.22620000
H	-0.70020000	-5.61870000	0.47210000
C	-0.31410000	-5.09860000	-1.58370000
H	-0.47050000	-6.11040000	-1.95690000
C	0.00900000	-4.06820000	-2.46590000
H	0.10020000	-4.27200000	-3.53360000
C	0.19930000	-2.77500000	-1.99590000
H	0.41120000	-1.95970000	-2.69000000
C	-0.70810000	2.34110000	-1.04530000
C	0.24540000	2.77050000	-1.97560000
H	0.56720000	2.08090000	-2.75700000
C	0.81800000	4.03200000	-1.86090000
H	1.58190000	4.34240000	-2.57420000
C	0.43720000	4.88800000	-0.83000000
H	0.89740000	5.87050000	-0.73210000
C	-0.51780000	4.46660000	0.09140000
H	-0.81380000	5.12330000	0.90960000
C	-1.08340000	3.20010000	-0.00890000
H	-1.81850000	2.85330000	0.71790000
C	-1.71240000	0.67500000	-2.37790000
H	-1.64200000	1.36980000	-3.21540000
C	-2.37510000	-0.54460000	-2.47350000
H	-2.84700000	-0.85170000	-3.41070000
C	-3.20840000	-2.47240000	-1.40680000
C	-3.26570000	-3.27160000	-2.56070000
H	-2.71810000	-2.97130000	-3.45380000
C	-3.96750000	-4.47070000	-2.55940000

H	-3.98670000	-5.07780000	-3.46510000
C	-4.62480000	-4.90830000	-1.41270000
H	-5.17180000	-5.85050000	-1.41540000
C	-4.56780000	-4.12760000	-0.25970000
H	-5.07680000	-4.45450000	0.64750000
C	-3.86490000	-2.93050000	-0.25470000
H	-3.81720000	-2.32320000	0.64750000
C	-4.95850000	1.46220000	4.17790000
C	-4.17050000	0.31980000	4.13940000
C	-3.59280000	-0.09590000	2.93420000
C	-3.84550000	0.62510000	1.75660000
C	-4.64200000	1.76140000	1.79450000
C	-5.18810000	2.18440000	3.00660000
H	-5.39450000	1.79420000	5.11900000
H	-3.97520000	-0.25140000	5.04880000
H	-3.51150000	0.24540000	0.77480000
H	-4.84260000	2.31240000	0.87640000
H	-5.80820000	3.07980000	3.03560000
C	-2.69310000	-1.23860000	2.93240000
H	-0.77920000	0.70090000	1.43450000
H	-2.68680000	-1.87770000	3.83600000
O	-1.92800000	-1.51720000	2.01190000
H	0.76710000	1.09710000	1.35570000

Species TSLM
HF energy= -1740.76467282
One imaginary frequency (1631*i*)
Zero-point correction= 0.574585 (Hartree/Particle)
Thermal correction to Energy= 0.610754
Thermal correction to Enthalpy= 0.611698
Thermal correction to Gibbs Free Energy= 0.502145
Sum of electronic and zero-point Energies= -1740.190088
Sum of electronic and thermal Energies= -1740.153919
Sum of electronic and thermal Enthalpies= -1740.152975
Sum of electronic and thermal Free Energies= -1740.262527

Coordinates: **TSLM**

Ru	-1.39270000	-0.10150000	0.05550000
N	0.30330000	-1.10850000	-0.27970000
N	1.48560000	1.36560000	0.85520000
N	-1.24590000	1.15130000	-1.47340000
N	-2.55520000	-1.04200000	-1.36390000
C	1.52980000	-0.61160000	-0.52220000
H	2.18990000	-1.20890000	-1.15640000
C	2.09720000	0.54810000	0.03420000
H	3.14260000	0.73400000	-0.24840000
C	2.04950000	2.53030000	1.35600000
C	3.16280000	3.17650000	0.79340000

H	3.63980000	2.76480000	-0.09620000
C	3.62630000	4.37140000	1.32980000
H	4.48550000	4.86330000	0.87220000
C	2.99800000	4.95110000	2.43030000
H	3.36540000	5.89050000	2.84210000
C	1.89100000	4.31670000	2.99380000
H	1.38810000	4.75820000	3.85470000
C	1.42050000	3.12400000	2.46360000
H	0.55440000	2.62350000	2.89920000
C	0.09770000	-2.41080000	-0.83360000
C	-0.35630000	-3.43600000	-0.00150000
H	-0.59100000	-3.20040000	1.03510000
C	-0.50110000	-4.72590000	-0.50050000
H	-0.85920000	-5.51830000	0.15680000
C	-0.20270000	-5.00310000	-1.83220000
H	-0.31940000	-6.01420000	-2.22200000
C	0.22450000	-3.97520000	-2.67090000
H	0.43910000	-4.17880000	-3.72040000
C	0.37390000	-2.68290000	-2.17780000
H	0.68710000	-1.86550000	-2.82950000
C	-0.50650000	2.35610000	-1.48830000
C	0.45920000	2.60320000	-2.47310000
H	0.65640000	1.83670000	-3.22440000
C	1.19230000	3.78450000	-2.46130000
H	1.95190000	3.95150000	-3.22570000
C	0.97540000	4.73960000	-1.47010000
H	1.56360000	5.65670000	-1.45020000
C	0.01660000	4.50010000	-0.48930000
H	-0.14670000	5.23000000	0.30410000
C	-0.72060000	3.32170000	-0.49900000
H	-1.47290000	3.12530000	0.26650000
C	-1.69100000	0.66470000	-2.66550000
H	-1.58120000	1.25470000	-3.57490000
C	-2.40740000	-0.50690000	-2.59780000
H	-2.91710000	-0.93930000	-3.46000000
C	-3.25410000	-2.25310000	-1.23180000
C	-3.21930000	-3.24860000	-2.21960000
H	-2.61130000	-3.09790000	-3.11120000
C	-3.88970000	-4.45270000	-2.03780000
H	-3.82470000	-5.21910000	-2.81130000
C	-4.61420000	-4.69420000	-0.87380000
H	-5.13360000	-5.64160000	-0.73220000
C	-4.67190000	-3.70270000	0.10440000
H	-5.25480000	-3.86450000	1.01190000
C	-4.00690000	-2.49700000	-0.07390000
H	-4.08200000	-1.70870000	0.67380000
C	-5.27680000	0.81300000	4.53840000
C	-4.27250000	-0.11090000	4.28630000

C	-3.60240000	-0.11210000	3.05510000
C	-3.95900000	0.82370000	2.07200000
C	-4.96730000	1.74280000	2.32410000
C	-5.62270000	1.74020000	3.55620000
H	-5.79320000	0.81240000	5.49730000
H	-3.99460000	-0.84540000	5.04470000
H	-3.44420000	0.82250000	1.10340000
H	-5.24440000	2.46480000	1.55730000
H	-6.41080000	2.46730000	3.75060000
C	-2.56120000	-1.11210000	2.84850000
H	-0.80700000	1.01760000	1.28940000
H	-2.40490000	-1.81280000	3.69430000
O	-1.86330000	-1.25790000	1.85190000
H	0.09080000	1.04470000	1.05520000

Species M

HF energy= -1740.77376415

No Imaginary frequency

Zero-point correction= 0.578350 (Hartree/Particle)

Thermal correction to Energy= 0.614862

Thermal correction to Enthalpy= 0.615806

Thermal correction to Gibbs Free Energy= 0.508097

Sum of electronic and zero-point Energies= -1740.195414

Sum of electronic and thermal Energies= -1740.158902

Sum of electronic and thermal Enthalpies= -1740.157958

Sum of electronic and thermal Free Energies= -1740.265667

Coordinates: **M**

Ru	-1.49450000	-0.00190000	0.02070000
N	0.34960000	-0.91310000	-0.16540000
N	1.55150000	1.56100000	1.09110000
N	-1.27550000	1.13640000	-1.62360000
N	-2.73320000	-0.91920000	-1.27990000
C	1.58230000	-0.45260000	-0.25860000
H	2.28710000	-1.05480000	-0.84460000
C	2.16160000	0.72880000	0.32000000
H	3.22320000	0.86560000	0.04760000
C	2.16560000	2.72840000	1.53530000
C	3.25780000	3.34590000	0.90300000
H	3.68220000	2.90690000	-0.00010000
C	3.75480000	4.55250000	1.37840000
H	4.59230000	5.02710000	0.86610000
C	3.17870000	5.16910000	2.48840000
H	3.56840000	6.11960000	2.85200000
C	2.08970000	4.56830000	3.11850000
H	1.62470000	5.04660000	3.98070000
C	1.58390000	3.36630000	2.64200000
H	0.72380000	2.88740000	3.11250000

C	0.19260000	-2.17630000	-0.82190000
C	-0.07900000	-3.31690000	-0.06420000
H	-0.22640000	-3.21010000	1.00990000
C	-0.14480000	-4.55930000	-0.68660000
H	-0.34740000	-5.44860000	-0.08990000
C	0.04060000	-4.66950000	-2.06380000
H	-0.01530000	-5.64460000	-2.54720000
C	0.28450000	-3.52580000	-2.82090000
H	0.41820000	-3.60210000	-3.90010000
C	0.36730000	-2.27980000	-2.20500000
H	0.55070000	-1.37240000	-2.78320000
C	-0.35370000	2.19480000	-1.76270000
C	0.57110000	2.22120000	-2.81770000
H	0.57610000	1.40400000	-3.54020000
C	1.50530000	3.24630000	-2.91850000
H	2.22360000	3.23680000	-3.73900000
C	1.53830000	4.26710000	-1.97090000
H	2.27970000	5.06310000	-2.04080000
C	0.62030000	4.25190000	-0.92280000
H	0.64750000	5.03450000	-0.16290000
C	-0.31760000	3.23250000	-0.82120000
H	-1.03870000	3.22230000	-0.00370000
C	-1.80490000	0.59420000	-2.75340000
H	-1.66690000	1.08600000	-3.71640000
C	-2.59420000	-0.51450000	-2.57100000
H	-3.14680000	-1.01060000	-3.36990000
C	-3.45150000	-2.10850000	-1.03500000
C	-3.20100000	-3.27370000	-1.76830000
H	-2.43000000	-3.25450000	-2.53940000
C	-3.89120000	-4.44720000	-1.48420000
H	-3.66660000	-5.35000000	-2.05400000
C	-4.85080000	-4.47610000	-0.47500000
H	-5.39060000	-5.39680000	-0.25450000
C	-5.12120000	-3.31250000	0.24380000
H	-5.88700000	-3.31360000	1.02070000
C	-4.43100000	-2.13860000	-0.03590000
H	-4.66180000	-1.21590000	0.49720000
C	-5.70760000	0.11150000	4.47540000
C	-4.62730000	-0.69350000	4.14040000
C	-3.84960000	-0.40120000	3.01190000
C	-4.16720000	0.71160000	2.21850000
C	-5.24840000	1.51320000	2.55560000
C	-6.01690000	1.21520000	3.68160000
H	-6.31070000	-0.11990000	5.35240000
H	-4.37520000	-1.56280000	4.75100000
H	-3.56050000	0.92790000	1.33200000
H	-5.49740000	2.37440000	1.93680000
H	-6.86580000	1.84730000	3.94150000

C	-2.73770000	-1.29420000	2.70020000
H	-1.20980000	1.35080000	1.15420000
H	-2.59940000	-2.13250000	3.41440000
O	-1.95730000	-1.21950000	1.75890000
H	-0.43620000	1.04810000	1.11760000

Species TSMEN
HF energy= -1740.76508731
One imaginary frequency (232*i*)
Zero-point correction= 0.578219 (Hartree/Particle)
Thermal correction to Energy= 0.614529
Thermal correction to Enthalpy= 0.615473
Thermal correction to Gibbs Free Energy= 0.508369
Sum of electronic and zero-point Energies= -1740.186869
Sum of electronic and thermal Energies= -1740.150559
Sum of electronic and thermal Enthalpies= -1740.149615
Sum of electronic and thermal Free Energies= -1740.256719

Coordinates: **TSMEN**

Ru	-1.38290000	-0.09890000	-0.00960000
N	0.47480000	-0.91000000	-0.18820000
N	1.67120000	1.59900000	0.96070000
N	-1.16480000	1.09250000	-1.62680000
N	-2.74630000	-0.79470000	-1.22360000
C	1.67900000	-0.40420000	-0.40430000
H	2.36040000	-0.99950000	-1.02360000
C	2.24880000	0.79510000	0.13300000
H	3.28760000	0.96990000	-0.20050000
C	2.31490000	2.75910000	1.38380000
C	3.27950000	3.44410000	0.62610000
H	3.55110000	3.07160000	-0.36190000
C	3.82690000	4.63280000	1.09110000
H	4.56270000	5.15900000	0.48200000
C	3.42900000	5.16530000	2.31650000
H	3.85700000	6.10120000	2.67460000
C	2.46480000	4.49850000	3.07150000
H	2.13850000	4.90900000	4.02730000
C	1.90740000	3.31440000	2.60670000
H	1.14920000	2.78480000	3.18500000
C	0.32430000	-2.22400000	-0.73470000
C	0.22220000	-3.32190000	0.12090000
H	0.22410000	-3.15020000	1.19610000
C	0.11630000	-4.60340000	-0.40950000
H	0.04000000	-5.45800000	0.26290000
C	0.11460000	-4.79920000	-1.79010000
H	0.03340000	-5.80490000	-2.20190000
C	0.21790000	-3.70030000	-2.64190000
H	0.21360000	-3.84320000	-3.72260000

C	0.32570000	-2.41430000	-2.12000000
H	0.38660000	-1.53810000	-2.76890000
C	-0.33890000	2.22870000	-1.73210000
C	0.52050000	2.40790000	-2.82460000
H	0.57100000	1.63570000	-3.59410000
C	1.33400000	3.53350000	-2.90460000
H	2.00460000	3.65050000	-3.75650000
C	1.30560000	4.49790000	-1.89950000
H	1.95310000	5.37310000	-1.95390000
C	0.46160000	4.31840000	-0.80510000
H	0.46010000	5.04710000	0.00710000
C	-0.35170000	3.19540000	-0.71900000
H	-1.01240000	3.04460000	0.13530000
C	-1.79790000	0.64530000	-2.73520000
H	-1.68890000	1.16470000	-3.68770000
C	-2.65390000	-0.41350000	-2.52770000
H	-3.26900000	-0.89130000	-3.28930000
C	-3.55730000	-1.91720000	-0.90710000
C	-3.14510000	-3.20780000	-1.24730000
H	-2.20920000	-3.33440000	-1.79180000
C	-3.90820000	-4.30560000	-0.86600000
H	-3.56670000	-5.30910000	-1.12330000
C	-5.09620000	-4.12590000	-0.15870000
H	-5.69320000	-4.98830000	0.13770000
C	-5.52270000	-2.83750000	0.15560000
H	-6.45800000	-2.68450000	0.69520000
C	-4.75660000	-1.73540000	-0.21590000
H	-5.07380000	-0.72110000	0.03050000
C	-6.00860000	-0.39190000	3.89280000
C	-4.89150000	-1.16780000	3.61610000
C	-3.89230000	-0.69170000	2.75670000
C	-4.01230000	0.58810000	2.19570000
C	-5.12680000	1.36490000	2.48010000
C	-6.12590000	0.87500000	3.32220000
H	-6.78780000	-0.77030000	4.55300000
H	-4.78990000	-2.16480000	4.04940000
H	-3.23610000	0.95630000	1.51750000
H	-5.22150000	2.35690000	2.04020000
H	-7.00040000	1.48780000	3.54000000
C	-2.78060000	-1.58760000	2.45650000
H	-1.03250000	1.12950000	1.81240000
H	-2.75900000	-2.52130000	3.05600000
O	-1.89390000	-1.43060000	1.62420000
H	-0.27460000	1.24670000	1.71320000

Species N
HF energy= -1.16669301727
No Imaginary frequency

Zero-point correction= 0.009967 (Hartree/Particle)
Thermal correction to Energy= 0.012327
Thermal correction to Enthalpy= 0.013272
Thermal correction to Gibbs Free Energy= -0.001518
Sum of electronic and zero-point Energies= -1.156726
Sum of electronic and thermal Energies= -1.154366
Sum of electronic and thermal Enthalpies= -1.153421
Sum of electronic and thermal Free Energies= -1.168211

Coordinates: **N**

H	0.81940000	-0.23870000	0.00000000
H	0.07740000	-0.23870000	0.00000000

Species TSBEO
HF energy= -2086.13350409
One imaginary frequency (856*i*)
Zero-point correction= 0.694739 (Hartree/Particle)
Thermal correction to Energy= 0.736039
Thermal correction to Enthalpy= 0.736983
Thermal correction to Gibbs Free Energy= 0.621131
Sum of electronic and zero-point Energies= -2085.438765
Sum of electronic and thermal Energies= -2085.397465
Sum of electronic and thermal Enthalpies= -2085.396521
Sum of electronic and thermal Free Energies= -2085.512373

Coordinates: **TSBEO**

Ru	-0.69330000	0.32730000	0.29420000
N	0.13580000	-0.38910000	-1.34210000
N	0.90410000	1.63040000	0.00960000
N	-2.35320000	1.62870000	0.59350000
N	-2.27420000	-0.96690000	0.37640000
C	1.29060000	0.19290000	-1.76590000
H	1.80570000	-0.18850000	-2.64740000
C	1.69840000	1.27790000	-1.02830000
H	2.61120000	1.83060000	-1.25170000
C	1.20850000	2.85850000	0.64450000
C	1.78600000	3.92560000	-0.06520000
H	1.94300000	3.83700000	-1.14030000
C	2.11820000	5.11170000	0.57810000
H	2.56370000	5.92480000	0.00390000
C	1.86700000	5.27130000	1.93850000
H	2.12420000	6.20290000	2.44200000
C	1.26120000	4.23190000	2.64060000
H	1.03400000	4.35040000	3.70080000
C	0.92850000	3.03960000	2.00720000
H	0.42220000	2.23460000	2.54130000
C	-0.37930000	-1.39880000	-2.20240000
C	0.24470000	-2.64280000	-2.29220000

H	1.10680000	-2.84600000	-1.65370000
C	-0.24540000	-3.60450000	-3.17070000
H	0.23980000	-4.57890000	-3.22900000
C	-1.35110000	-3.32550000	-3.97050000
H	-1.73040000	-4.07780000	-4.66160000
C	-1.97650000	-2.08370000	-3.87550000
H	-2.84370000	-1.85870000	-4.49680000
C	-1.49590000	-1.12180000	-2.99270000
H	-1.97080000	-0.14390000	-2.89600000
C	-2.29350000	3.01460000	0.83330000
C	-1.72850000	3.84640000	-0.13820000
H	-1.35460000	3.39320000	-1.05770000
C	-1.65020000	5.21530000	0.07990000
H	-1.21080000	5.85790000	-0.68300000
C	-2.10990000	5.76440000	1.27640000
H	-2.03310000	6.83730000	1.45040000
C	-2.65400000	4.93490000	2.25360000
H	-2.99900000	5.35510000	3.19850000
C	-2.74950000	3.56460000	2.03620000
H	-3.13590000	2.89960000	2.80990000
C	-3.45180000	0.97400000	0.90980000
H	-4.34680000	1.50120000	1.25440000
C	-3.43140000	-0.42230000	0.74470000
H	-4.30920000	-1.04450000	0.93190000
C	-2.30650000	-2.37120000	0.15540000
C	-3.30420000	-2.93470000	-0.64790000
H	-4.00690000	-2.27530000	-1.15920000
C	-3.36340000	-4.31150000	-0.83340000
H	-4.13360000	-4.73300000	-1.47920000
C	-2.43600000	-5.14330000	-0.21190000
H	-2.48670000	-6.22280000	-0.35240000
C	-1.44240000	-4.58460000	0.58810000
H	-0.71700000	-5.22490000	1.09020000
C	-1.36620000	-3.20780000	0.76310000
H	-0.59830000	-2.78010000	1.40380000
C	-3.01910000	-2.43710000	4.28500000
C	-2.23490000	-1.41690000	3.75190000
C	-0.84720000	-1.52380000	3.75280000
C	-0.24450000	-2.64110000	4.33520000
C	-1.02530000	-3.65920000	4.87020000
C	-2.41630000	-3.56350000	4.83690000
H	-4.10600000	-2.35160000	4.26860000
H	-2.68030000	-0.52460000	3.31250000
H	0.84480000	-2.71630000	4.35130000
H	-0.54860000	-4.53390000	5.31340000
H	-3.02840000	-4.36520000	5.25010000
C	-0.01480000	-0.41580000	3.14630000
H	0.69680000	-0.00170000	3.89550000

O	-0.65130000	0.50500000	2.45320000
C	4.25150000	-2.42780000	4.71360000
C	3.47960000	-2.16830000	3.58710000
C	3.11380000	-0.85730000	3.26690000
C	3.53710000	0.18880000	4.08700000
C	4.31660000	-0.06850000	5.21250000
C	4.67210000	-1.37620000	5.52760000
H	4.53150000	-3.45260000	4.95670000
H	3.14620000	-2.98570000	2.94400000
H	3.24780000	1.21230000	3.83680000
H	4.64220000	0.75580000	5.84630000
H	5.27910000	-1.58010000	6.40940000
C	2.27510000	-0.57670000	2.06140000
H	2.15880000	0.50320000	1.88380000
H	2.69550000	-1.02490000	1.14850000
O	0.96390000	-1.17510000	2.18390000
H	0.36700000	-0.89320000	1.18320000

Species O

HF energy= -2086.17353184

No Imaginary frequency

Zero-point correction= 0.698131 (Hartree/Particle)

Thermal correction to Energy= 0.739733

Thermal correction to Enthalpy= 0.740677

Thermal correction to Gibbs Free Energy= 0.623418

Sum of electronic and zero-point Energies= -2085.475401

Sum of electronic and thermal Energies= -2085.433799

Sum of electronic and thermal Enthalpies= -2085.432855

Sum of electronic and thermal Free Energies= -2085.550114

Coordinates: **O**

Ru	-0.59610000	0.48820000	0.12270000
N	-0.14720000	0.72100000	-1.80070000
N	0.52670000	2.20390000	0.15110000
N	-2.44430000	1.17150000	-0.22630000
N	-1.66080000	-1.25870000	-0.09360000
C	0.29870000	1.95760000	-2.12900000
H	0.45170000	2.22700000	-3.17410000
C	0.68510000	2.75450000	-1.06790000
H	1.18770000	3.71500000	-1.19140000
C	1.00010000	2.90630000	1.27670000
C	0.77940000	4.28250000	1.42780000
H	0.20160000	4.81260000	0.66960000
C	1.25990000	4.95460000	2.54740000
H	1.07530000	6.02440000	2.64950000
C	1.96630000	4.27040000	3.53300000
H	2.34500000	4.79910000	4.40720000
C	2.18730000	2.90140000	3.38750000

H	2.75060000	2.35660000	4.14580000
C	1.71050000	2.22340000	2.27270000
H	1.88950000	1.15630000	2.14450000
C	-0.56010000	-0.13960000	-2.84380000
C	-0.22110000	-1.49540000	-2.80950000
H	0.40200000	-1.86010000	-1.99450000
C	-0.68120000	-2.35890000	-3.79670000
H	-0.41190000	-3.41430000	-3.75200000
C	-1.47140000	-1.88210000	-4.84040000
H	-1.83260000	-2.56170000	-5.61190000
C	-1.79050000	-0.52670000	-4.89340000
H	-2.41090000	-0.14180000	-5.70320000
C	-1.33800000	0.33980000	-3.90510000
H	-1.61710000	1.39470000	-3.92090000
C	-2.73810000	2.54620000	-0.33690000
C	-3.26710000	3.11080000	-1.50450000
H	-3.42960000	2.47840000	-2.37920000
C	-3.53740000	4.47390000	-1.56170000
H	-3.93870000	4.90400000	-2.47960000
C	-3.27790000	5.29160000	-0.46380000
H	-3.48730000	6.35970000	-0.51460000
C	-2.74390000	4.73400000	0.69710000
H	-2.53030000	5.36340000	1.56170000
C	-2.47570000	3.37260000	0.76180000
H	-2.04260000	2.92710000	1.65940000
C	-3.23120000	0.25550000	-0.85470000
H	-4.13660000	0.56250000	-1.37920000
C	-2.85260000	-1.06440000	-0.69630000
H	-3.45840000	-1.90350000	-1.04470000
C	-1.32940000	-2.48820000	0.47480000
C	-2.29210000	-3.43150000	0.87460000
H	-3.35140000	-3.20520000	0.75060000
C	-1.90380000	-4.61880000	1.48240000
H	-2.66650000	-5.33430000	1.79120000
C	-0.55740000	-4.88960000	1.72230000
H	-0.26040000	-5.82120000	2.20300000
C	0.40370000	-3.95510000	1.34100000
H	1.46090000	-4.15410000	1.51820000
C	0.02540000	-2.77490000	0.71380000
H	0.77300000	-2.04500000	0.39170000
C	-3.44380000	-1.51790000	4.33650000
C	-2.44500000	-0.94090000	3.56590000
C	-1.10280000	-1.10130000	3.93960000
C	-0.77930000	-1.82910000	5.09280000
C	-1.78240000	-2.41290000	5.85390000
C	-3.11510000	-2.25720000	5.47390000
H	-4.48680000	-1.39350000	4.04590000
H	-2.68690000	-0.36370000	2.67290000

H	0.26840000	-1.94050000	5.37850000
H	-1.52910000	-2.98710000	6.74430000
H	-3.90490000	-2.71340000	6.07080000
C	-0.01520000	-0.52710000	3.16250000
H	0.72060000	0.17290000	3.61110000
O	-0.44560000	0.14160000	2.22720000
C	3.88020000	-1.75070000	5.88920000
C	2.90170000	-1.80120000	4.89800000
C	3.17390000	-2.41340000	3.67740000
C	4.43480000	-2.97330000	3.46100000
C	5.41070000	-2.92380000	4.44910000
C	5.13480000	-2.31010000	5.67010000
H	3.65730000	-1.26840000	6.84130000
H	1.91790000	-1.36550000	5.06090000
H	4.65160000	-3.45390000	2.50350000
H	6.39080000	-3.36490000	4.26660000
H	5.89800000	-2.26910000	6.44700000
C	2.14310000	-2.49040000	2.58630000
H	2.55790000	-2.03270000	1.66620000
H	1.95520000	-3.55530000	2.34330000
O	0.96290000	-1.84470000	2.98960000
H	0.75160000	-0.24790000	0.34230000

Species TSOPQ
HF energy= -2086.11266096
One imaginary frequency (280*i*)
Zero-point correction= 0.693144 (Hartree/Particle)
Thermal correction to Energy= 0.734924
Thermal correction to Enthalpy= 0.735868
Thermal correction to Gibbs Free Energy= 0.617526
Sum of electronic and zero-point Energies= -2085.419517
Sum of electronic and thermal Energies= -2085.377737
Sum of electronic and thermal Enthalpies= -2085.376793
Sum of electronic and thermal Free Energies= -2085.495135

Coordinates: **TSOPQ**

Ru	-0.22780000	-0.16420000	0.52430000
N	0.07340000	0.73670000	-1.22240000
N	0.49240000	1.66850000	1.09850000
N	-2.45220000	0.39270000	-0.11110000
N	-0.99420000	-1.92250000	-0.33030000
C	0.27570000	2.05440000	-1.16560000
H	0.34280000	2.64300000	-2.08080000
C	0.53520000	2.55930000	0.11320000
H	0.88800000	3.57710000	0.28730000
C	0.97690000	2.08090000	2.36420000
C	0.55180000	3.29000000	2.92680000
H	-0.20280000	3.88520000	2.40900000

C	1.06900000	3.71020000	4.14590000
H	0.72080000	4.64570000	4.58360000
C	2.01570000	2.93320000	4.81000000
H	2.42440000	3.26680000	5.76370000
C	2.42590000	1.72310000	4.25640000
H	3.15750000	1.09840000	4.76950000
C	1.90300000	1.28470000	3.04430000
H	2.22180000	0.33680000	2.60750000
C	-0.05390000	0.14220000	-2.50450000
C	0.91770000	-0.75170000	-2.95560000
H	1.78660000	-0.93740000	-2.32440000
C	0.77680000	-1.36020000	-4.19680000
H	1.54170000	-2.05230000	-4.54780000
C	-0.33220000	-1.08080000	-4.99520000
H	-0.44240000	-1.56250000	-5.96610000
C	-1.29000000	-0.17300000	-4.55300000
H	-2.15090000	0.06260000	-5.17860000
C	-1.15360000	0.44340000	-3.31130000
H	-1.89220000	1.16350000	-2.95320000
C	-3.07430000	1.64820000	-0.15150000
C	-3.96640000	2.04520000	-1.16030000
H	-4.19430000	1.37170000	-1.98780000
C	-4.54130000	3.30900000	-1.12140000
H	-5.22740000	3.61300000	-1.91150000
C	-4.23990000	4.18870000	-0.08180000
H	-4.69870000	5.17650000	-0.05430000
C	-3.33950000	3.80630000	0.91050000
H	-3.09710000	4.48530000	1.72840000
C	-2.74210000	2.55330000	0.86450000
H	-2.03200000	2.23350000	1.62710000
C	-2.96110000	-0.63080000	-0.70780000
H	-3.94770000	-0.60350000	-1.18890000
C	-2.22020000	-1.86740000	-0.75310000
H	-2.70510000	-2.76140000	-1.15830000
C	-0.40340000	-3.20700000	-0.24290000
C	-1.12960000	-4.26070000	0.32480000
H	-2.11830000	-4.06020000	0.73640000
C	-0.54100000	-5.51310000	0.46110000
H	-1.10140000	-6.32020000	0.93160000
C	0.76320000	-5.72750000	0.02440000
H	1.22360000	-6.70870000	0.13480000
C	1.48680000	-4.67450000	-0.53440000
H	2.51320000	-4.83190000	-0.86570000
C	0.91640000	-3.41290000	-0.65060000
H	1.48480000	-2.57320000	-1.04440000
C	-3.24140000	1.77880000	4.39360000
C	-2.75600000	0.72910000	3.61670000
C	-1.51730000	0.15510000	3.89060000

C	-0.77750000	0.62580000	4.97610000
C	-1.26160000	1.66820000	5.76040000
C	-2.49230000	2.25430000	5.46690000
H	-4.21250000	2.22120000	4.16330000
H	-3.32660000	0.32110000	2.78180000
H	0.18780000	0.17180000	5.20140000
H	-0.67120000	2.02790000	6.60450000
H	-2.86960000	3.07270000	6.08130000
C	-1.02810000	-0.97890000	2.98880000
H	-0.03680000	-0.55750000	2.43710000
O	-1.87730000	-1.43970000	2.12780000
C	3.64530000	-1.62540000	4.91120000
C	2.30100000	-1.91910000	4.70000000
C	1.87960000	-2.50000000	3.50180000
C	2.83030000	-2.77370000	2.51710000
C	4.17500000	-2.47110000	2.72050000
C	4.58730000	-1.89380000	3.91880000
H	3.96190000	-1.18540000	5.85840000
H	1.55610000	-1.70480000	5.46720000
H	2.50370000	-3.22810000	1.57820000
H	4.90400000	-2.68310000	1.93760000
H	5.63900000	-1.65790000	4.08180000
C	0.42740000	-2.86090000	3.29750000
H	0.20710000	-2.96710000	2.22410000
H	0.22190000	-3.84460000	3.75330000
O	-0.43580000	-1.93200000	3.89550000
H	1.22700000	-0.79470000	0.68750000

Species P
HF energy= -690.668015779
No Imaginary frequency
Zero-point correction= 0.224811 (Hartree/Particle)
Thermal correction to Energy= 0.237838
Thermal correction to Enthalpy= 0.238782
Thermal correction to Gibbs Free Energy= 0.183205
Sum of electronic and zero-point Energies= -690.443205
Sum of electronic and thermal Energies= -690.430178
Sum of electronic and thermal Enthalpies= -690.429234
Sum of electronic and thermal Free Energies= -690.484811

Coordinates: **P**

C	-1.46230000	-0.78290000	0.34440000
C	-0.08810000	-0.67090000	0.16550000
C	0.48940000	0.59070000	0.00140000
C	-0.31250000	1.73400000	0.01720000
C	-1.68490000	1.61870000	0.19730000
C	-2.25980000	0.35960000	0.36070000
H	-1.91430000	-1.76620000	0.47120000

H	0.54460000	-1.55650000	0.14890000
H	0.16480000	2.70420000	-0.11460000
H	-2.30950000	2.51140000	0.20930000
H	-3.33680000	0.26760000	0.50050000
C	1.94780000	0.77740000	-0.19650000
O	2.48880000	1.84520000	-0.35930000
O	2.61800000	-0.38870000	-0.17740000
C	4.03420000	-0.28420000	-0.35840000
H	4.42580000	0.42970000	0.38280000
H	4.24260000	0.14330000	-1.35020000
C	4.63760000	-1.64550000	-0.20310000
C	4.33590000	-2.43200000	0.91170000
C	5.53340000	-2.13110000	-1.15360000
C	4.92060000	-3.68300000	1.06870000
H	3.62880000	-2.05670000	1.65260000
C	6.12810000	-3.38050000	-0.99410000
H	5.76670000	-1.52330000	-2.02980000
C	5.82090000	-4.15950000	0.11680000
H	4.67460000	-4.29010000	1.93980000
H	6.82720000	-3.74860000	-1.74470000
H	6.28010000	-5.13990000	0.24190000

Species Q
HF energy= -1395.48932447
No Imaginary frequency
Zero-point correction= 0.467693 (Hartree/Particle)
Thermal correction to Energy= 0.495855
Thermal correction to Enthalpy= 0.496799
Thermal correction to Gibbs Free Energy= 0.407553
Sum of electronic and zero-point Energies= -1395.021632
Sum of electronic and thermal Energies= -1394.993470
Sum of electronic and thermal Enthalpies= -1394.992525
Sum of electronic and thermal Free Energies= -1395.081772

Coordinates: **Q**

Ru	-0.14250000	-0.20520000	0.42920000
N	0.37500000	0.71780000	-1.43310000
N	0.74560000	1.54780000	0.96480000
N	-2.19250000	0.14910000	-0.09590000
N	-0.86490000	-2.04500000	-0.06380000
C	0.92550000	1.91490000	-1.31860000
H	1.14930000	2.52900000	-2.19470000
C	1.18810000	2.34090000	-0.00900000
H	1.73780000	3.25500000	0.22040000
C	0.91640000	2.02520000	2.29340000
C	0.58840000	3.34720000	2.61350000
H	0.17900000	3.99800000	1.83940000
C	0.75160000	3.81480000	3.91460000

H	0.48050000	4.84320000	4.15340000
C	1.25260000	2.97420000	4.90310000
H	1.38260000	3.34080000	5.92080000
C	1.58090000	1.65720000	4.58260000
H	1.97550000	0.99080000	5.34940000
C	1.40720000	1.17830000	3.29090000
H	1.64960000	0.15040000	3.02600000
C	-0.17650000	0.33870000	-2.67210000
C	-0.07140000	-0.98970000	-3.10180000
H	0.47370000	-1.69980000	-2.48040000
C	-0.64640000	-1.38420000	-4.30310000
H	-0.54400000	-2.41820000	-4.63220000
C	-1.35300000	-0.46930000	-5.08320000
H	-1.81070000	-0.78580000	-6.01990000
C	-1.47710000	0.84880000	-4.65140000
H	-2.04020000	1.56890000	-5.24540000
C	-0.89450000	1.25410000	-3.45520000
H	-1.02690000	2.27440000	-3.09280000
C	-2.76220000	1.42940000	-0.20330000
C	-3.51860000	1.80970000	-1.31980000
H	-3.65340000	1.10370000	-2.14070000
C	-4.04180000	3.09660000	-1.39910000
H	-4.62190000	3.38710000	-2.27520000
C	-3.81890000	4.01180000	-0.37350000
H	-4.22980000	5.01870000	-0.44040000
C	-3.05600000	3.63650000	0.73240000
H	-2.87470000	4.34720000	1.53920000
C	-2.51970000	2.35850000	0.81550000
H	-1.91420000	2.04700000	1.66820000
C	-2.83650000	-0.90790000	-0.53130000
H	-3.86940000	-0.85090000	-0.88610000
C	-2.12140000	-2.12250000	-0.48370000
H	-2.55690000	-3.07210000	-0.79670000
C	-0.14790000	-3.27160000	0.02520000
C	-0.76380000	-4.43030000	0.50830000
H	-1.78900000	-4.38140000	0.87530000
C	-0.05510000	-5.62570000	0.57220000
H	-0.54190000	-6.51680000	0.96820000
C	1.26920000	-5.68160000	0.14830000
H	1.82290000	-6.61850000	0.19970000
C	1.88700000	-4.52540000	-0.32480000
H	2.92710000	-4.55370000	-0.64850000
C	1.19090000	-3.32450000	-0.37440000
H	1.67390000	-2.40570000	-0.70510000
H	-0.41220000	-0.47670000	2.00480000
H	1.17330000	-0.96690000	0.98350000

Species TSQR

HF energy= -1395.47989661
One imaginary frequency (753*i*)
Zero-point correction= 0.464745 (Hartree/Particle)
Thermal correction to Energy= 0.492679
Thermal correction to Enthalpy= 0.493624
Thermal correction to Gibbs Free Energy= 0.405411
Sum of electronic and zero-point Energies= -1395.015151
Sum of electronic and thermal Energies= -1394.987217
Sum of electronic and thermal Enthalpies= -1394.986273
Sum of electronic and thermal Free Energies= -1395.074485

Coordinates: **TSQR**

Ru	-0.19340000	-0.13180000	0.37970000
N	0.40200000	0.59720000	-1.46190000
N	0.85200000	1.51700000	0.87480000
N	-2.19040000	0.21580000	-0.19650000
N	-0.85230000	-1.96850000	-0.26980000
C	0.89980000	1.85850000	-1.40680000
H	1.10980000	2.40290000	-2.32890000
C	1.18040000	2.34550000	-0.15590000
H	1.70400000	3.28280000	0.03190000
C	1.03800000	2.01420000	2.18510000
C	0.65590000	3.32210000	2.51550000
H	0.20790000	3.95180000	1.74540000
C	0.81600000	3.79900000	3.81250000
H	0.50470000	4.81630000	4.05180000
C	1.36190000	2.98450000	4.80080000
H	1.48810000	3.35980000	5.81580000
C	1.74930000	1.68560000	4.47620000
H	2.18870000	1.04050000	5.23720000
C	1.59410000	1.20380000	3.18200000
H	1.91540000	0.19680000	2.91420000
C	-0.14540000	0.16470000	-2.68530000
C	0.10380000	-1.13300000	-3.14980000
H	0.76510000	-1.77610000	-2.56760000
C	-0.48650000	-1.58960000	-4.32230000
H	-0.27630000	-2.60130000	-4.67020000
C	-1.33550000	-0.76160000	-5.05560000
H	-1.79740000	-1.12300000	-5.97400000
C	-1.57910000	0.53510000	-4.60840000
H	-2.23820000	1.19380000	-5.17520000
C	-0.98900000	0.99710000	-3.43610000
H	-1.20110000	2.00200000	-3.06620000
C	-2.78460000	1.49440000	-0.15210000
C	-3.57220000	1.99770000	-1.19670000
H	-3.70800000	1.41700000	-2.10950000
C	-4.14200000	3.26210000	-1.09170000
H	-4.74740000	3.64560000	-1.91270000

C	-3.93100000	4.04010000	0.04360000
H	-4.37750000	5.03100000	0.11940000
C	-3.12820000	3.55020000	1.07320000
H	-2.94100000	4.15450000	1.96100000
C	-2.54920000	2.29300000	0.97320000
H	-1.89740000	1.90560000	1.75710000
C	-2.69680000	-0.74300000	-0.94200000
H	-3.62880000	-0.61860000	-1.49880000
C	-1.99470000	-1.96700000	-0.93650000
H	-2.36050000	-2.85270000	-1.45990000
C	-0.17270000	-3.19050000	-0.07470000
C	-0.86130000	-4.39670000	0.10740000
H	-1.95080000	-4.39770000	0.14060000
C	-0.15610000	-5.57790000	0.30320000
H	-0.70010000	-6.50920000	0.45900000
C	1.23720000	-5.57170000	0.31990000
H	1.78550000	-6.50040000	0.47490000
C	1.92400000	-4.37180000	0.15010000
H	3.01330000	-4.35690000	0.16580000
C	1.22650000	-3.18510000	-0.04210000
H	1.74050000	-2.23210000	-0.17990000
H	-0.65400000	-0.23540000	2.00030000
H	0.13310000	-0.91020000	1.84290000

Species R

HF energy= -1395.48444768

No Imaginary frequency

Zero-point correction= 0.468536 (Hartree/Particle)

Thermal correction to Energy= 0.496501

Thermal correction to Enthalpy= 0.497445

Thermal correction to Gibbs Free Energy= 0.410019

Sum of electronic and zero-point Energies= -1395.015912

Sum of electronic and thermal Energies= -1394.987947

Sum of electronic and thermal Enthalpies= -1394.987003

Sum of electronic and thermal Free Energies= -1395.074428

Coordinates: **R**

Ru	-0.18280000	-0.09990000	0.26490000
N	0.25890000	0.65860000	-1.54900000
N	0.61910000	1.70360000	0.73920000
N	-2.17410000	0.28850000	-0.02910000
N	-0.88870000	-1.90480000	-0.42740000
C	0.48160000	1.99530000	-1.54210000
H	0.57770000	2.53920000	-2.48240000
C	0.70310000	2.56100000	-0.30340000
H	1.03450000	3.59000000	-0.16000000
C	0.90400000	2.17990000	2.03360000
C	0.43000000	3.42290000	2.47630000

H	-0.18620000	4.02460000	1.80710000
C	0.70480000	3.86100000	3.76750000
H	0.31990000	4.82590000	4.09900000
C	1.45570000	3.07410000	4.63740000
H	1.67030000	3.42110000	5.64760000
C	1.93190000	1.83890000	4.20160000
H	2.53190000	1.21820000	4.86730000
C	1.66180000	1.39480000	2.91280000
H	2.05530000	0.44330000	2.55170000
C	-0.05130000	0.03700000	-2.77840000
C	0.52230000	-1.19770000	-3.09900000
H	1.24710000	-1.63140000	-2.41030000
C	0.15890000	-1.86010000	-4.26550000
H	0.61070000	-2.82470000	-4.49720000
C	-0.77070000	-1.29540000	-5.13730000
H	-1.05650000	-1.81840000	-6.04950000
C	-1.32410000	-0.05250000	-4.83760000
H	-2.05160000	0.39880000	-5.51280000
C	-0.96660000	0.61140000	-3.66910000
H	-1.42120000	1.56950000	-3.41090000
C	-2.73920000	1.56820000	0.15910000
C	-3.37710000	2.26680000	-0.87410000
H	-3.42700000	1.83070000	-1.87290000
C	-3.90940000	3.53020000	-0.64020000
H	-4.39520000	4.06700000	-1.45510000
C	-3.80750000	4.11570000	0.61930000
H	-4.22110000	5.10790000	0.79700000
C	-3.16330000	3.42760000	1.64640000
H	-3.06730000	3.87810000	2.63480000
C	-2.62890000	2.16630000	1.41890000
H	-2.10510000	1.62830000	2.20960000
C	-2.71140000	-0.56320000	-0.90940000
H	-3.64140000	-0.32960000	-1.43180000
C	-2.06150000	-1.79100000	-1.05760000
H	-2.48000000	-2.60650000	-1.65140000
C	-0.30170000	-3.16000000	-0.22430000
C	-1.04840000	-4.34820000	-0.18630000
H	-2.13220000	-4.30740000	-0.29320000
C	-0.41810000	-5.56320000	0.04470000
H	-1.01190000	-6.47670000	0.07770000
C	0.95940000	-5.61800000	0.25820000
H	1.44690000	-6.57510000	0.44060000
C	1.70390000	-4.44190000	0.24140000
H	2.78050000	-4.47250000	0.40740000
C	1.08270000	-3.22320000	-0.00700000
H	1.65490000	-2.29380000	-0.05830000
H	-0.53700000	-0.36240000	2.03400000
H	0.03550000	-0.94850000	1.87380000
